# Polarisation effects on the H-bond acceptor properties of secondary amides†

**DOI:** 10.1039/d3sc03823h

**Published:** 2023-09-29

**Authors:** Fergal E. Hanna, Alexander J. Root, Christopher A. Hunter

**Affiliations:** a Yusuf Hamied Department of Chemistry, University of Cambridge Lensfield Road Cambridge CB2 1EW UK herchelsmith.orgchem@ch.cam.ac.uk

## Abstract

H-bonding interactions in networks are stabilised by cooperativity, but the relationship between the chemical structures of the interacting functional groups and the thermodynamic consequences is not well-understood. We have used compounds with an intramolecular H-bond between a pyridine H-bond acceptor and an amide NH group to quantify cooperative effects on the H-bond acceptor properties of the amide carbonyl group. ^1^H NMR experiments in *n*-octane confirm the presence of the intramolecular H-bond and show that this interaction is intact in the 1 : 1 complex formed with perfluoro-*tert*-butanol (PFTB). UV-vis absorption titrations were used to measure the relationship between the association constant for formation of this complex and the H-bond acceptor properties of the pyridine involved in the intramolecular H-bond. Electron-donating substituents on the pyridine increase the strength of the intermolecular H-bond between PFTB and the amide. There is a linear relationship between the H-bond acceptor parameter *β* measured for the amide carbonyl group and the H-bond acceptor parameter for the pyridine. The cooperativity parameter *κ* determined from this relationship is 0.2, *i.e. β* for an amide carbonyl group is increased by one fifth of the value of *β* of an acceptor that interacts with the NH group. This result is reproduced by DFT calculations of H-bond parameters for the individual molecules in the gas phase, which implies that the observed cooperativity can be understood as polarisation of the electron density in the amide π-system in response to formation of a H-bond. The cooperativity parameter *κ* measured for the secondary amide H-bond donor and H-bond acceptor is identical, which implies that polarisation of an amide mediates the interaction between an external donor or acceptor in a reciprocal manner.

## Abstract 2

An intramolecular H-bond between a pyridine and an amide NH group increases the H-bond acceptor strength of the amide carbonyl oxygen. Polarisation of the amide group is directly proportional to the polarity of the pyridine H-bond acceptor.

## Graphical abstract

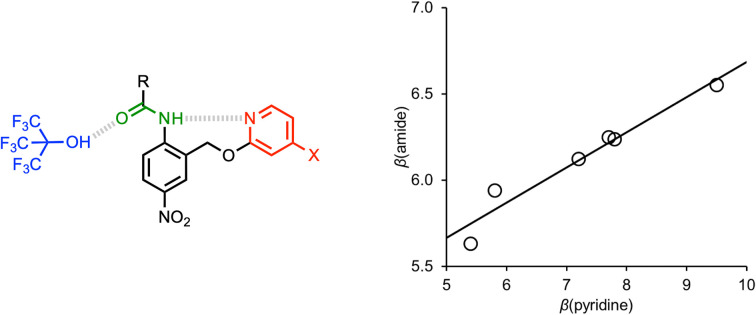

## Introduction

Non-covalent interactions play a central role in determining the functional properties of biomolecules,^1^ materials^2^ and supramolecular systems,^3^ as well as in the design of new drugs and catalysts. The thermodynamic properties of non-covalent interactions can be predicted, to a first approximation, according to the properties of the individual, isolated molecules.^4^ However for some functional groups, polar interactions such as H-bonding can alter the charge density, leading to cooperative effects in interaction networks.^1–3^ For example, supramolecular polymerisation of *N*-methylacetamide is cooperative, and formation of the first H-bond in the chain is less favourable than subsequent interactions, both in terms of free energy and enthalpy.^5–7^ Calculations at varying theory levels predict that upon forming a H-bond the charge density of the amide is polarised to make the NH more positive and carbonyl oxygen more negative, leading to an increase in the stability of subsequent H-bonding interactions with these groups.^8–16^ Spectroscopic analysis of gas phase complexes confirm these predictions,^17^ and X-ray crystal structure data show that formation of a H-bond lengthens the C–O bond and shortens the C–N bond in an amide.^18,19^ These experiments are consistent with polarisation of the electron density towards the oxygen, which increases C–N double bond character and decreases C–O double bond character. The development of an understanding of the magnitude of cooperative effects is of particular significance for biological systems, because of the prevalence of networks of H-bonded secondary amides in protein structures.

Although it is known that this kind of allosteric cooperativity can increase the stability of H-bonding interactions with amide groups by an order of magnitude,^5–7,18–20^ the relationship between the properties of individual functional groups and the magnitude of cooperative effects in H-bonded networks is not well-understood. We have recently developed an approach that allows quantification of the effect of an intramolecular H-bond on the thermodynamic properties of an intermolecular H-bond. By tuning the polarity of the groups involved in the intramolecular H-bond, it is possible to build up structure–activity relationships to quantify cooperative effects in multiply H-bonded networks. For example, we have shown that formation of an intramolecular H-bond between a phenol H-bond donor and an amide carbonyl oxygen stabilises intermolecular H-bonds formed with the amide NH group. The increase in H-bond donor strength of the amide NH was directly proportional to the H-bond donor strength of the phenol.^21^ Here we reverse this system and investigate the effect of an intramolecular H-bond between a pyridine H-bond acceptor and an amide NH group on the H-bond acceptor properties of the amide carbonyl.

**Fig. 1:**
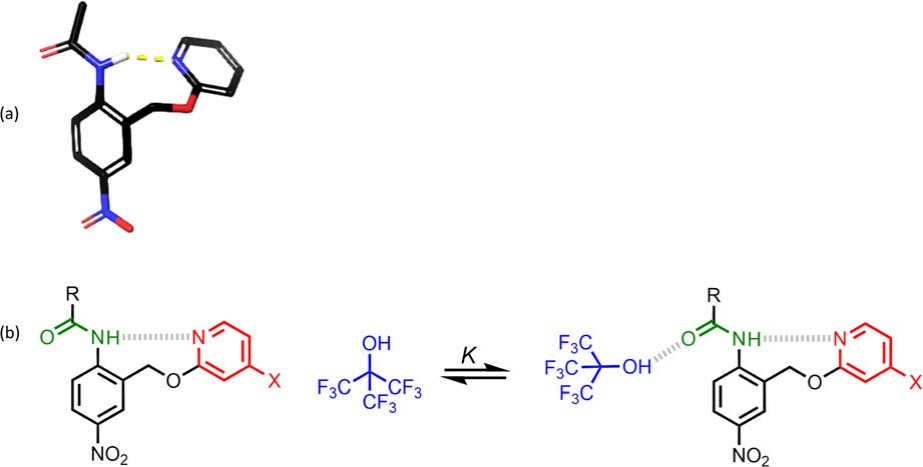
(a) Lowest energy conformation of *p*-nitroacetanilide obtained using a molecular mechanics conformational search (OPLS4 with chloroform solvation in Macromodel) and optimised with DFT (6-31G* B3LYP). The yellow dotted line is a H-bond. (b) Interaction of a H-bonded amide group (green) with perfluoro-*tert*-butanol (PFTB). X is a substituent that modulates the H-bond acceptor properties of the pyridine (red), and R is a solubilising group.

Fig. 1(a) shows a platform for quantification of cooperative effects on the H-bond acceptor properties of an amide carbonyl group. Molecular mechanics conformational searches and density functional theory (DFT) calculations indicate that an intramolecular H-bond will be formed between the pyridine acceptor and the amide NH group. The H-bond acceptor properties of the pyridine can be varied over a wide range by addition of substituents (X in Fig. 1(b)), and the methylene spacer ensures that there is no through bond conjugation between the pyridine and amide.^22^ The relationship between pyridine substituent X and the H-bond acceptor properties of the amide can be quantified by measuring association constants (*K*) for formation of 1 : 1 complexes with a standard H-bond donor in a non-polar solvent. Here we use perfluoro-*tert*-butanol (PFTB) as the H-bond donor, because it is an exceptionally strong H-bond donor and exceptionally weak H-bond acceptor, ensuring that the main site of interaction is with the amide carbonyl group as shown in Fig. 1(b).

## Results

### Synthesis

**Scheme 1:**
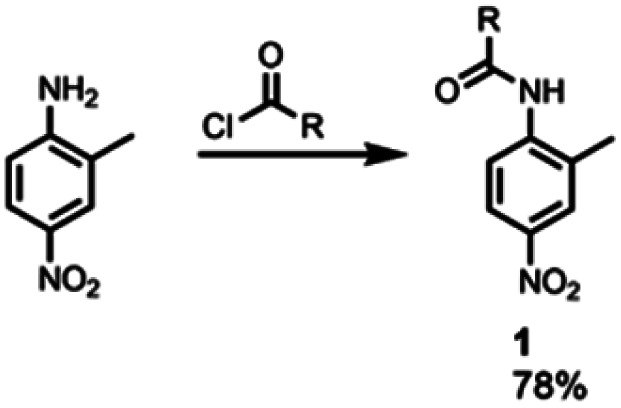
Synthesis of 1. R = 3-heptyl.

Reference compound 1, which does not have an intramolecular H-bonding interaction, was synthesised by the condensation of commercially available 2-methyl-4-nitroaniline and 2-ethylhexanoyl chloride (Scheme 1).

**Scheme 2:**
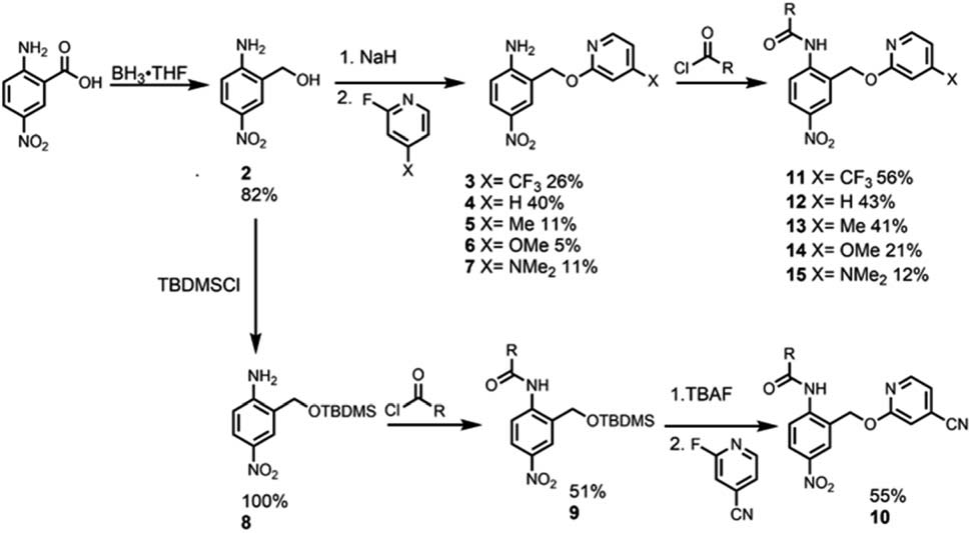
Synthesis of compounds 10–15. R = 3-heptyl.

Synthesis of the analogues that contain a pyridine substituent was carried out using the route shown in Scheme 2. Reduction of commercially available 2-amino-5-nitrobenzoic acid to compound 2 provided a handle for S_N_Ar reactions with 2-fluoropyridine derivatives to form compounds 3–7. Amide coupling reactions with 2-ethylhexanoyl chloride gave amides 11–15. The yield of compound 10 (X = CN) obtained by this route was very low, but reversing the order of the amide coupling and S_N_Ar steps gave 10 in good yield.

### Intramolecular H-bonding

**Fig. 2:**
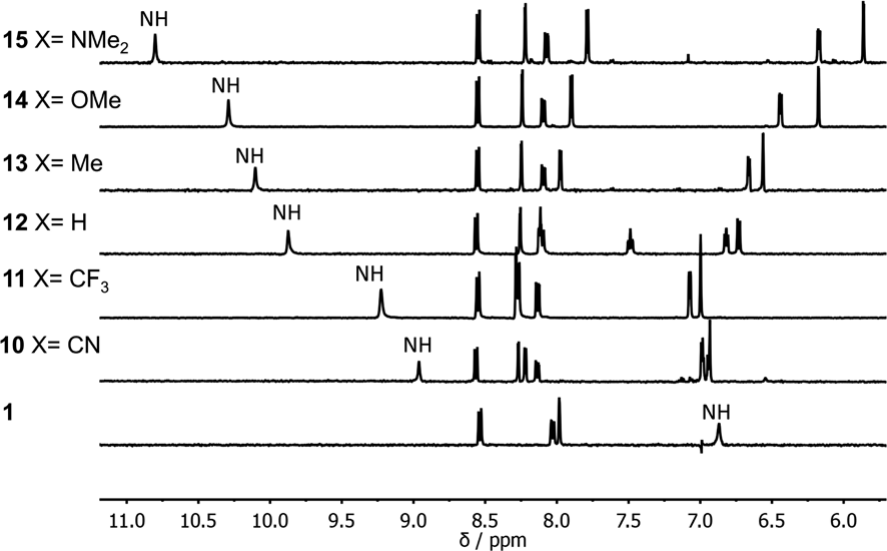
Partial 400 MHz ^1^H NMR spectra of compounds 1 and 10–15 (0.2–1.0 mM) recorded in *n*-octane at 298 K with WET solvent suppression. The signal due to the amide NH is highlighted.

Fig. 2 shows ^1^H NMR spectra of compounds 1 and 10–15 recorded in *n*-octane. For compounds 10–15, the presence of the pyridine substituent leads to an increase of over 2 ppm in the chemical shift of the signal due to the amide NH group compared with compound 1. ^1^H NMR dilution experiments showed no evidence of any intermolecular interactions at these concentrations (see ESI†). The large increases in the NH chemical shift relative to 1 therefore provide strong evidence for the presence on an intramolecular H-bonding interaction between the pyridine nitrogen and the amide NH in each of compounds 10–15. There are also significant differences in the chemical shifts of the signal due to the NH proton in compounds 10–15. The largest chemical shifts are observed for the most electron-donating X substituents, and the values correlate with the H-bond acceptor parameters (*β*) of the corresponding 4-X-pyridines (see Table 1, *R*^2^ = 0.98). This observation is consistent with the presence an intramolecular H-bond that is sensitive to the H-bond acceptor properties of the pyridine nitrogen.

**Table 1:** Association constants for formation of 1 : 1 complexes with PFTB measured by UV/vis absorption titrations and limiting ^1^H NMR chemical shifts of the signal due to the amide NH proton (ppm) in *n*-octane at 298 K^a^

Compound	X	*β*(pyridine)^b^	*K*/M^−1^	*δ*(free)	*δ*(bound)
10	CN	5.4	160 ± 30	8.96	9.45
11	CF_3_	5.8	260 ± 60	9.22	9.88
12	H	7.2	340 ± 40	9.87	10.63
13	Me	7.7	410 ± 60	10.10	10.84
14	OMe	7.8	400 ± 40	10.29	10.84
15	NMe_2_	9.5	640 ± 30	10.80	11.67

a Errors are quoted as two standard deviations based on at least three different experiments.

b Literature values from ref. 23, except for the value for 4-(trifluoromethyl)pyridine, which was calculated from the linear relationship between the Hammett substituent constant and *β* for 4-X-pyridines (see ESI).

### Intermolecular H-bonding

**Fig. 3:**
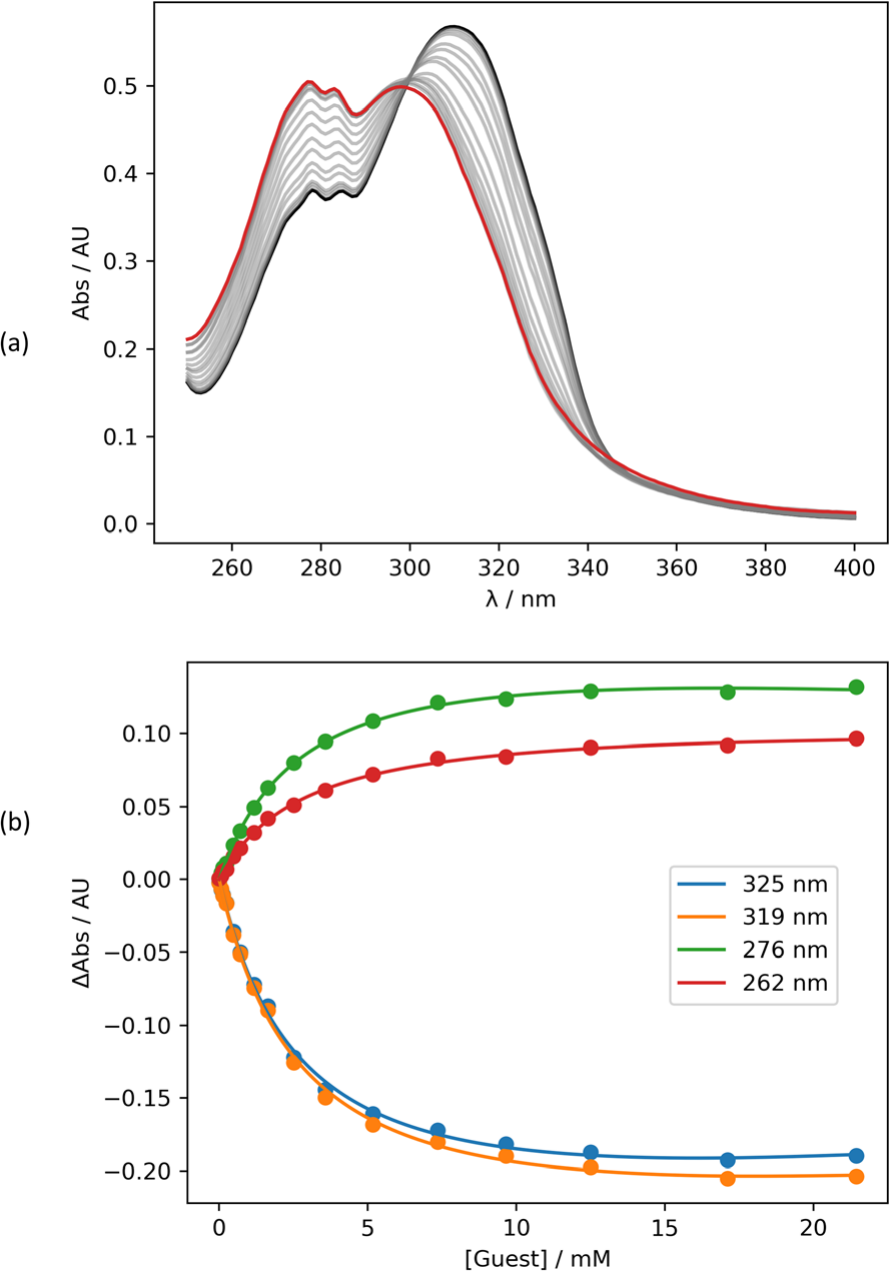
(a) UV/vis absorption spectra for the titration of PFTB into 13 (37 μM in *n*-octane, at 298 K). The UV/vis spectrum of 13 and the final point of the titration are reported in black and in red, respectively. (b) The lines show the best fit of the absorbance at four different wavelengths (points) to a 1 : 1 binding isotherm with a linear correction for the absorption due to the guest.

Intermolecular interactions between the amide carbonyl oxygen and PFTB were investigated using UV/vis absorption titrations in *n*-octane at 298 K. Fig. 3 shows data for the titration of PFTB into 13 (see ESI† for the other compounds). Addition of PFTB leads to disappearance of the absorption band at 320 nm and the appearance of a new band at 300 nm. The titration data fit well to a 1 : 1 binding isotherm in all cases, and the resulting association constants are summarised in Table 1. The largest association constant was observed for the most electron-donating pyridine substituent, X = NMe_2_, and the smallest association constant was observed for the most electron-withdrawing substituent, X = CN. To confirm that H-bonding does not occur at the nitro group of the amide compounds, UV/vis absorption titrations were also carried out using 2-methyl-4-nitroaniline as the host (see ESI†). In this case, the data also fit well to a 1 : 1 binding isotherm, but the association constant is an order of magnitude lower than the values measured for the amides (*K* = 28 ± 2 M^−1^). In addition, the UV/vis absorption spectrum of the 2-methyl-4-nitroaniline·PTFB complex is red-shifted relative to 2-methyl-4-nitroaniline, whereas for the amide complexes, the H-bonding interaction with PFTB leads to a blue shift in all cases (see ESI†).

**Fig. 4:**
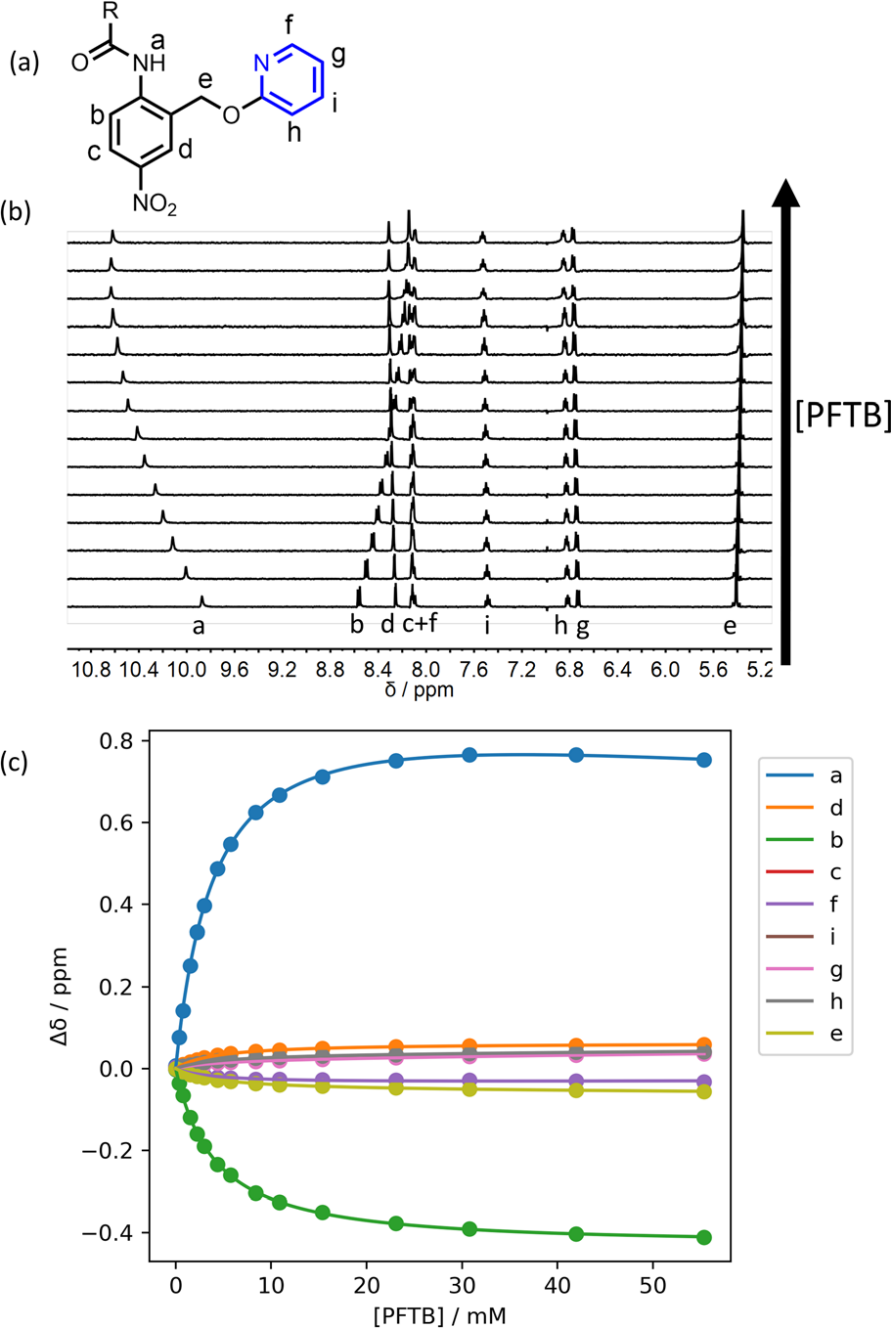
(a) Proton labelling scheme for compound 12. (b) 400 Mz ^1^H NMR spectra for titration of PFTB into 12 (0.21 mM) in *n*-octane at 298 K. (c) The lines show the best fit of the chemical shift data (points) to a 2 : 1 binding isotherm.

To test whether intermolecular H-bonding with PFTB competes with the intramolecular H-bond, ^1^H NMR titrations were carried out in *n*-octane. Fig. 4 shows the data for titration of PFTB into compound 12 in *n*-octane at 298 K. A large increase in the chemical shift of the signal due to the amide NH proton was observed. For each of compounds 10–15, the limiting complexation-induced change in the ^1^H NMR chemical shift of the signal due to the amide NH proton was more than +0.5 ppm for formation of the 1 : 1 complex with PFTB (Table 1). In contrast, when an ^1^H NMR titration was carried out with compound 1, which does not have an intramolecular H-bond, the limiting complexation-induced change in the chemical shift of the signal due to the amide NH proton was much smaller (+0.2 ppm). These results suggest that the intramolecular amide-pyridine interaction is enhanced rather than disrupted by the intermolecular H-bond in the PFTB complexes of compounds 10–15. The other signal that moves significantly in the ^1^H NMR titration experiments is the signal due to proton b, which is *ortho* to the amide group (Fig. 4(b)). Fig. 1(b) shows that this proton is in close proximity to the guest when PFTB forms a H-bond with the amide carbonyl oxygen, providing further evidence for the structure of complex.

**Fig. 5:**
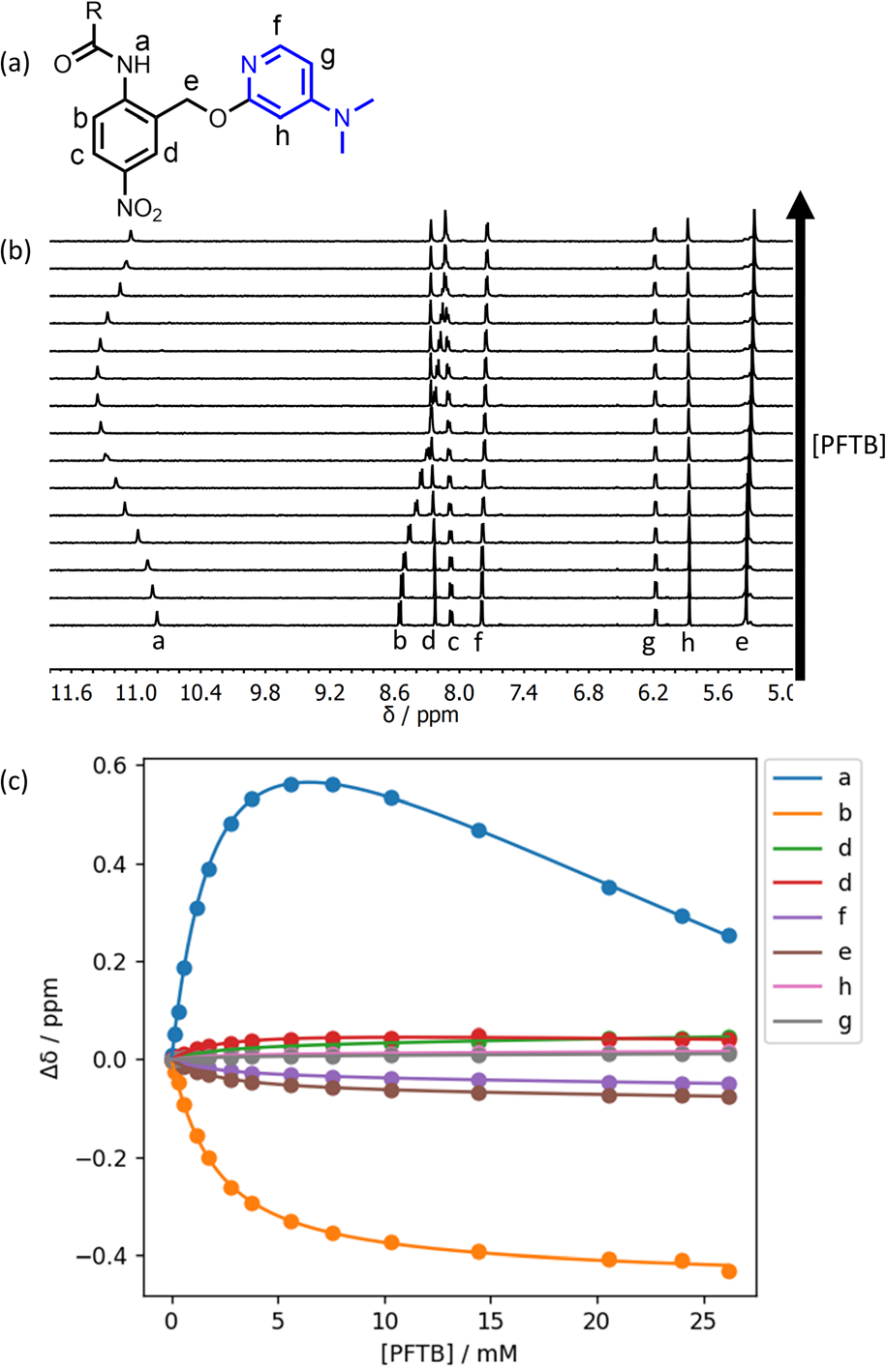
(a) Proton labelling scheme for compound 15. (b) 400 Mz ^1^H NMR spectra for titration of PFTB into 15 (0.65 mM) in *n*-octane at 298 K. (c) The lines show the best fit of the chemical shift data (points) to a 2 : 1 binding isotherm.

The titration data shown in Fig. 4 for compound 12 fit slightly better to a 2 : 1 binding isotherm than a 1 : 1 binding isotherm, giving a value of *K*_1_ that is similar to the value measured by UV/vis absorption spectroscopy (260 M^−1^) and a much weaker second binding interaction (*K*_2_ = 5 M^−1^). The weak second binding interaction was more clearly visible in the titration of PFTB into compound 15 (Fig. 5). The chemical shift of the signal due to the amide NH proton initially increased on addition of PFTB, as observed for the other compounds. However for guest concentrations greater than 10 mM, the chemical shift started to decrease as more PFTB was added. The limiting ^1^H NMR chemical shift of the signal due to the NH proton in the 2 : 1 complex is 8.03 ppm, which is significantly lower than the value measured in the free state (10.80 ppm). This result suggests that the weaker second binding interaction involves breaking of the intramolecular H-bond, so that the PTFB can interact with the pyridine nitrogen H-bond acceptor. Again, the value of *K*_1_ was similar to the value measured by UV/vis absorption spectroscopy (610 M^−1^), and the value of *K*_2_ was much lower (8 M^−1^). The second binding event has a more dramatic effect on the appearance of the isotherm in this case, because the value of *K*_2_ measured for 15 is larger than the value measured for 12.

The increased competition between intermolecular and intramolecular H-bonding observed for compound 15 can be rationalised based on the H-bond properties of the functional groups involved. The pyridine nitrogen in compound 15 is a better H-bond acceptor than the pyridine nitrogen in compound 12 (*β* = 9.5 and 7.2 respectively), and PFTB is a better H-bond donor than the amide NH group (*α* = 4.9 and 3.3 respectively).^23–25^ Thus although the intramolecular H-bond in compound 15 is stronger than the intramolecular H-bond in compound 12, the pyridine substituents have a more dramatic effect on the strength of the intermolecular H-bond than on the intramolecular H-bond, because PFTB is a better H-bond donor than the amide NH group. As a consequence, PFTB starts to compete with the intramolecular H-bond in compound 15 at lower concentrations than for compound 12.

## Discussion

The ^1^H NMR spectra show that there is an intramolecular H-bond between the amide NH donor and pyridine nitrogen acceptor in compounds 10–15 in *n*-octane solution, and the ^1^H NMR titration data show that this intramolecular H-bond is also present in the 1 : 1 complexes formed with PFTB. The intramolecular H-bond can only be broken by addition of a very large excess of PFTB, because although PFTB in a better H-bond donor than an amide, intermolecular interactions are less favourable than intramolecular interactions. The association constants for formation of the 1 : 1 PFTB complexes measured by UV/vis absorption titrations show that substituents on the pyridine group have a significant effect on the strength of the intermolecular H-bond formed between PFTB and the amide carbonyl oxygen acceptor. There is a correlation between the H-bond acceptor parameters of the pyridine group and the association constants listed in Table 1. The strength of the intermolecular H-bond increases with the strength of the intramolecular H-bond. This positive cooperativity indicates that the intramolecular H-bond modulates the polarity of the amide group, presumably by changing the electron distribution in the π-system.

The association constants in Table 1 can be used to determine H-bond acceptor parameters for the amide group in compounds 10–15 using eqn (1).^4^

1 Δ*G*°/kJ mol^−1^ = −*RT* ln *K* = −(*α* − *α*_S_)(*β* − *β*_S_) + 6

where *α* is H-bond donor parameter for PFTB (4.9), *β* is the H-bond acceptor parameter of the amide group, and *α*_S_ and *β*_S_ are the H-bond parameters of the solvent (1.2 and 0.6 respectively for *n*-octane).^22,26^

**Fig. 6:**
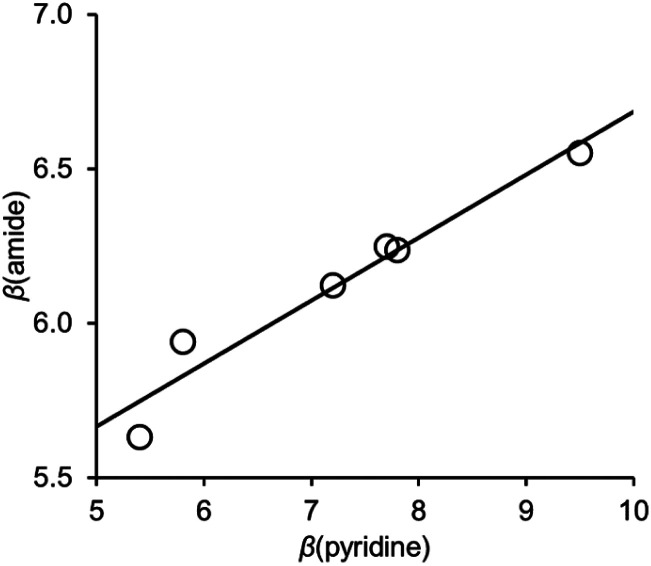
Relationship between the H-bond acceptor properties of the amide group in compounds 10–15, *β*(amide), and the H-bond acceptor properties of the corresponding 4-X-pyridines, *β*(pyridine). The best fit line is *y* = 0.20*x* + 4.6 (*R*^2^ = 0.94).

Fig. 6 shows the relationship between the value of *β*(amide) measured for compounds 10–15 and the value of *β*(pyridine), the H-bond acceptor parameter for the corresponding 4-X-pyridines. There is a linear relationship with a positive slope of 0.2. We have previously measured the effect of an intramolecular interaction between a phenol H-bond donor and an amide carbonyl oxygen on the H-bond donor properties of the amide NH donor interacting with a phosphine oxide (R_3_PO).^21^ A linear relationship was found between the value of *α* for the phenol and the value of *α* for the amide, and the slope of this correlation was also 0.2.

The change in H-bond parameters can also be expressed as change in the free energy for formation of the intermolecular H-bond of an amide acceptor with PFTB (eqn (2)) or an amide donor with R_3_PO (eqn (3)).

2 ΔΔ*G*°/kJ mol^−1^ = −*α*_PFTB_Δ*β* = −0.2*α*_PFTB_*β*_pyridine_

3 ΔΔ*G*°/kJ mol^−1^ = −Δ*αβ*_R_3_PO_ = −0.2*α*_phenol_*β*_R_3_PO_

**Fig. 7:**
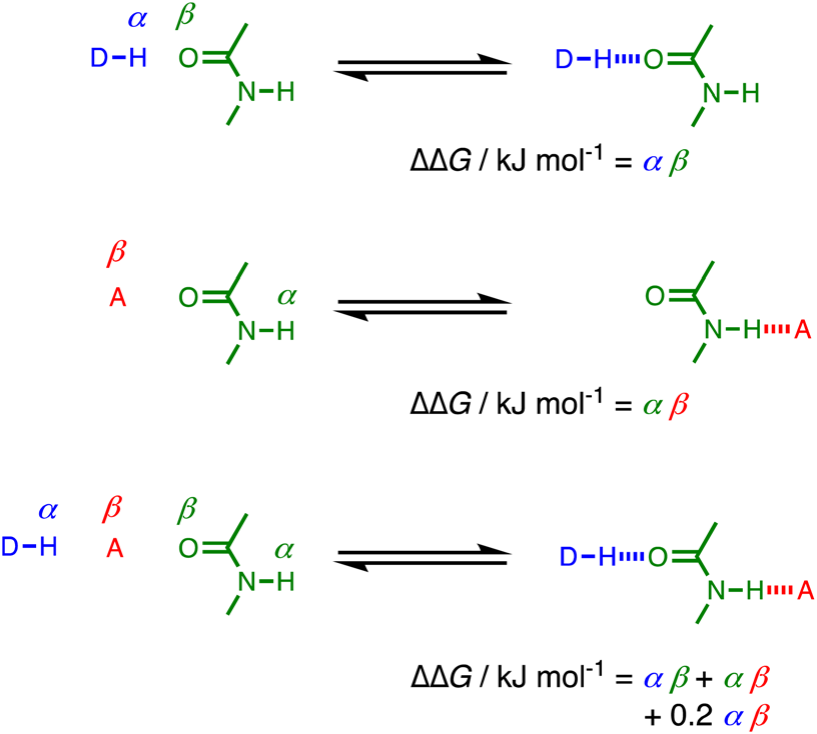
Comparison of H-bond interactions in singly H-bonded complexes and a cooperative doubly H-bonded network mediated by an amide group.

The fact that the cooperativity factor of 0.2 is the same in eqn (2) and (3), regardless of whether the amide acts a H-bond donor or acceptor, suggests that cooperativity in these systems can be considered as a long range interaction between the two external groups that interact with the amide. Fig. 7 illustrates the difference between two single H-bonding interactions with an amide and a cooperative interaction mediated by an amide group. The increase in H-bond strength in the cooperative doubly H-bonded complex is equivalent to a direct H-bond between the external donor DH and acceptor A multiplied by a cooperativity factor, *κ* = 0.2.

**Fig. 8:**
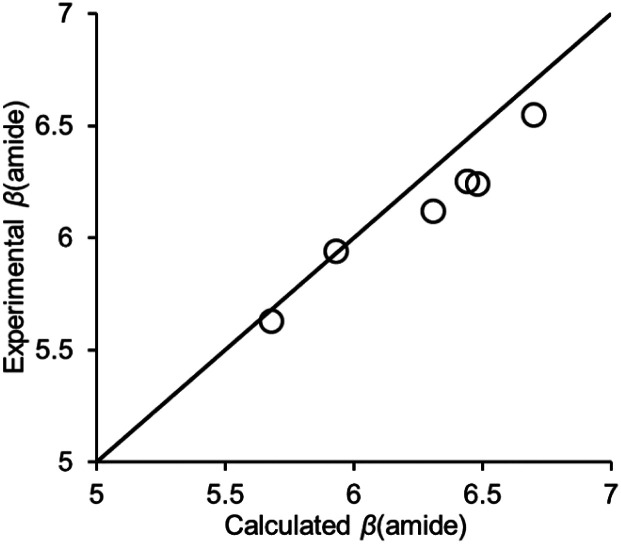
Comparison of calculated and experimental values of *β*(amide) for compounds 10–15. The line is *y* = *x* (RMSE = 0.16).

It is possible using density functional theory (DFT) in the gas phase to calculate H-bond acceptor parameters for amide groups from the minimum in the molecular electrostatic potential calculated on the 0.0300 e Bohr^−3^ electron density isosurface^27^ (see ESI†). Fig. 8 compares the calculated and experimental values of *β*(amide) for compounds 10–15. The agreement is excellent (RMSE = 0.16). The DFT calculations are based on the properties of the isolated molecule rather than the complex formed with PFTB, which suggests that the cooperativity observed for the H-bond network illustrated in Fig. 7 can be understood based on polarisation of the electron density in the amide group due to the intramolecular H-bond.

## Conclusions

Compounds with an intramolecular H-bond between a pyridine H-bond acceptor and an amide NH group have been used to quantify cooperative effects on the H-bond acceptor properties of the amide carbonyl oxygen. ^1^H NMR experiments in *n*-octane confirm the presence of the intramolecular H-bond and show that this interaction is intact in the 1 : 1 complex formed with perfluoro-*tert*-butanol (PFTB). UV-vis absorption titrations were used to measure the relationship between the association constant for formation of this complex and the H-bond acceptor properties of the pyridine involved in the intramolecular H-bond.

Electron-donating substituents on the pyridine increase the strength of the intermolecular H-bond between PFTB and the amide, and there is a linear relationship between the H-bond acceptor parameter *β* measured for the amide carbonyl oxygen and the H-bond acceptor parameter for the pyridine. The cooperativity parameter *κ* measured from this relationship is 0.20, which is identical to the cooperativity parameter previously reported for the H-bond donor parameter of an amide NH group involved in intramolecular H-bonding interactions. This result suggests that cooperativity in networks of H-bonded amides can be considered as a long range interaction between the H-bond donor that interacts with the amide carbonyl oxygen and the H-bond acceptor that interacts with the amide NH group. The interaction between these external H-bond donor and acceptor groups is moderated by the intervening amide, so that increase in stability due to the cooperative effect is equivalent to one fifth of a direct donor–acceptor interaction. The experimental results are reproduced well by DFT calculations of H-bond parameters for the individual molecules in the gas phase, which implies that the observed cooperativity can be understood as polarisation of the electron density in the amide π-system in response to formation of a H-bond.

The cooperativity parameter measured for amides (*κ* = 0.20) is significantly lower than the corresponding parameter that we measured previously for phenols (*κ* = 0.33).^22^ This result suggests that H-bond cooperativity and polarisation effects depend strongly on chemical structure, and the approach described here could be used to experimentally characterise a range of different functional groups. However, the agreement between H-bond parameters measured by experiment and the parameters derived from DFT calculations indicates that theoretical methods might also be useful for determining cooperativity parameters for other functional groups.

The experiments on cooperativity in chains of H-bonded phenols also have implications for understanding how cooperativity might affect chains of H-bonded secondary amides. The phenol results imply that H-bond cooperativity is dominated by pairwise interactions between nearest neighbours in a chain, so that the maximum increase in the H-bond parameters at the end of a long chain is given by 1/(1 − *κ*).^22^ In the case of secondary amides, the maximum increase in the H-bond parameters of the chain ends would therefore be 25%, which is not much larger the 20% increase due to a single H-bond between two amides.

## Data availability

All supporting data is provided in the ESI.†

## Author contributions

The manuscript was written through contributions of all authors.

## Conflicts of interest

There are no conflicts to declare.

## Supplementary Material

SC-014-D3SC03823H-s001
