# Supplementary material for: Polarisation effects on the H-bond acceptor properties of secondary amides
Source: Chem Sci. 2023 Sep 29;14(40):11151–7. doi: 10.1039/d3sc03823h (PMC10583693; doi:10.1039/d3sc03823h)
Supplement: SC-014-D3SC03823H-s001 [file SC-014-D3SC03823H-s001.pdf]

Electronic Supplementary Material (ESI) for Chemical Science.
This journal is © The Royal Society of Chemistry 2023

Polarisation Effects on the H-bond Acceptor Properties of Secondary Amides

Fergal E. Hanna, Alexander J. Root, and Christopher A. Hunter*

Yusuf Hamied Department of Chemistry, University of Cambridge, Lensfield Road, Cambridge CB2 1EW, UK.

Supplementary Information

Table of Contents

Materials and methods 2

Relationship between σ_P and β(pyridine) 3

Synthesis and characterisation 4

UV/Vis absorption spectroscopy and ^{1}H NMR titrations 40

NMR Titration Shift Changes 60

Computational calculations 61

References 62

Materials and methods

All reagents were purchased from commercial sources (Sigma Aldrich UK, Acros, Tokyo Chemical Industry, Alfa Aesar and FluoroChem) and were used as received without any further purification unless stated. Dry solvents were obtained by means of a Grubbs solvent purification system.

Flash chromatography was done with an automated system (Combiflash Companion) using pre-packed cartridges of silica (50 µm PuriFlash® column) or basic alumina (45 µm PuriFlash® column)

The LC-MS analysis of samples was performed using Waters Acquity H-class UPLC coupled with a single quadrupole Waters SQD2. ACQUITY UPLC CSH C18 Column, 130 Å, 1.7 µm, 2.1 mm X 50 mm was used as the UPLC column for all samples. The conditions of the UPLC method are as follows: Solvent A: Water +0.1% Formic acid; Solvent B: Acetonitrile +0.1% Formic acid; Gradient of 0-2 minutes 5% - 100%B + 1 minute 100% B with re-equilibration time of 2 minutes. Flow rate: 0.6 ml/min; column temperature of 40°C; injection volume of 2 µL. The signal was monitored with MS-ES+, MS-ES-, and UV/Vis absorption at 254 nm or at 290 nm.

^{1}H-NMR and ^{13}C-NMR were recorded on a 400 MHz Bruker spectrometer. The reference values used for the chemical shifts of the various spectra are reported in the literature[1]. The splitting pattern is indicated with the following abbreviations: s for singlet, d for doublet, t for triplet, q for quartet, p for pentet and m for multiplet.

FT-IR spectra were collected with a Bruker ALPHA FT-IR Spectrometer.

UV/Vis spectra were recorded with an Agilent UV/Vis Cary 60 spectrophotometer.

Melting points were measured in a Mettler Toledo MP50 Melting Point System.

Relationship between σ_P and β(pyridine)

X	σ_p[a]	β(pyridine)[b]
CN	0.66	5.4
Cl	0.23	6.5
CH=CH2	-0.01	7.4
Ph	-0.02	7.4
^{i}Pr	-0.15	7.4
Et	-0.07	7.4
Me	-0.07	7.7
OMe	-0.27	7.8
NMe_2	-0.83	9.3
H	0	7.2

Table S.1 – *Para* Hammett constants, σ_P, for a substituent X and the corresponding β(pyridine) value for the 4-X-pyridine.

[a]Literature values taken from Ref 2

[b]Literature values taken from Ref 3

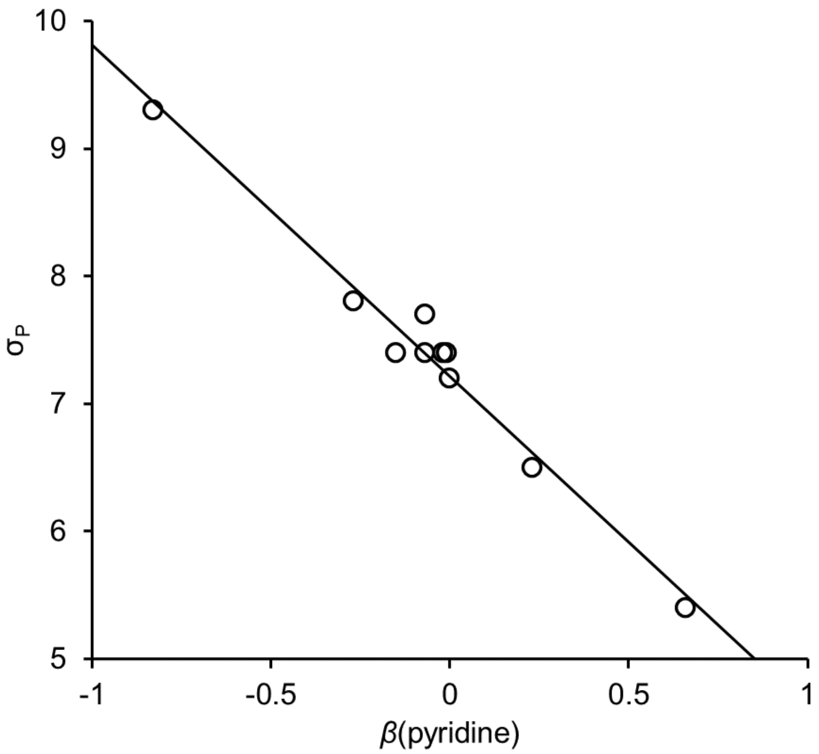

Figure S.1 – Plot of β(pyridine) against σ_P for 4-X-pyridine, where X corresponds to the functional groups listed in Table S.4. The line of best fit is Y = -2.6 X + 7.2. R^2 = 0.97

Synthesis and characterisation

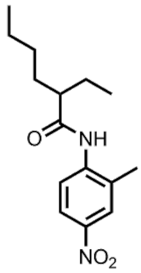

2-ethyl-N-(4-nitro-2-methylphenyl)hexanamide, **1**. 4-nitro-2-methylaniline (152 mg, 1.00 mmol) was dissolved in dry DCM (5 mL) under a nitrogen atmosphere. NEt_3 (0.2 mL, 1.15 mmol) and 2-ethylhexanoylchloride (0.4 mL, 2.87 mmol) were added and the reaction stirred for 24 hours. The reaction mixture was diluted with DCM (50 mL) and washed with HCl (1 M, 2 x 25 mL) and $NaHCO_3$ (25 mL). The organic layer was dried with $MgSO_4$, and the solvent removed under reduced pressure to obtain the desired product as a yellow solid (218 mg, 0.783 mmol, 78%)

ν_{max} (film) cm^{-1} : 3268 (N-H), 2960 (C-H), 2924 (C-H), 2858 (C-H), 1656 (C=O), 1587 (C=C), 1531 (C=C), 1503, 1355, 1274, 743

^{1}H NMR (400 MHz, $CDCl_3$) δ_H ppm: 8.36 (dd, J = 8.8, 2.3 Hz, 1H), 8.13 – 8.07 (m, 2H), 7.16 (s, 1H), 2.37 (d, J = 2.4 Hz, 3H), 2.20 (dq, J = 12.7, 6.9, 5.5 Hz, 1H), 1.73 (dt, J = 14.8, 7.4 Hz, 2H), 1.59 (h, J = 8.1, 7.5 Hz, 2H), 1.34 (dd, J = 6.9, 3.1 Hz, 4H), 0.99 (td, J = 7.5, 2.2 Hz, 3H), 0.90 (dt, J = 7.9, 3.9 Hz, 3H).

^{13}C{^{1}H} NMR (101 MHz, $CDCl_3$) δ_C ppm: 174.6, 143.5, 142.0, 127.3, 125.8, 125.1, 123.1, 121.2, 51.2, 32.7, 30.0, 26.4, 22.9, 17.9, 14.1, 12.2.

HRMS: calc for $C_{15}H_{23}N_2O_3^+$ $[M+H]^+$: 297.1709, found: 279.1718

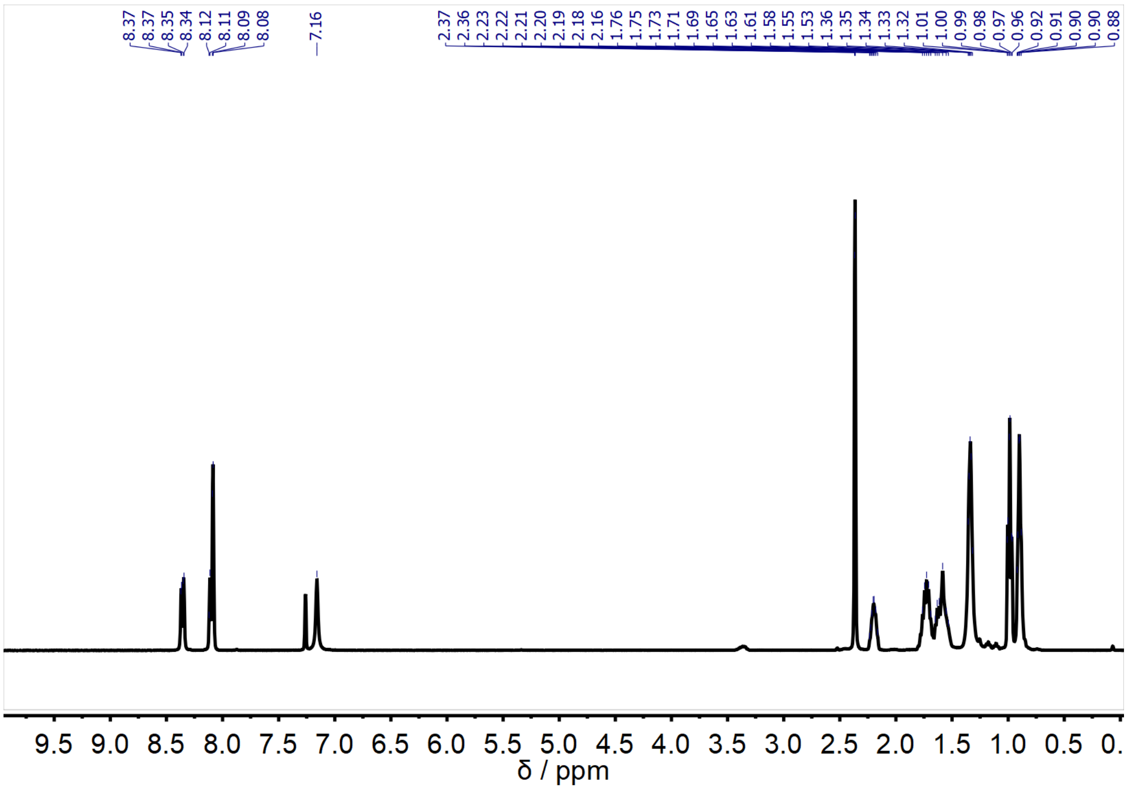

Figure S.2 – ^{1}H NMR spectrum of **1** in $CDCl_3$ (δ 0 – 10 ppm).

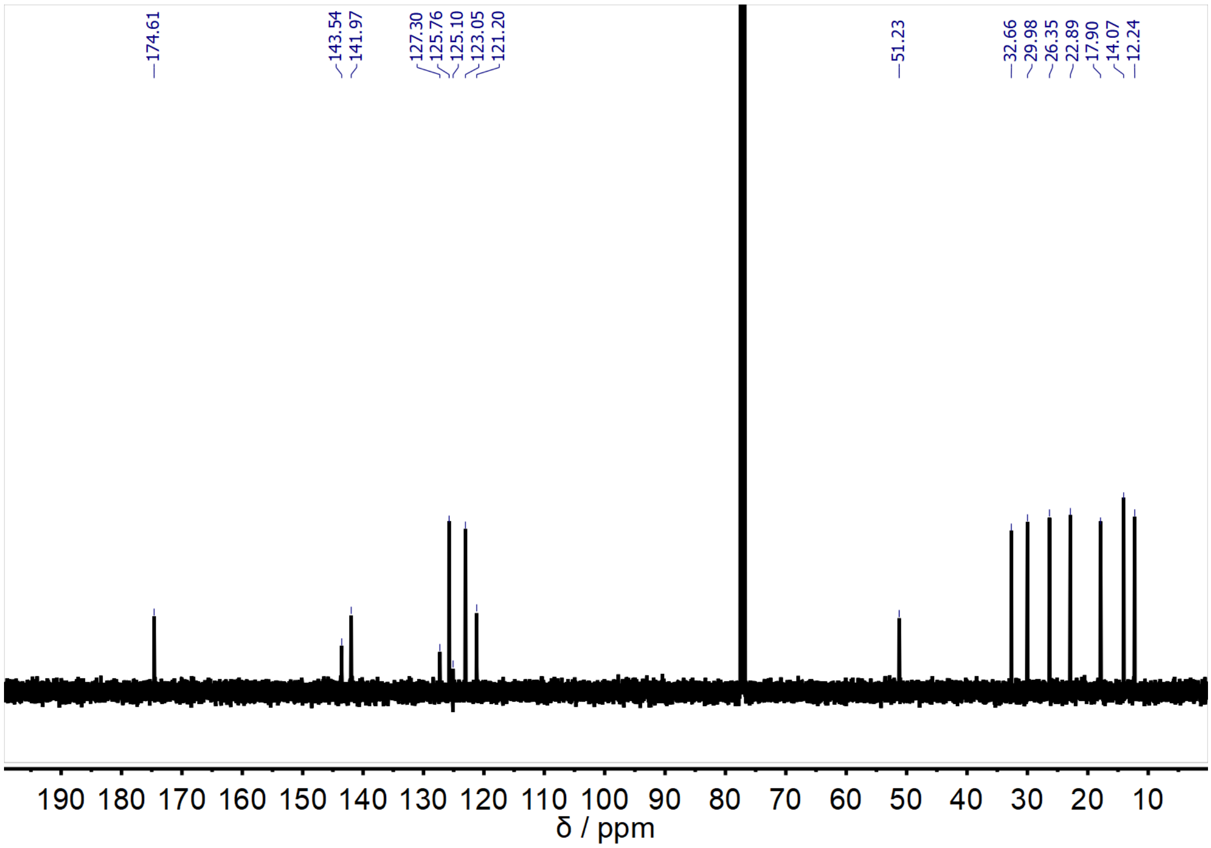

Figure S.3 – ^{13}C NMR spectrum of **1** in $CDCl_3$ (δ 0 – 200 ppm).

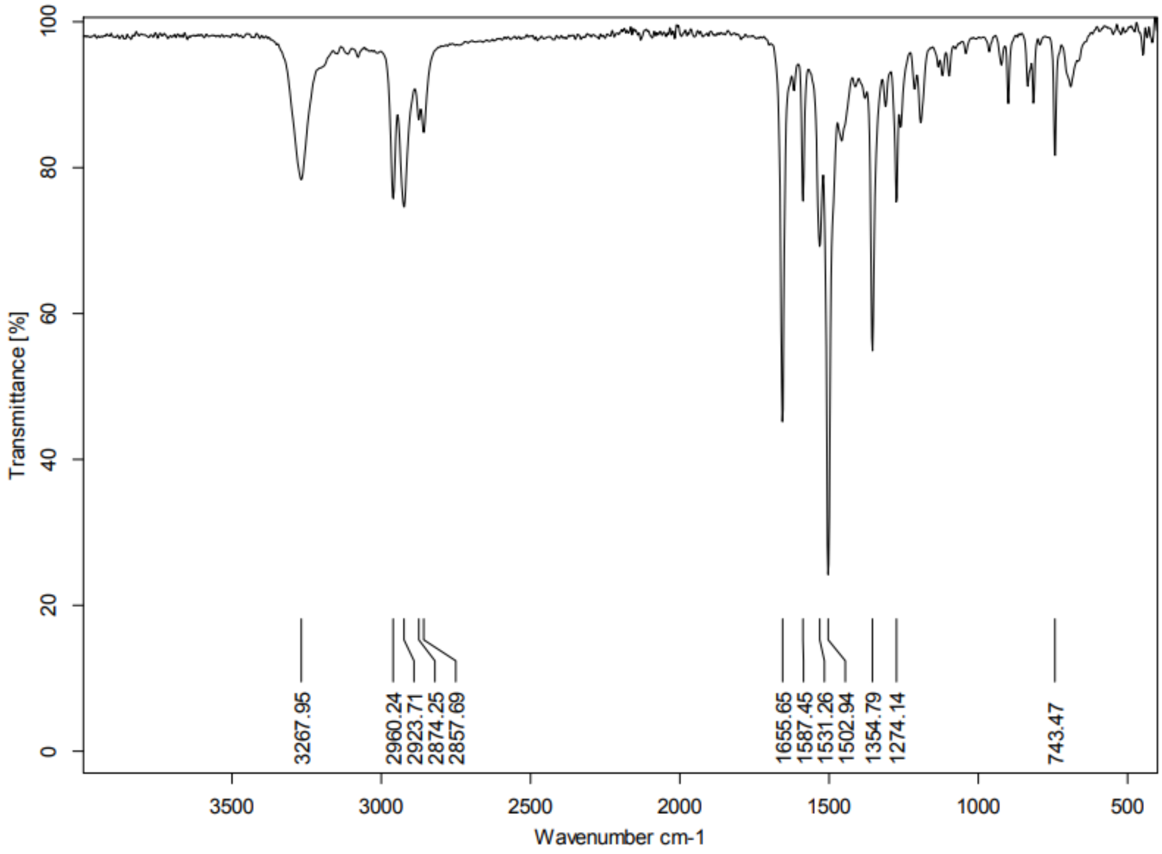

Figure S.4 – FT-IR spectrum of **1**

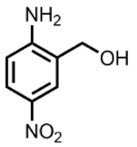

2-amino-5-nitrobenzyl alcohol, **2**. 2-amino-5-nitrobenzoic acid (2.51 g, 13.8 mmol) was dissolved in dry THF (80 mL) at 0 °C. BH_3·THF was added (1 M in THF, 30 mL, 30 mmol) was added dropwise and the reaction warmed to room temperature overnight. The reaction was quenched with water (30 mL), then extracted with EtOAc (3 x 100 mL). The combined organic layers were washed with sat $NaHCO_3$ (100 mL) and brine (100 mL). The organic layer was dried with $MgSO_4$, and the solvent removed under reduced pressure to yield the desired product as a yellow solid (1.90 g, 11.3 mmol, 82%).

m.p. – 142 – 143 °C

ν_{max} (film) cm^{-1}: 3378 (N-H), 3342 (O-H), 3199 (N-H), 2932 (C-H), 2885 (C-H), 1645 (C=C), 1607 (C=C), 1587 (C=C), 1493 (C=C), 1306, 1101, 1016

^1H NMR (400 MHz, MeOD) δ_H ppm: δ 8.07 (d, J = 2.7 Hz, 1H), 7.95 (dd, J = 8.9, 2.7 Hz, 1H), 6.69 (d, J = 9.0 Hz, 1H), 4.85 (s, 2H), 4.57 (s, 2H), 3.35 (s, 1H).

$^{13}C\{^1H\}$ NMR (101 MHz, MeOD) δ_C ppm: 154.54, 138.42, 126.15, 125.57, 124.99, 114.58, 62.39.

HRMS: calc for $C_7H_9N_2O_3^+$ $[M+H]^+$: 169.0613, found: 169.0599

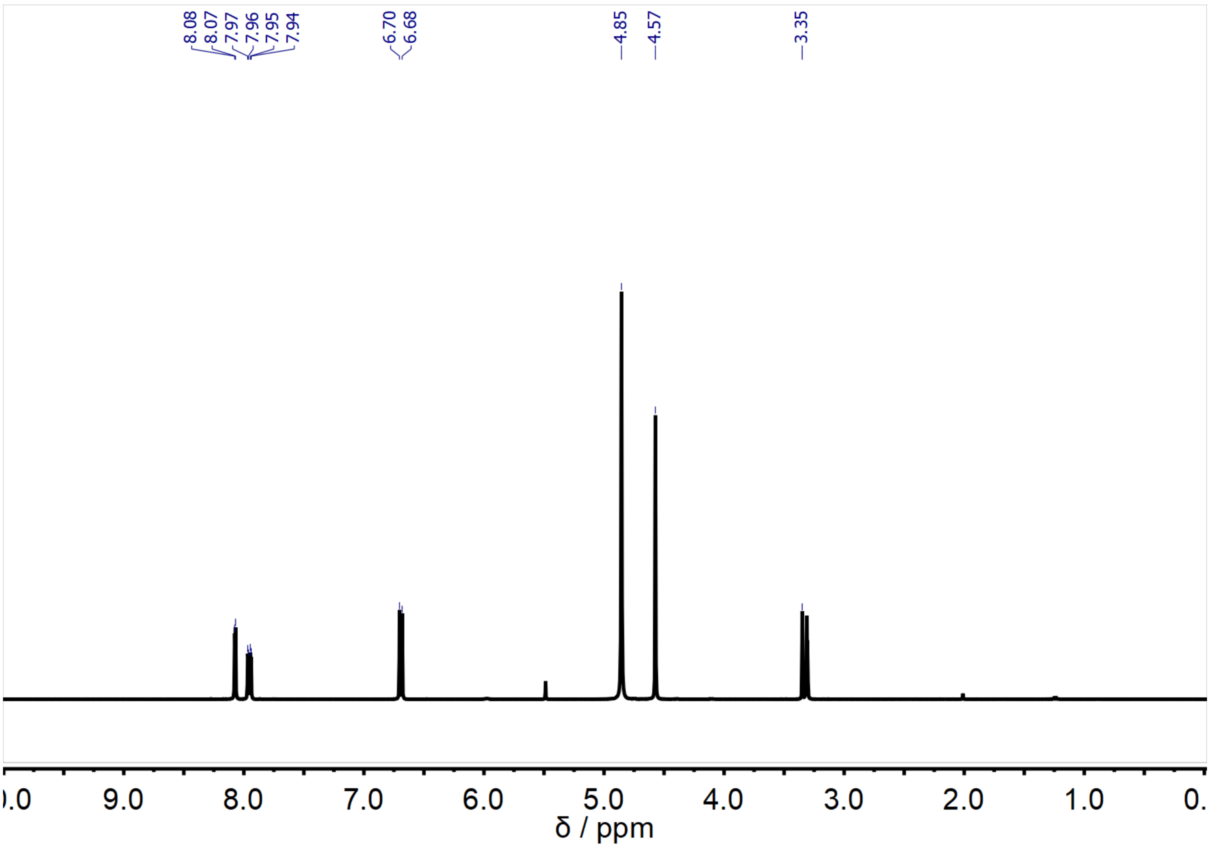

Figure S.5 – ^1H NMR spectrum of **2** in MeOD (δ 0 – 10 ppm).

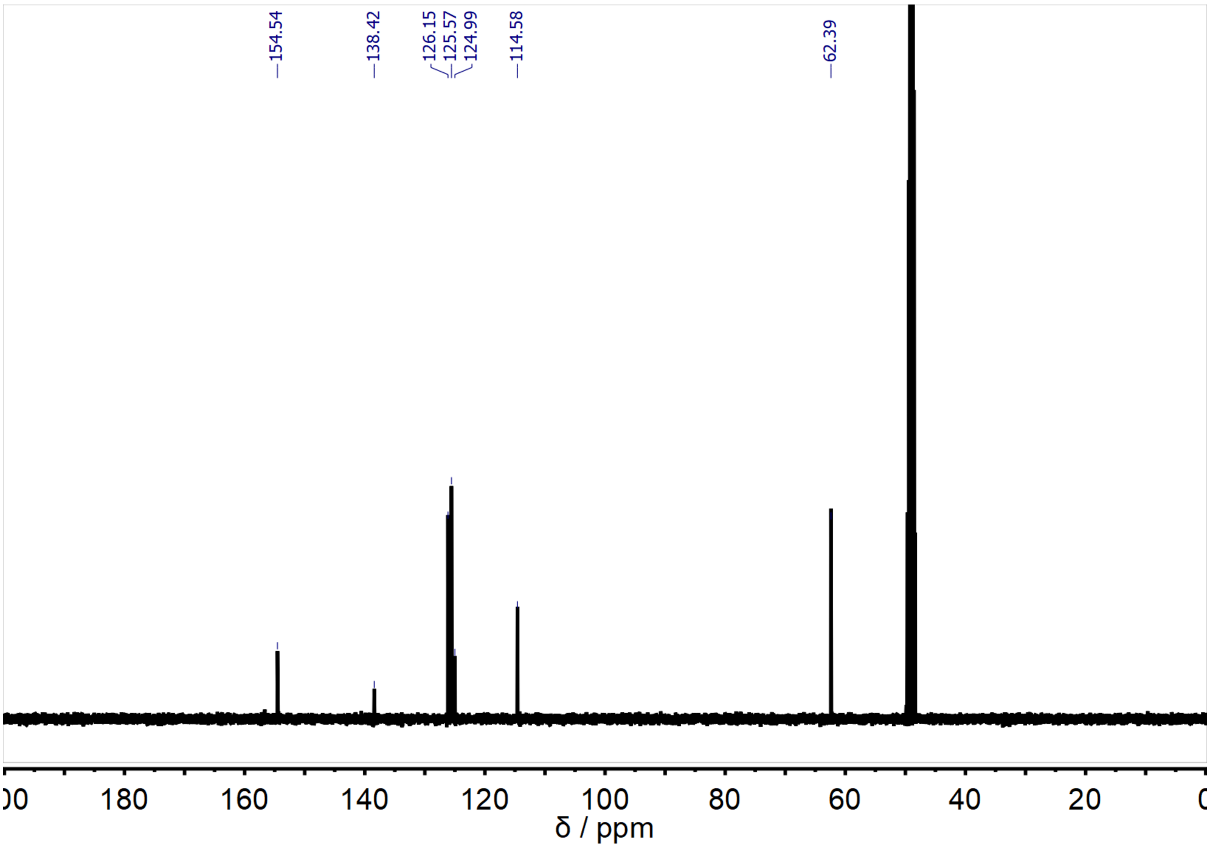

Figure S.6 – ^{13}C NMR spectrum of **2** in MeOD (δ 0 – 200 ppm).

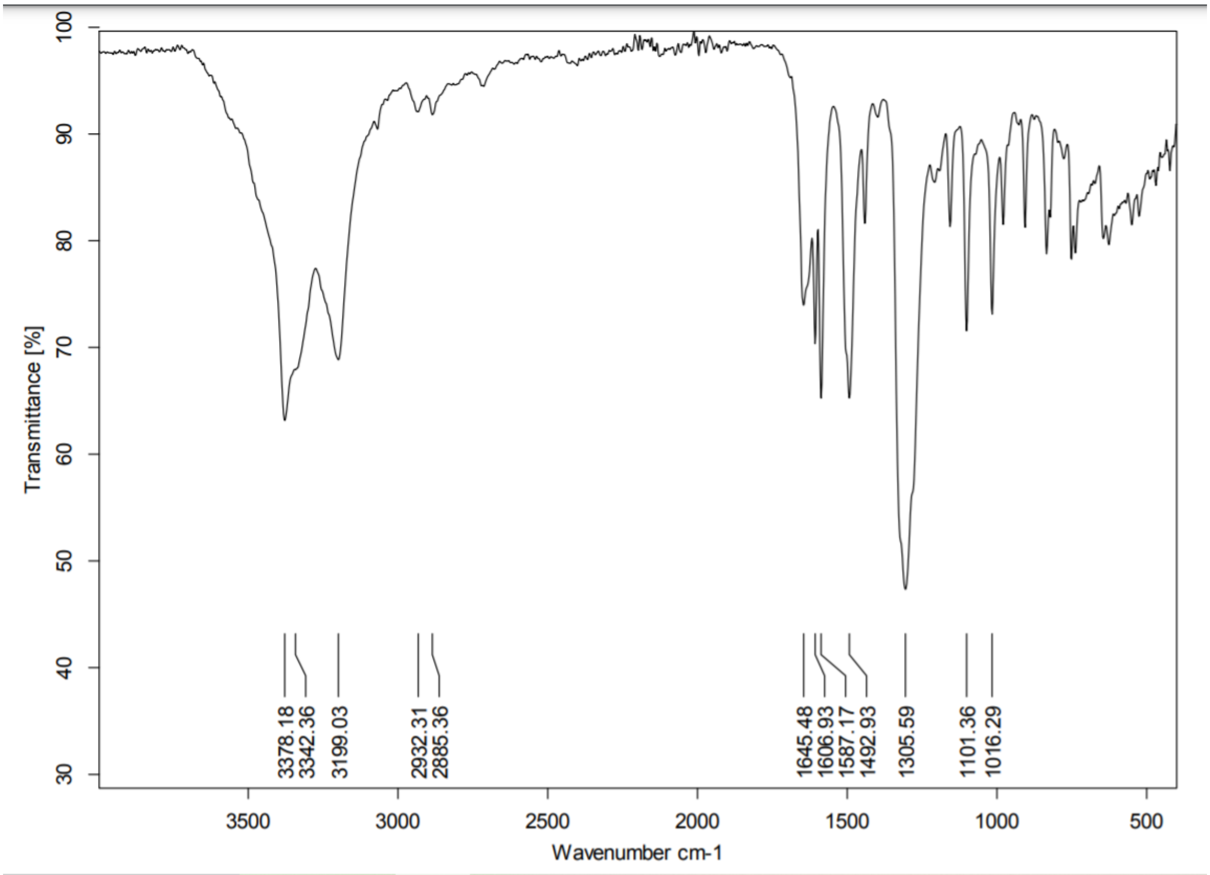

Figure S.7 – FT-IR spectrum of **2**

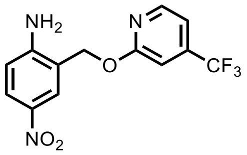

4-trifluoromethyl-2-((pyridin-2-yloxy)methyl)aniline, **3**. 2-amino-5-nitrobenzyl alcohol (57.1 mg, 0.340 mmol) and sodium hydride (60% in mineral oil, 28.6 mg, 0.715 mmol) were dissolved in DMF (3.0 mL) under a nitrogen atmosphere and stirred for 60 minutes. 4-trifluoromethyl-2-fluoropyridine was added (50 µL, 0.410 mmol) was added dropwise and the reaction stirred for 24 hours. The reaction was quenched with saturated NH_4Cl (5 mL), then the mixture was diluted with EtOAc (50 mL) and washed with $NaHCO_3$ (25 mL), water (2 x 25 mL) and LiCl (5% solution, 25 mL). The organic layer was dried with $MgSO_4$, and the solvent removed under reduced pressure. The crude residue was purified by flash chromatography (EtOAc/PE on SiO_2) to leave the desired product as a yellow solid (28.1 mg, 0.0897 mmol, 26%)

ν_{max} (film) cm^{-1} : 3481 (N-H), 3379 (N-H), 3229 (C-H), 2919 (C-H), 1632 (C-C), 1610 (C-C), 1586 (C-C), 1490, 1417, 1329, 1316, 1284, 1173, 1138, 1101, 1003

^1H NMR (400 MHz, $CDCl_3$) δ_H ppm: 8.34 (d, J = 5.3 Hz, 1H), 8.27 (d, J = 2.6 Hz, 1H), 8.07 (dd, J = 8.9, 2.6 Hz, 1H), 7.14 (dd, J = 5.3, 1.4 Hz, 1H), 7.05 – 7.01 (m, 1H), 6.67 (d, J = 8.9 Hz, 1H), 5.44 (s, 2H), 5.02 (s, 2H).

$^{13}C\{^1H\}$ NMR (101 MHz, $CDCl_3$) δ_C ppm: 163.5, 152.2, 148.3, 138.9, 128.2, 126.5, 125.1, 124.0, 119.8, 114.9, 113.3 (q, J = 3.1 Hz), 108.3 (q, J = 3.9 Hz), 65.7.

HRMS: calc for $C_{13}H_{11}N_3O_3F_3^+$ $[M+H]^+$: 314.0747, found: 314.0746

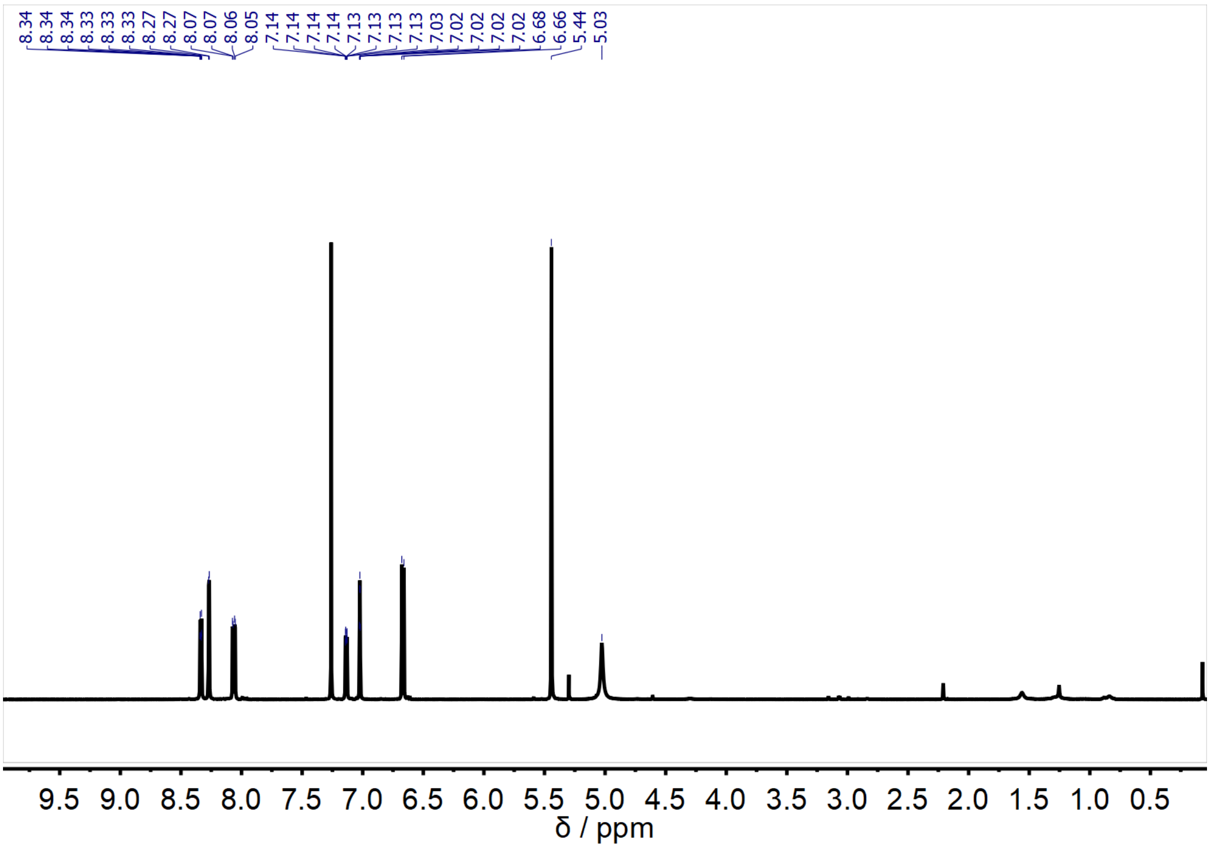

Figure S.8 – ^1H NMR spectrum of **3** in $CDCl_3$ (δ 0 – 10ppm).

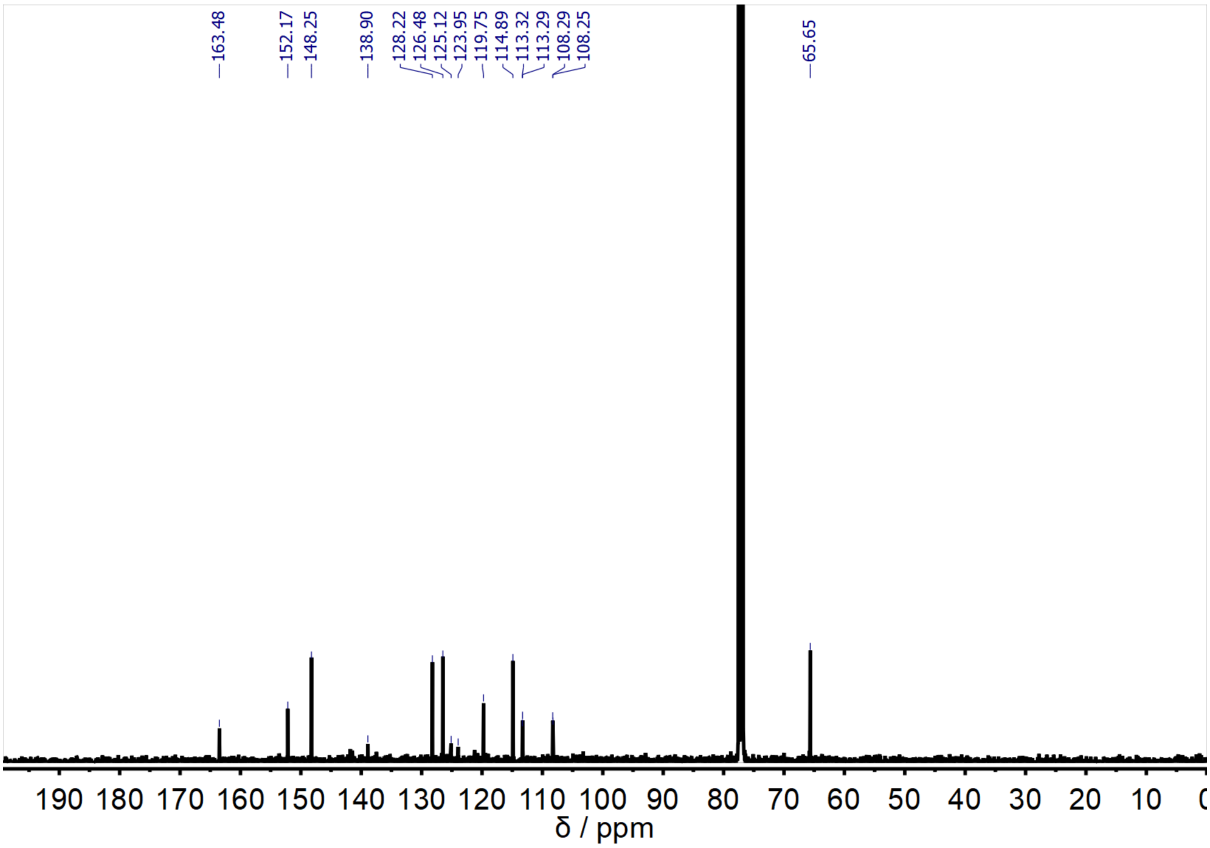

Figure S.9 – ^{13}C NMR spectrum of **3** in $CDCl_3$ (δ 0 – 200 ppm).

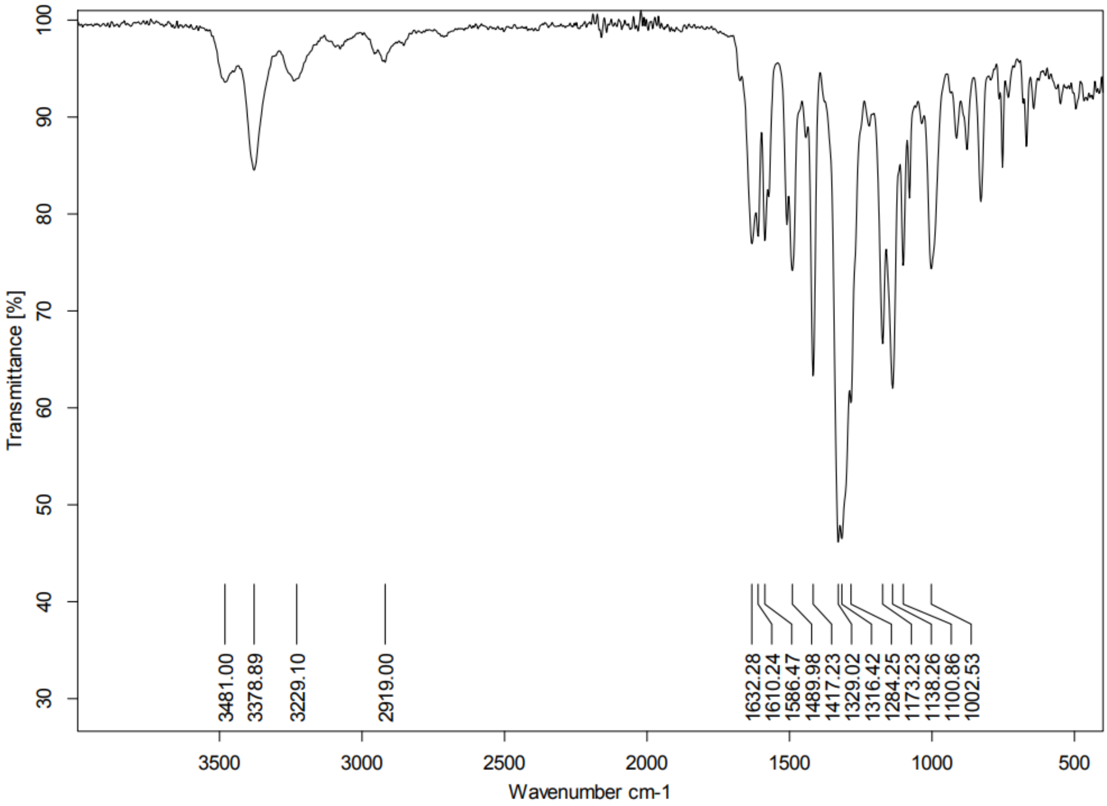

Figure S.10 – FT-IR spectrum of **3**

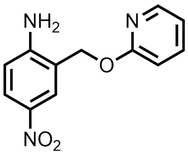

4-nitro-2-((pyridin-2-yloxy)methyl)aniline, **4**. 2-amino-5-nitrobenzyl alcohol (83.3 mg, 0.495 mmol) and sodium hydride (60% in mineral oil, 41.8 mg, 1.045 mmol) were dissolved in DMF (4.0 mL) under a nitrogen atmosphere and stirred for 60 minutes. 2-fluoropyridine was added (50 µL, 0.58 mmol) was added dropwise and the reaction stirred for 24 hours. The reaction mixture was diluted with EtOAc (50 mL) and washed with $NaHCO_3$ (25 mL), water (2 x 25 mL) and LiCl (5% solution, 25 mL). The organic layer was dried with $MgSO_4$, and the solvent removed under reduced pressure. The crude residue was purified by flash chromatography (EtOAc/PE on SiO_2) to leave the desired product as a yellow solid (48.7 mg, 0.199 mmol, 40%)

ν_{max} (film) cm^{-1} : 3478 (N-H), 3376 (N-H), 3324 (N-H), 2927 (C-H), 1631 (C=C), 1608 (C=C), 1585 (C=C), 1571 (C=C), 1507, 1488, 1472, 1430, 1302, 1283, 1251, 1154, 1100, 1043, 989, 909, 827, 779, 752

^{1}H NMR (400 MHz, $CDCl_3$) δ_H ppm: 8.26 (d, J = 2.6 Hz, 1H), 8.17 (dd, J = 5.1, 1.9 Hz, 1H), 8.05 (dd, J = 8.9, 2.6 Hz, 1H), 7.61 (ddd, J = 8.6, 7.1, 2.0 Hz, 1H), 6.93 (t, J = 6.3 Hz, 1H), 6.79 (d, J = 8.3 Hz, 1H), 6.64 (d, J = 8.9 Hz, 1H), 5.39 (s, 2H), 5.23 (s, 2H).

$^{13}C\{^{1}H\}$ NMR (101 MHz, $CDCl_3$) δ_C ppm: 163.15, 152.47, 146.69, 139.34, 138.73, 128.23, 126.33, 120.47, 117.70, 114.76, 111.63, 110.13, 65.02.

HRMS: calc for $C_{11}H_{11}N_3O_3^+$ $[M+H]^+$: 246.0868, found: 246.0873

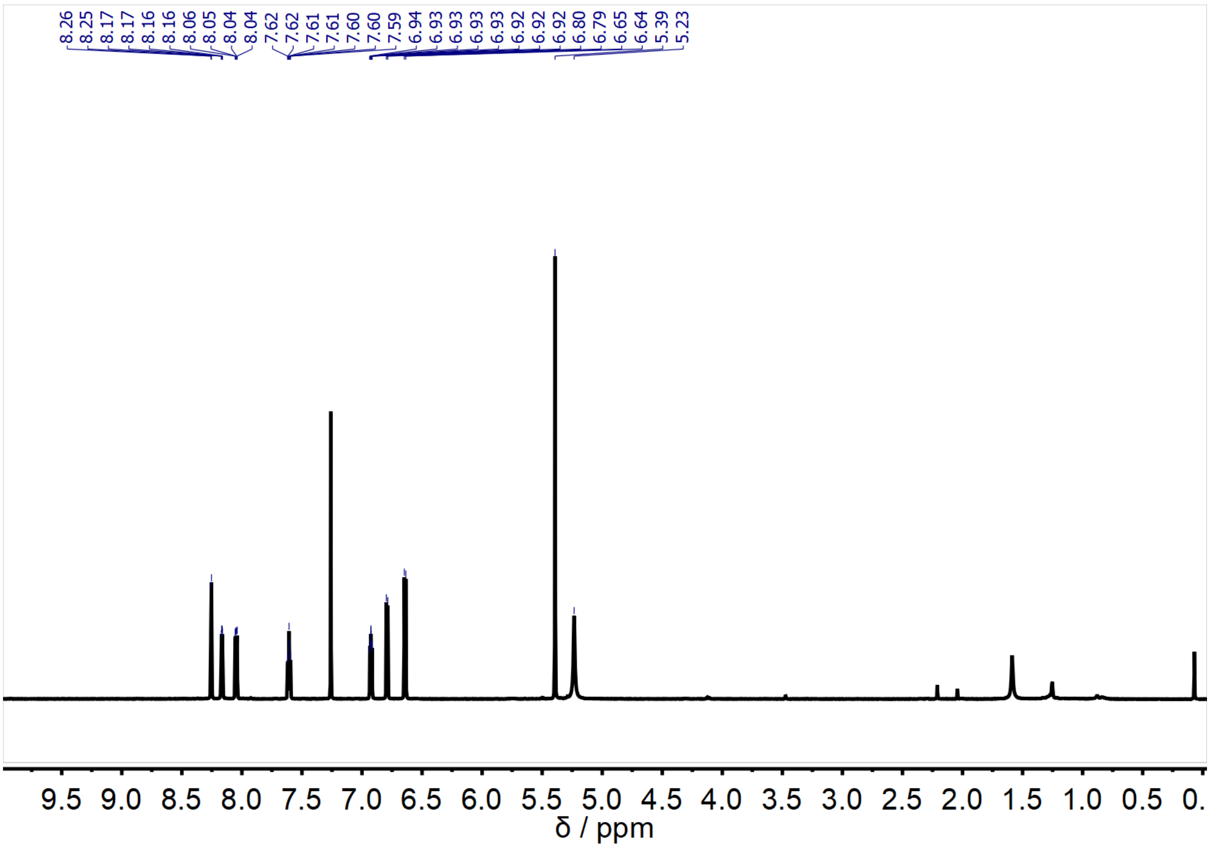

Figure S.12 – ^{1}H NMR spectrum of **4** in $CDCl_3$ (δ 0 – 10 ppm).

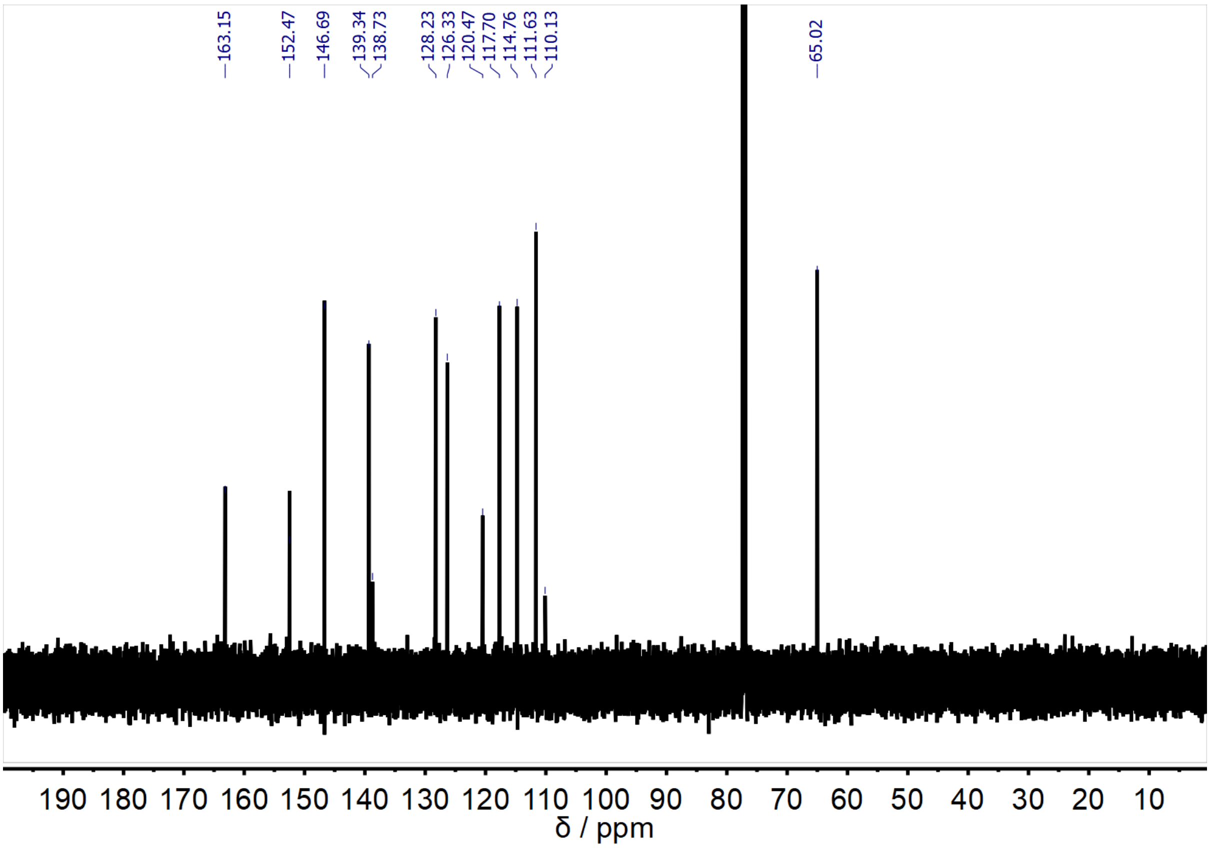

Figure S.13 – ^{13}C NMR spectrum of **4** in $CDCl_3$ (δ 0 – 200 ppm).

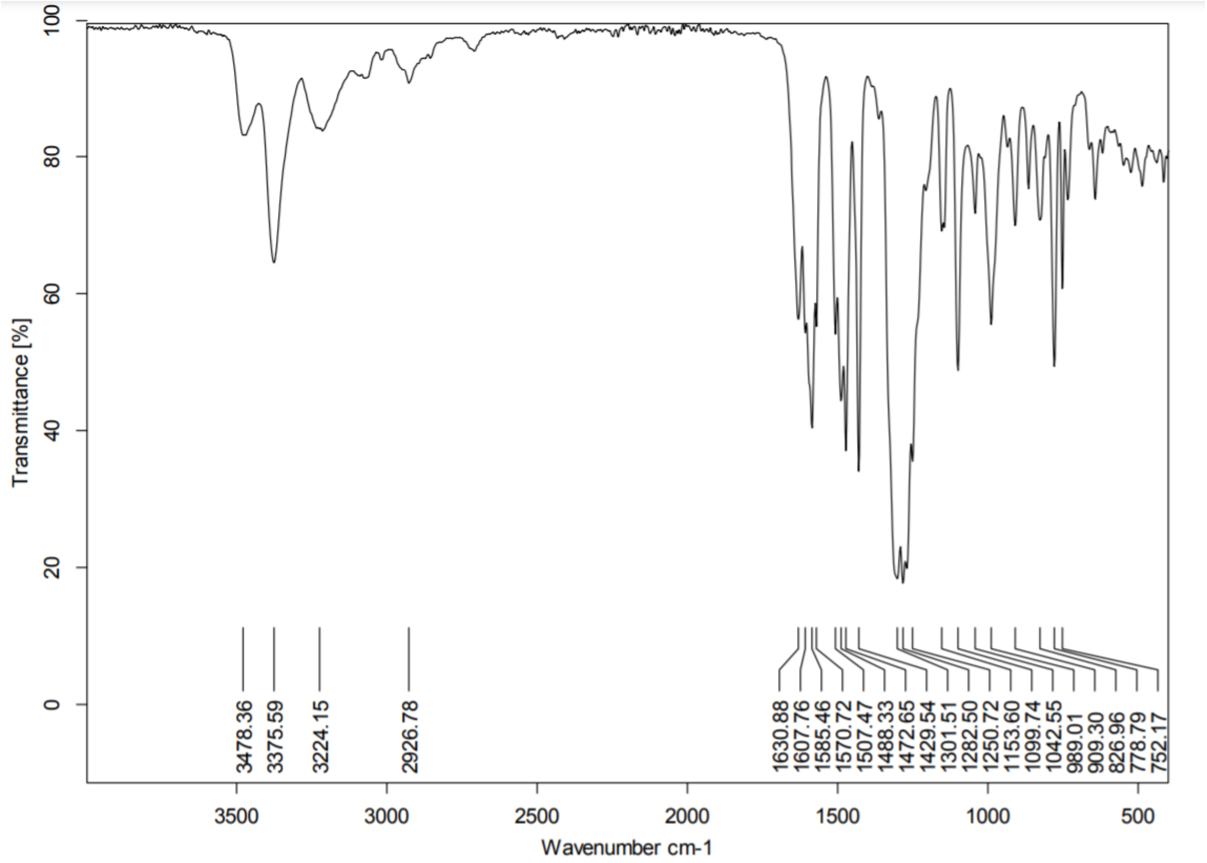

Figure S.14 – FT-IR spectrum of **4**

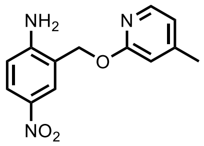

4-methyl-2-((pyridin-2-yloxy)methyl)aniline, **5**. 2-amino-5-nitrobenzyl alcohol (0.341 g, 2.03 mmol), sodium hydride (60% in mineral oil, 0.166 g, 4.15 mmol), and 2-fluoro-4-methylpyridine were dissolved in DMF (5.0 mL) under a nitrogen atmosphere and stirred for 24 hours. The reaction was quenched with saturated NH_4Cl solution (5 mL), diluted with EtOAc (50 mL), and washed with $NaHCO_3$ (25 mL), water (2 x 25 mL) and LiCl (5% solution, 25 mL). The organic layer was dried with $MgSO_4$, and the solvent removed under reduced pressure. The crude residue was purified by flash chromatography (EtOAc/PE on SiO_2) to leave the desired product as a yellow solid (60.1 mg, 0.232 mmol, 11%)

ν_{max} (film) cm^{-1} : 3444 (N-H), 3383 (N-H), 3321 (N-H), 3182 (C-H), 2920 (C-H), 1647 (C=C), 1609 (C=C), 1596 (C=C), 1511 , 1585, 1454, 1298, 1098, 818

^1H NMR (400 MHz, $CDCl_3$) δ_H ppm: 8.24 (d, J = 2.6 Hz, 1H), 8.04 (dd, J = 8.9, 2.6 Hz, 1H), 8.01 (d, J = 5.2 Hz, 1H), 6.75 (dd, J = 5.3, 1.4 Hz, 1H), 6.63 (d, J = 8.9 Hz, 1H), 6.60 (s, 1H), 5.37 (s, 2H), 5.29 (s, 2H), 2.30 (s, 3H).

$^{13}C\{^1H\}$ NMR (101 MHz, $CDCl_3$) δ_C ppm: 163.5, 152.5, 150.9, 146.1, 138.7, 128.3, 126.3, 120.6, 119.3, 114.7, 114.3, 111.7, 64.9, 21.1.

HRMS: calc for $C_{13}H_{14}N_3O_3^+$ $[M+H]^+$: 260.1035, found: 260.1021

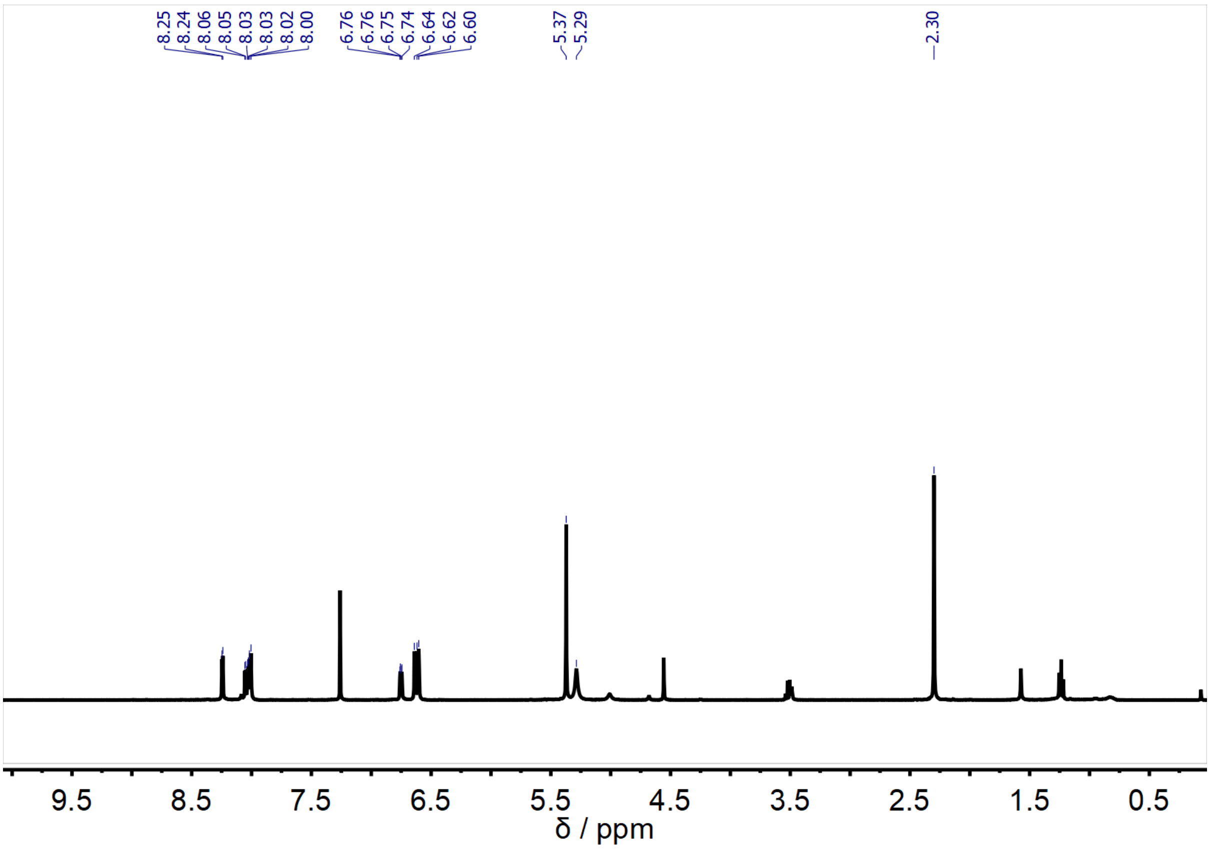

Figure S.15 – ^1H NMR spectrum of **5** in $CDCl_3$ (δ 0 – 10 ppm).

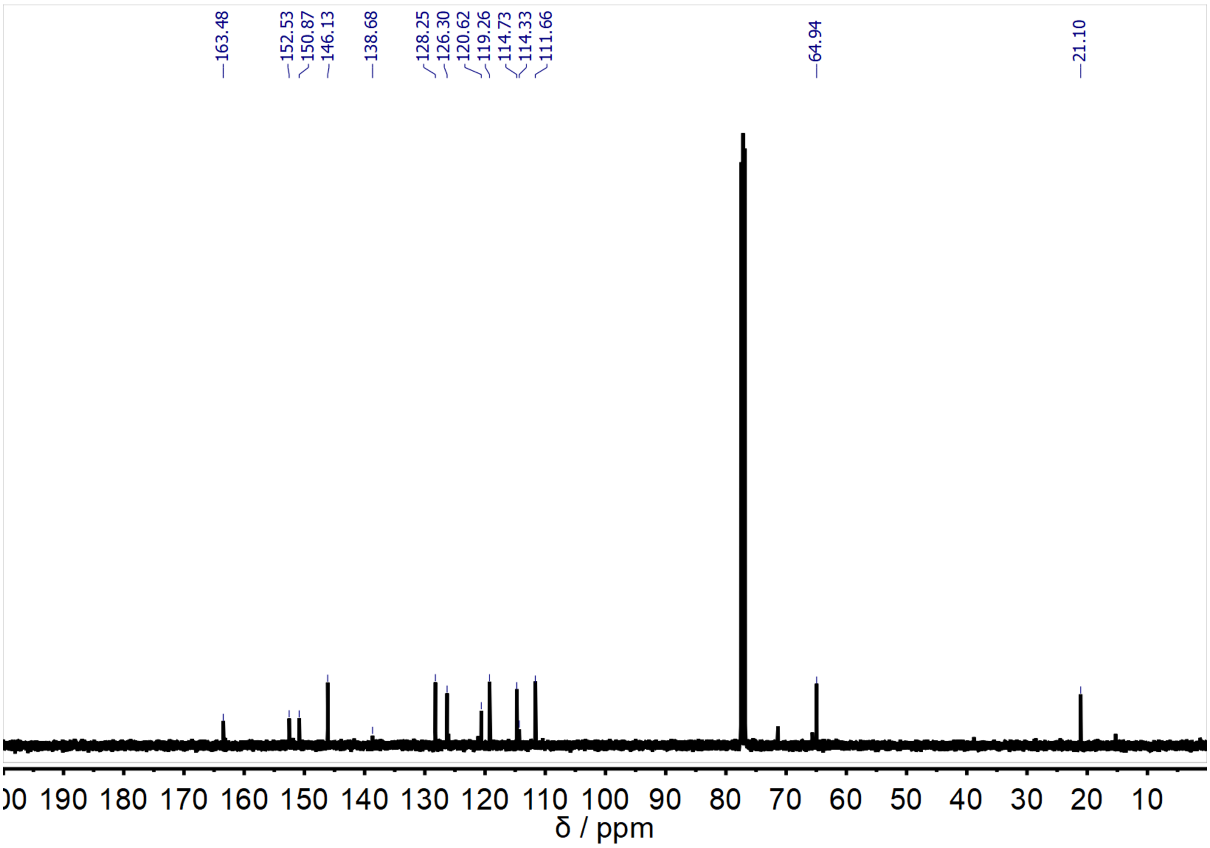

Figure S.16 – ^{13}C NMR spectrum of **5** in $CDCl_3$ (δ 0 – 200 ppm).

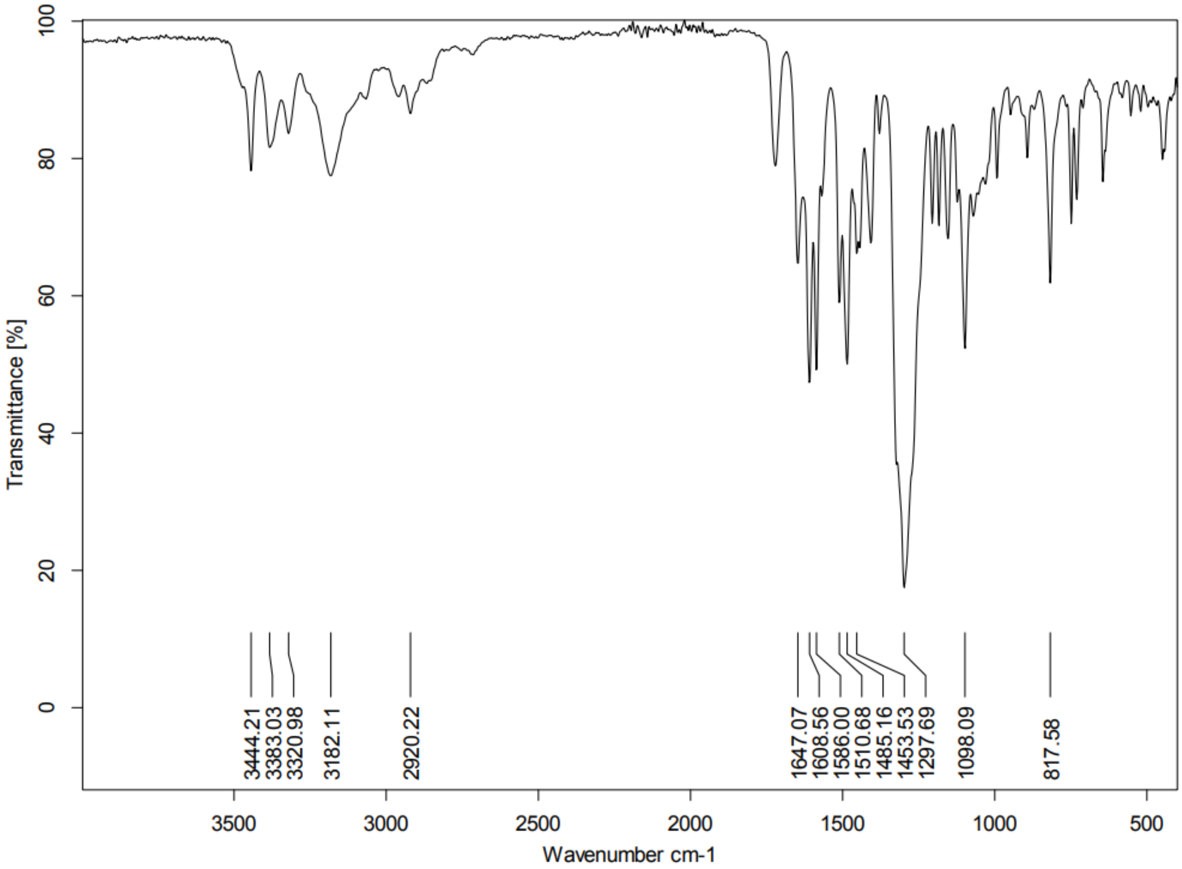

Figure S.17 – FT-IR spectrum of **5**

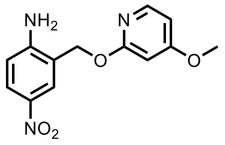

4-methoxy-2-((pyridin-2-yloxy)methyl)aniline, **6**. 2-amino-5-nitrobenzyl alcohol (63.0 mg, 0.375 mmol) and sodium hydride (60% in mineral oil, 35.2 mg, 0.880 mmol) were dissolved in DMF (3.0 mL) under a nitrogen atmosphere and stirred for 4 hours. 4-methoxy-2-fluoropyridine was added (200 µL, 0.802 mmol) was added dropwise and the reaction stirred for 3 days. The reaction mixture was quenched with saturated NH_4Cl (5 mL), diluted with EtOAc (50 mL) and washed with $NaHCO_3$ (25 mL), water (2 x 25 mL) and LiCl (5% solution, 25 mL). The organic layer was dried with $MgSO_4$, and the solvent removed under reduced pressure. The crude residue was purified by flash chromatography (EtOAc/PE on SiO_2) to leave the desired product as a yellow solid (5.5 mg, 0.0199 mmol, 5%)

ν_{max} (film) cm^{-1}: 3468 (N-H), 3379 (N-H), 3222 (C-H), 2939 (C-H), 1603 (C=C), 1586 (C=C), 1573 (C=C), 1508, 1485, 1439, 1312, 1200, 1162, 1100, 1038

^{1}H NMR (400 MHz, $CDCl_3$) δ_H ppm: 8.24 (d, J = 2.7 Hz, 1H), 8.05 (dd, J = 8.9, 2.7 Hz, 1H), 7.96 (d, J = 5.9 Hz, 1H), 6.63 (d, J = 8.8 Hz, 1H), 6.52 (dd, J = 6.0, 2.2 Hz, 1H), 6.25 (d, J = 2.2 Hz, 1H), 5.38 (s, 2H), 5.34 (s, 2H), 3.81 (s, 3H).

$^{13}C\{^{1}H\}$ NMR (101 MHz, $CDCl_3$) δ_C ppm: 168.4, 165.2, 152.5, 147.1, 138.7, 128.3, 126.4, 120.5, 114.8, 107.2, 94.7, 65.1, 55.5.

HRMS: calc for $C_{13}H_{14}N_3O_4^+$ $[M+H]^+$: 276.0984, found: 276.0985

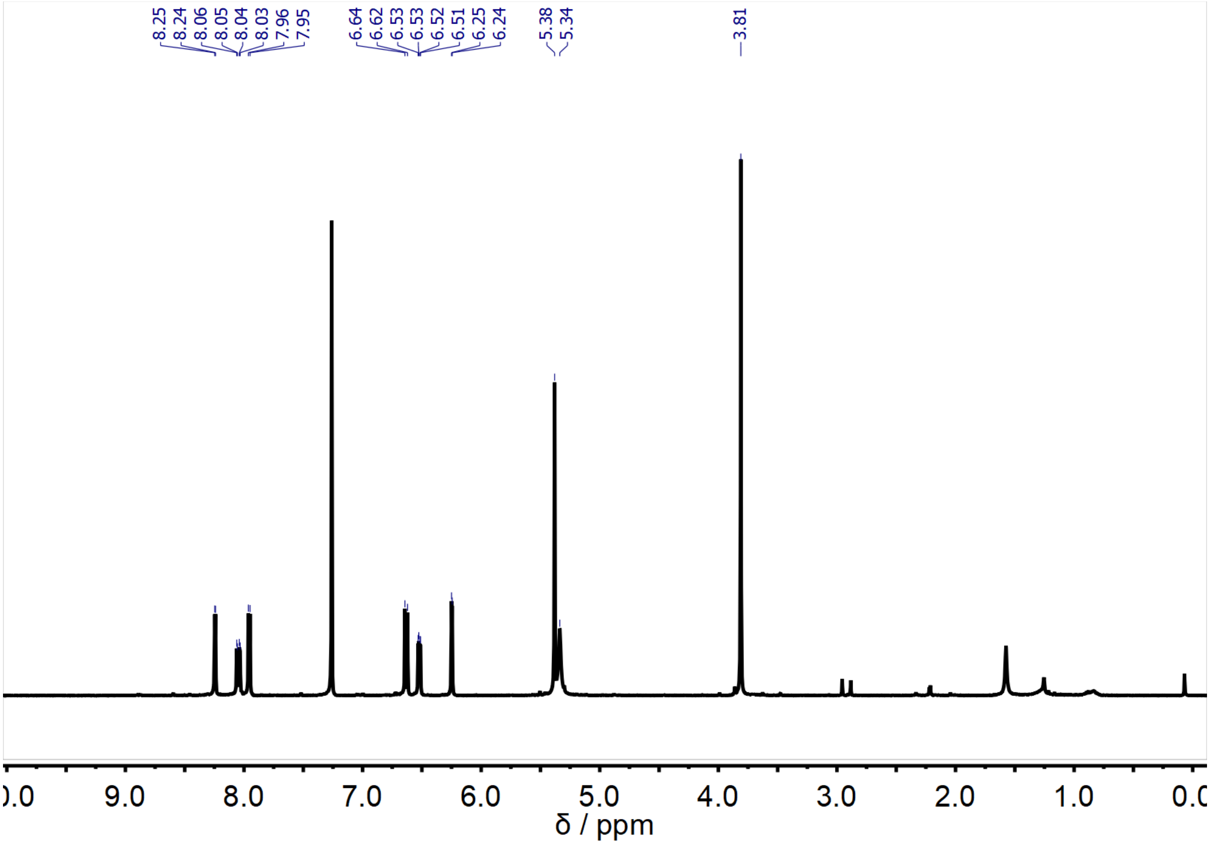

Figure S.18 – ^{1}H NMR spectrum of **6** in $CDCl_3$ (δ 0 – 10 ppm).

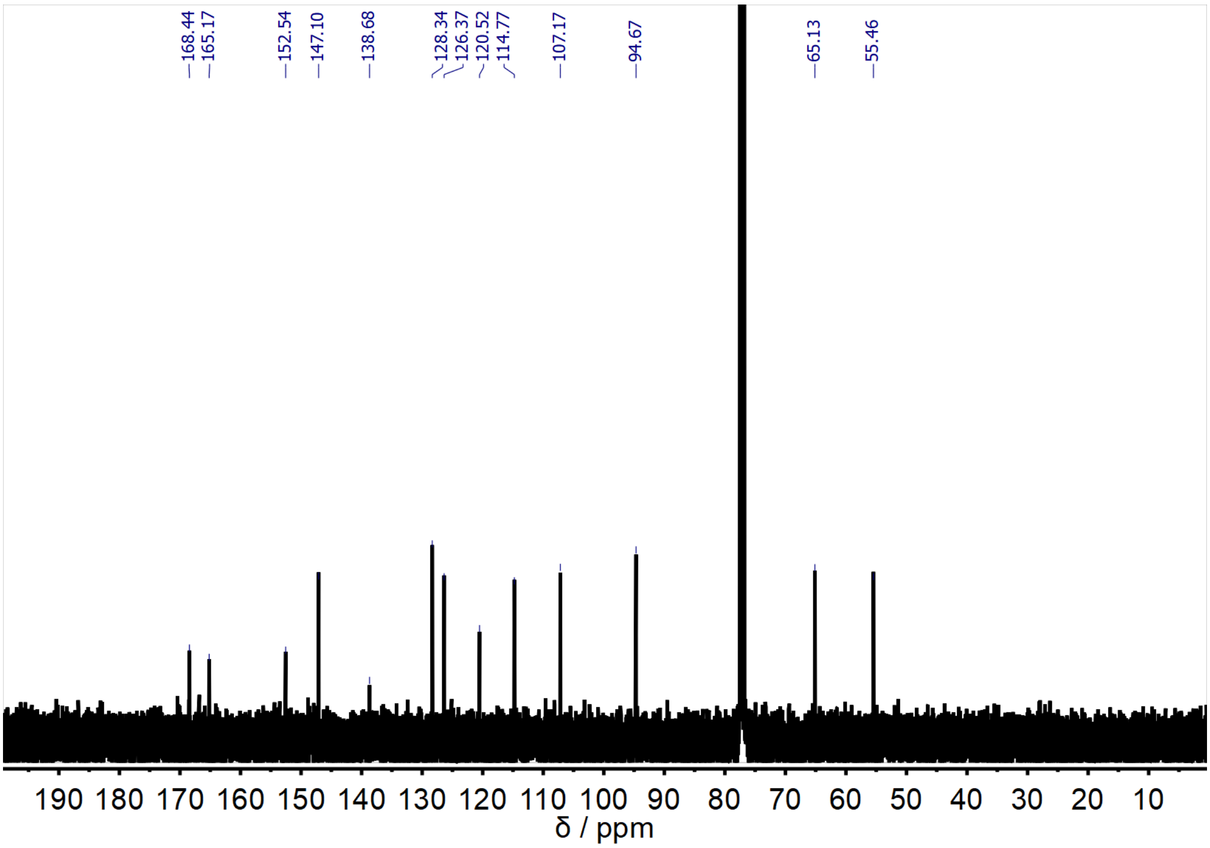

Figure S.19 – ^{13}C NMR spectrum of **6** in $CDCl_3$ (δ 0 – 200 ppm).

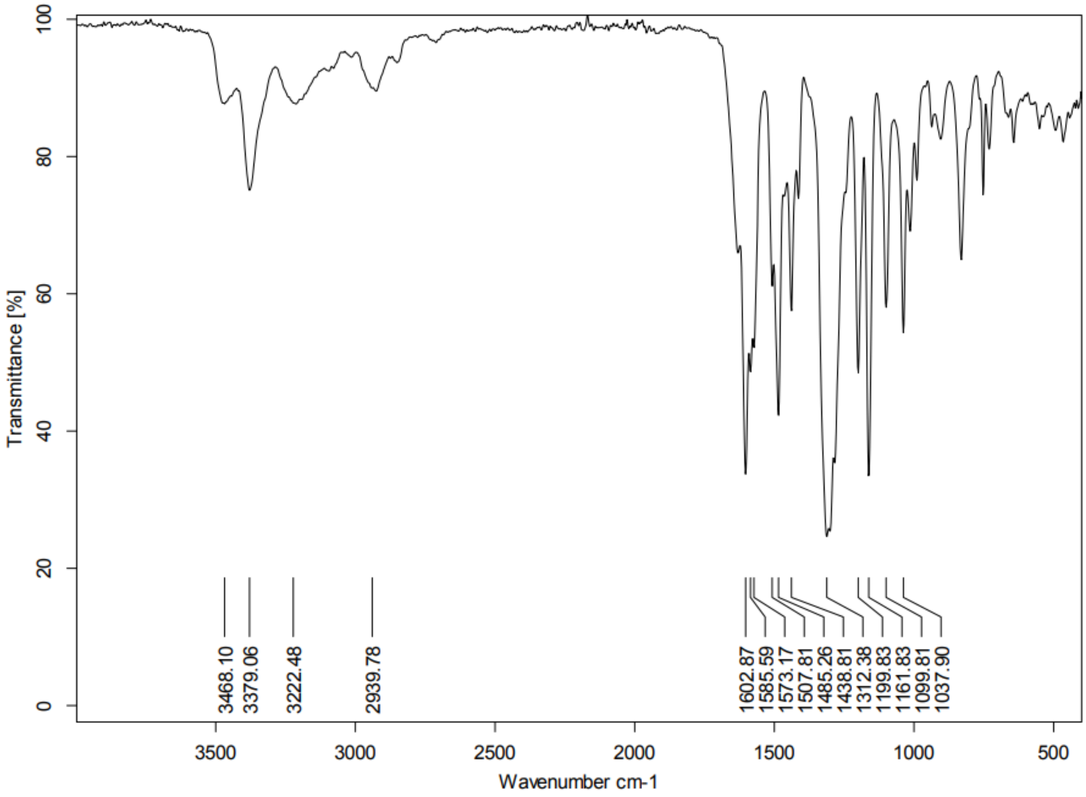

Figure S.20 – FT-IR spectrum of **6**

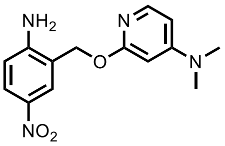

4-dimethylamino-2-((pyridin-2-yloxy)methyl)aniline, **7**. 2-amino-5-nitrobenzyl alcohol (0.244 g, 1.45 mmol), sodium hydride (60% in mineral oil, 0.240 g, 3.00 mmol) and 2-fluoro-4-(dimethylamino)pyridine (0.363 g, 2.74 mmol) were dissolved in DMF (6.0 mL) under a nitrogen atmosphere and stirred for 5 days. The reaction was quenched with water (30 mL), diluted with EtOAc (50 mL), and washed with $NaHCO_3$ (2 x 50 mL), LiCl (5% solution, 50 mL) and brine (50 mL). The organic layer was dried with $MgSO_4$, and the solvent removed under reduced pressure. The crude residue was purified by flash chromatography (EtOAc/PE on SiO_2, then DCM/MeOH on SiO_2 and then DCM/EtOAc on Al_2O_3) to leave the desired product as a yellow solid (45.1 mg, 0.156 mmol, 11%)

ν_{max} (film) cm^{-1}: 3365 (N-H), 3208 (N-H), 2947 (C-H), 2855 (C-H), 1609 (C=C), 1587 (C=C), 1510, 1492, 1442, 1317, 1284, 1257, 1157, 1029, 732

^{1}H NMR (700 MHz, $CDCl_3$) δ_H ppm: 8.23 (d, J = 2.6 Hz, 1H), 8.04 (dd, J = 8.9, 2.6 Hz, 1H), 7.82 (d, J = 6.1 Hz, 1H), 6.61 (d, J = 8.9 Hz, 1H), 6.27 (dd, J = 6.2, 2.4 Hz, 1H), 5.91 (d, J = 2.3 Hz, 1H), 5.55 (s, 2H), 5.36 (s, 2H), 2.97 (s, 6H).

$^{13}C\{^{1}H\}$ NMR (101 MHz, $CDCl_3$) δ_C ppm: 164.8, 157.3, 152.8, 146.3, 138.5, 128.4, 126.3, 120.9, 114.7, 103.4, 91.4, 64.9, 39.5.

HRMS: calc for $C_{13}H_{17}N_4O_3^+$ $[M+H]^+$: 289.1301, found: 289.1300

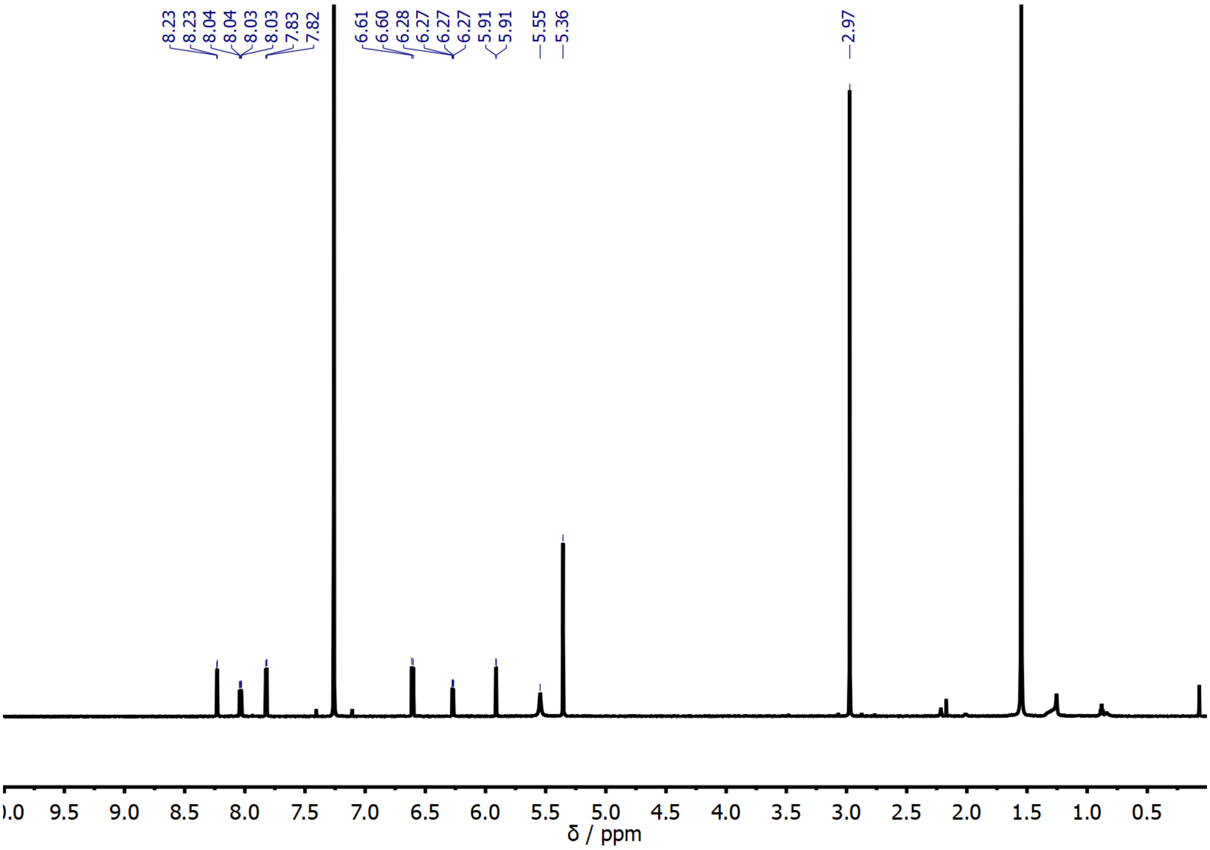

Figure S.21 – ^{1}H NMR spectrum of **7** in $CDCl_3$ (δ 0 – 10 ppm).

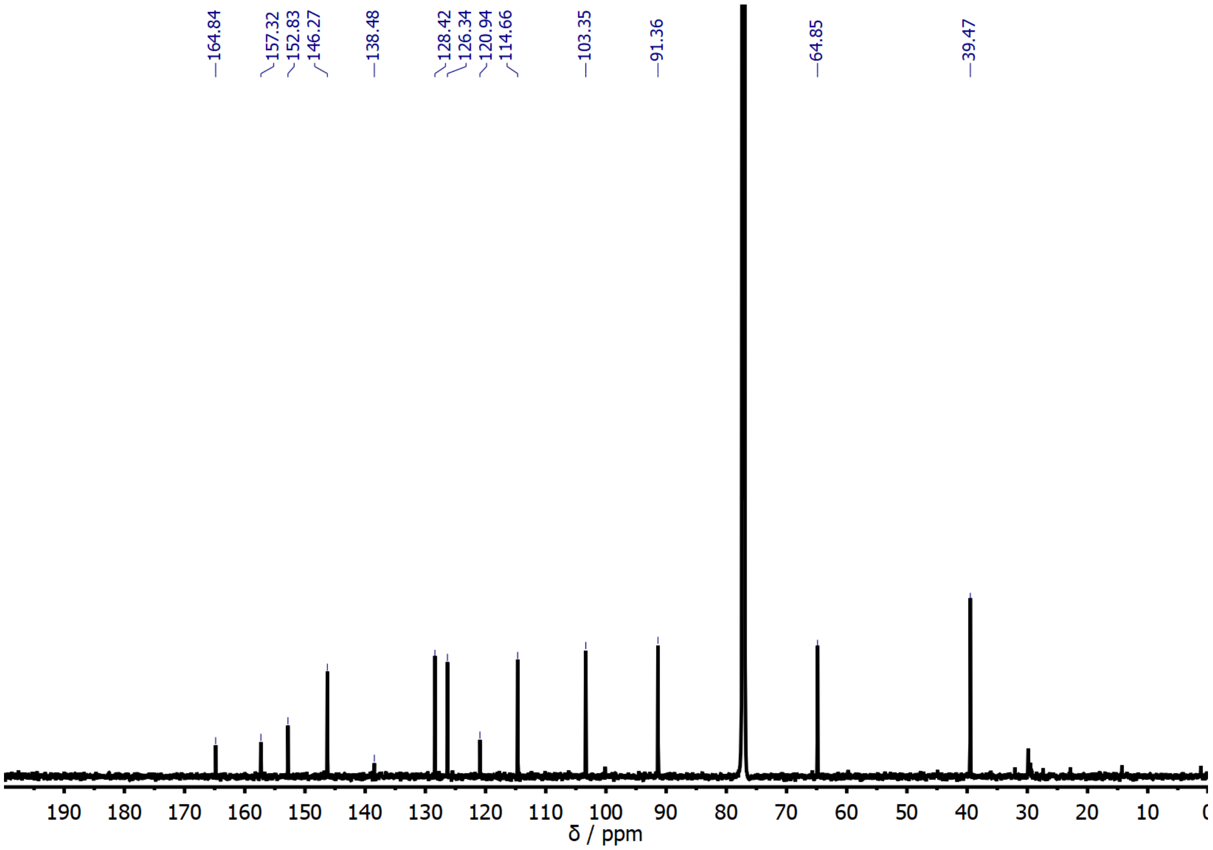

Figure S.22 – ^{13}C NMR spectrum of **7** in $CDCl_3$ (δ 0 – 200 ppm).

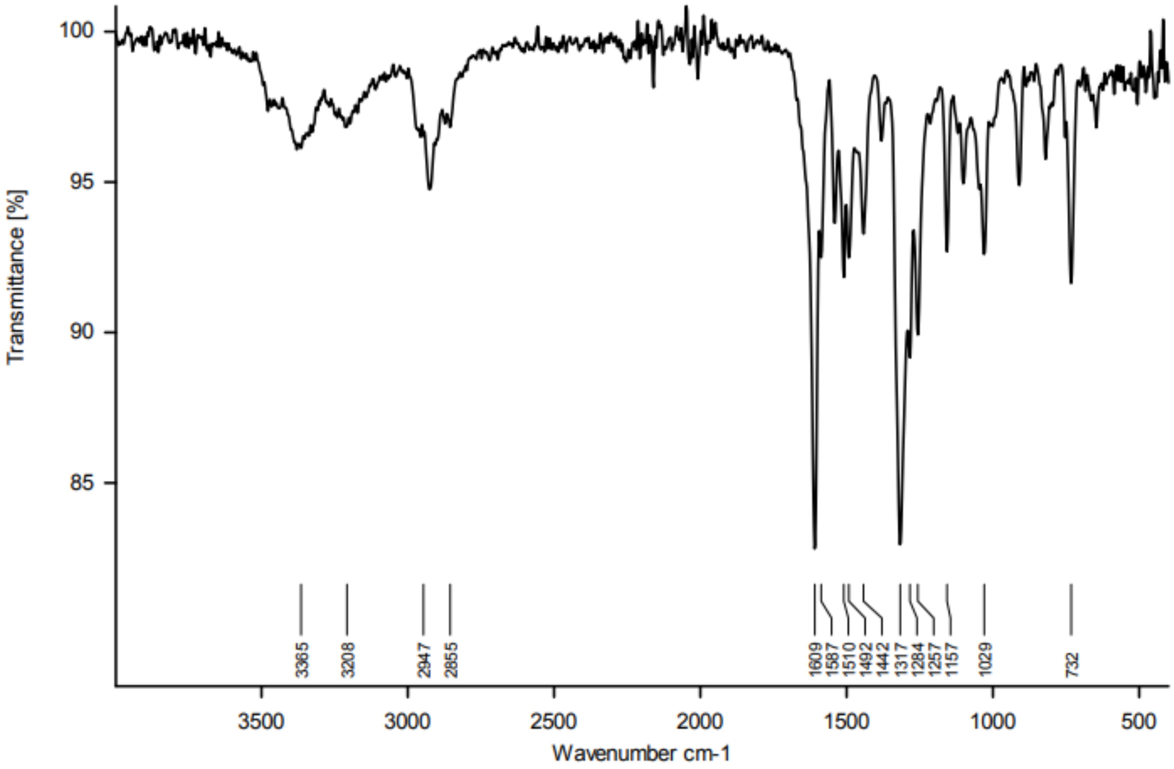

Figure S.23 – FT-IR spectrum of **7**

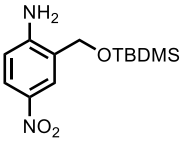

2-(((*tert*-butyldimethylsilyl)oxy)methyl)-4-nitroaniline, **8**. 2-amino-5-nitrobenzyl alcohol (0.891 g, 5.30 mmol) and imidazole (1.15 g, 17.0 mmol) dissolved in dry DMF (6 mL) at 0 ^{o}C. *Tert*-butyldimethylsilyl chloride (1.19 g, 7.89 mmol), the cooling bath was removed and stirred for 4 hours. Reaction mixture quenched with 1% KH_2PO_4 (20 mL), then extracted with EtOAc (3 x 25 mL). The combined organic layers washed with brine (25 mL) and then dried with $MgSO_4$. The solvent was removed in vacuo and the crude purified by flash chromatography (PE/EtOAc on SiO_2) to yield the desired product as a yellow solid (1.49 g, quant.).

m.p. – 72 – 78 ^{o}C (acetone)

ν_{max} (film) cm^{-1} : 3477 (N-H), 3375 (N-H), 2956 (C-H), 2929 (C-H), 2856 (C-H), 1623 (C=C), 1586 (C=C), 1507 (C=C), 1490 (C=C), 1305, 1283, 1258, 1101, 1065, 831, 778, 751.

^{1}H NMR (400 MHz, $CDCl_3$) δ_H ppm: 8.05 (dd, J = 8.8, 2.6 Hz, 1H), 7.99 (d, J = 2.6 Hz, 1H), 6.64 (d, J = 8.8 Hz, 1H), 5.03 (s, 2H), 4.74 (s, 2H), 0.92 (s, 9H), 0.12 (s, 6H).

^{13}C{^{1}H} NMR (101MHz, $CDCl_3$) δ_c ppm: 152.5, 138.3, 125.6, 124.7, 123.7, 114.2, 64.5, 25.8, 18.2, -5.3.

HRMS: calc for $C_{13}H_{21}N_2O_3Si^+$ [M-H]$^-$: 281.1321, found: 281.1331

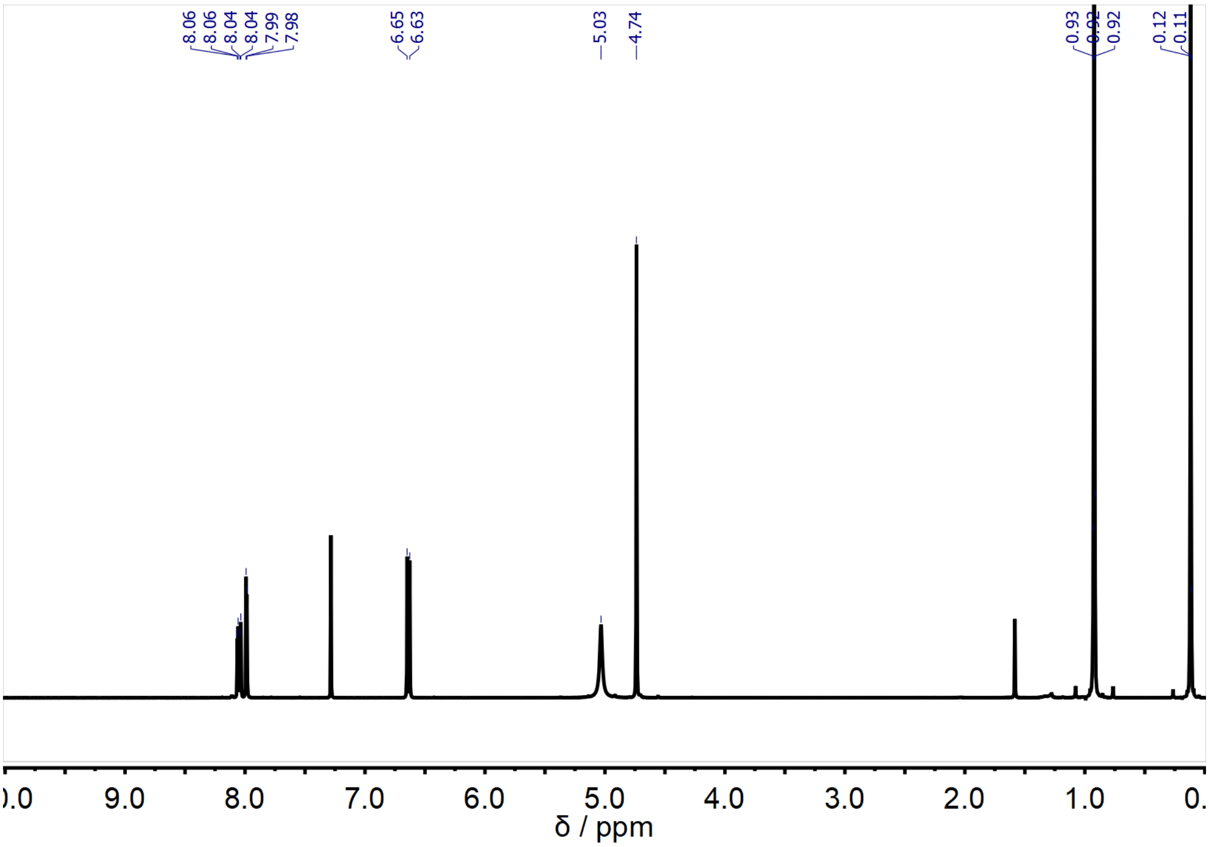

Figure S.24 – ^{1}H NMR spectrum of **8** in $CDCl_3$ (δ 0 – 11 ppm).

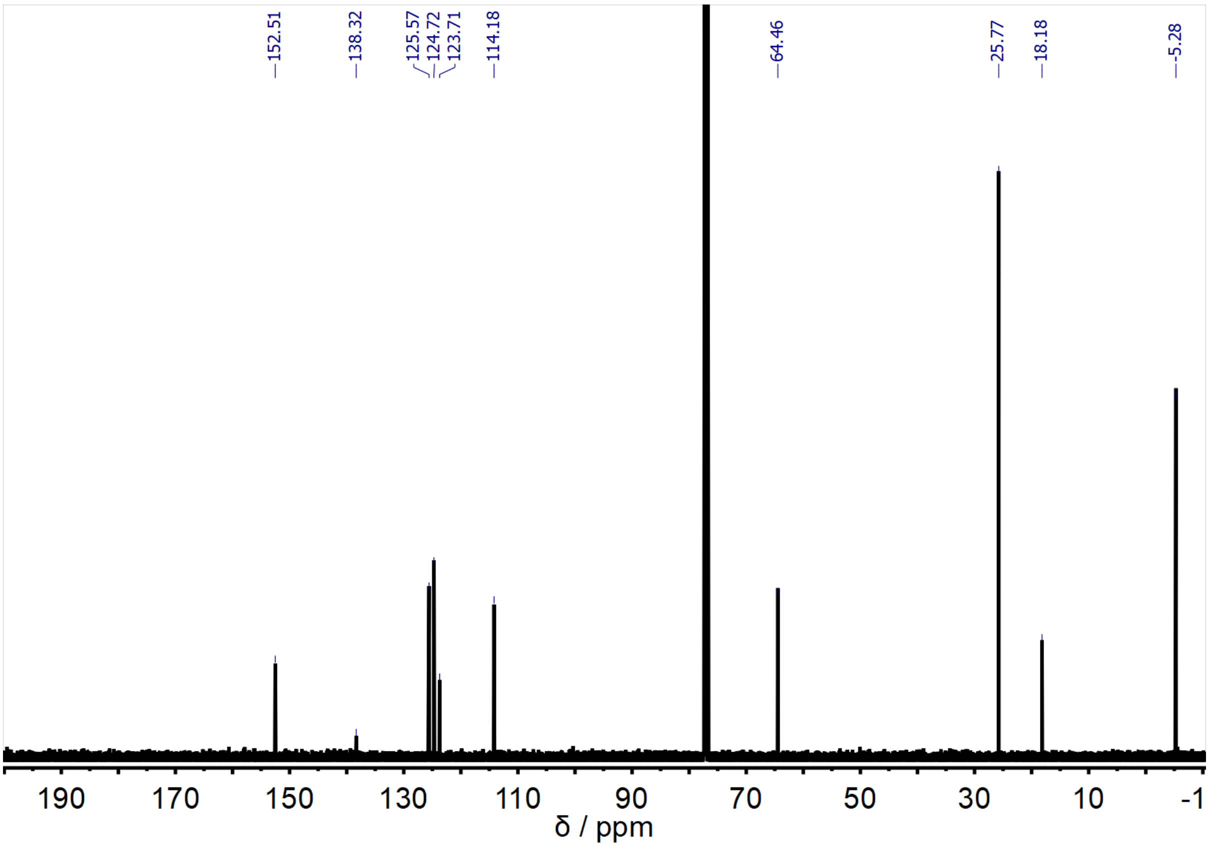

Figure S.25 – ^{13}C NMR spectrum of **8** in $CDCl_3$ (δ -10 – 200 ppm).

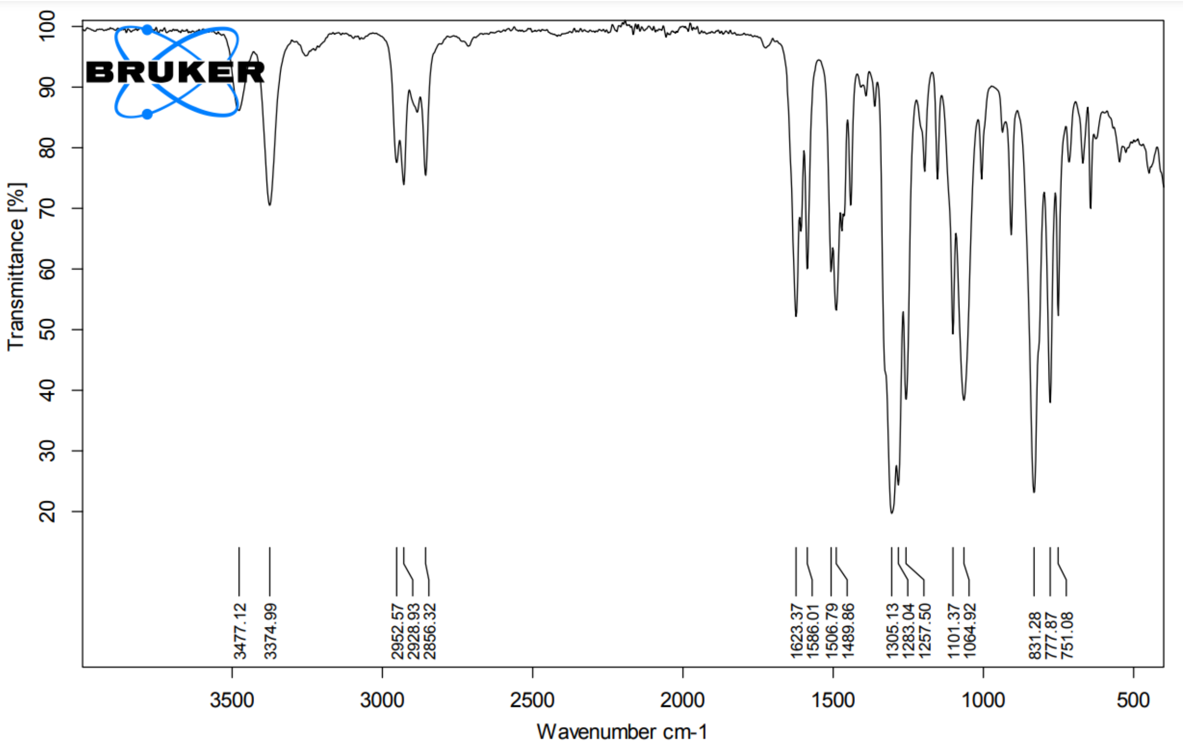

Figure S.26 – FT-IR spectrum of **8**

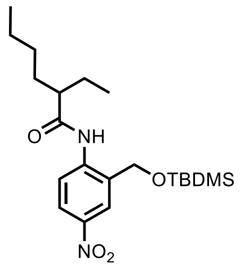

N-(2-(((*tert*-butyldimethylsilyl)oxy)methyl)-4-nitrophenyl)-2-ethylhexanamide, **9**. 2-(((*tert*-butyldimethylsilyl)oxy)methyl)-4-nitroaniline (0498 g, 1.76 mmol) was dissolved in dry DCM (5 mL) under an inert atmosphere. Triethylamine (0.70 mL, 5.0 mmol) and 2-ethylhexanoyl chloride (0.46 mL, 2.7 mmol) were added and the reaction stirred for 3 days. The reaction mixture was diluted with EtOAc (50 mL) and washed with 1M NaOH (2 x 25 mL), saturated $NaHCO_3$ (2 x 25 mL) and brine (25 mL). The organic layer was dried with $MgSO_4$ and solvent removed in vacuo. The crude mixture was purified by flash chromatography to yield desired product as a yellow solid (0.370 g, 0.906 mmol, 51%)

ν_{max} (film) cm^{-1} : 3343 (N-H), 2957 (C-H), 2931 (C-H), 2859 (C-H), 1698 (C=O), 1505 (C=C), 1340, 1278, 1263, 1060, 906, 832, 780, 729.

^{1}H NMR (400 MHz, $CDCl_3$) δ_H ppm: 9.23 (s, 1H), 8.53 (d, J = 9.0 Hz, 1H), 8.20 (dd, J = 9.1, 2.7 Hz, 1H), 8.00 (d, J = 2.6 Hz, 1H), 4.80 (s, 2H), 2.12 (tt, J = 9.0, 5.2 Hz, 1H), 1.79 – 1.65 (m, 2H), 1.65 – 1.49 (m, 2H), 1.39 – 1.26 (m, 4H), 0.97 (t, J = 7.4 Hz, 3H), 0.93 (s, 9H), 0.89 (t, J = 7.1, 6.7 Hz, 3H), 0.14 (s, 6H).

$^{13}C\{^1H\}$ NMR (101MHz, $CDCl_3$) δ_c ppm: 175.0, 144.3, 142.7, 128.8, 124.9, 123.5, 121.3, 65.3, 51.6, 32.6, 30.0, 26.3, 25.9, 22.9, 18.4, 14.1, 12.2, -5.1z.

HRMS: calc for $C_{21}H_{35}N_2O_4Si^+$ $[M-H]^-$: 407.2366, found: 407.2232

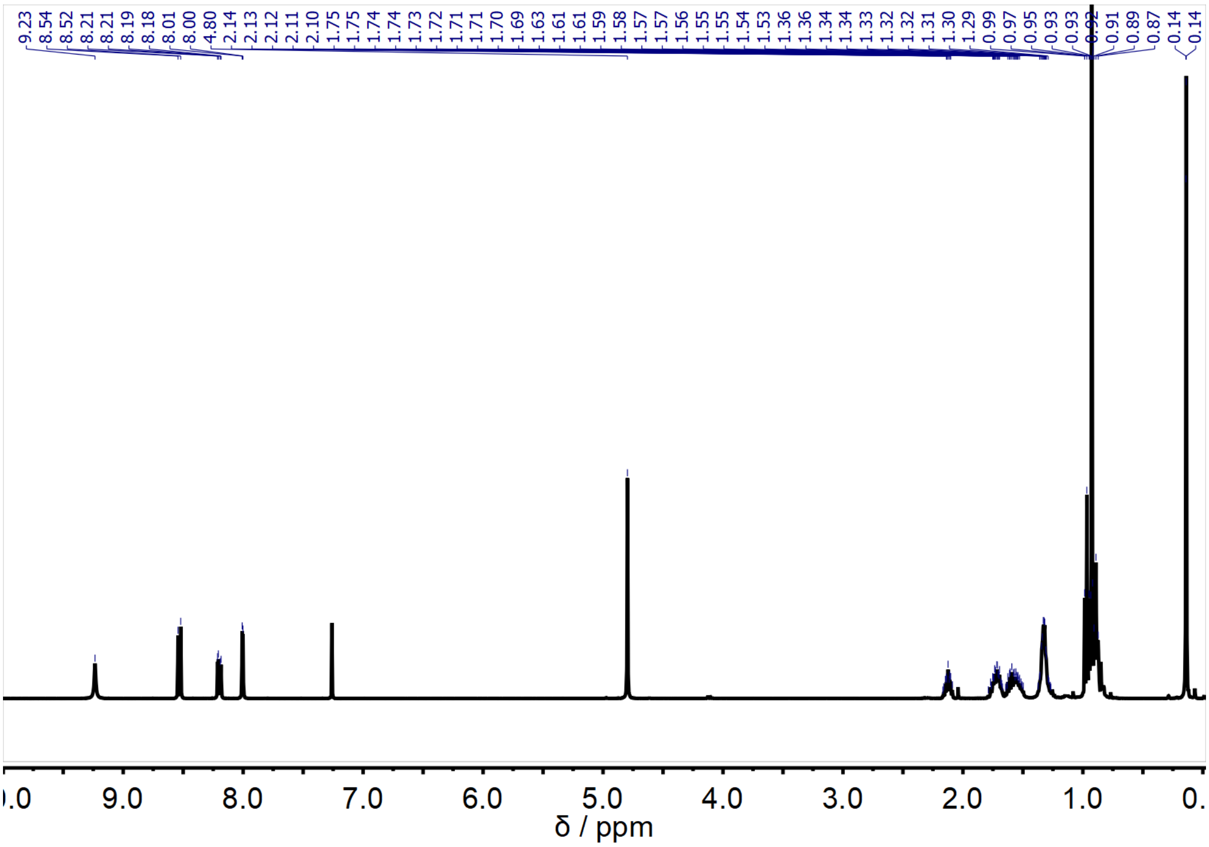

Figure S.27 – ^{1}H NMR spectrum of **9** in $CDCl_3$ (δ 0 – 10 ppm).

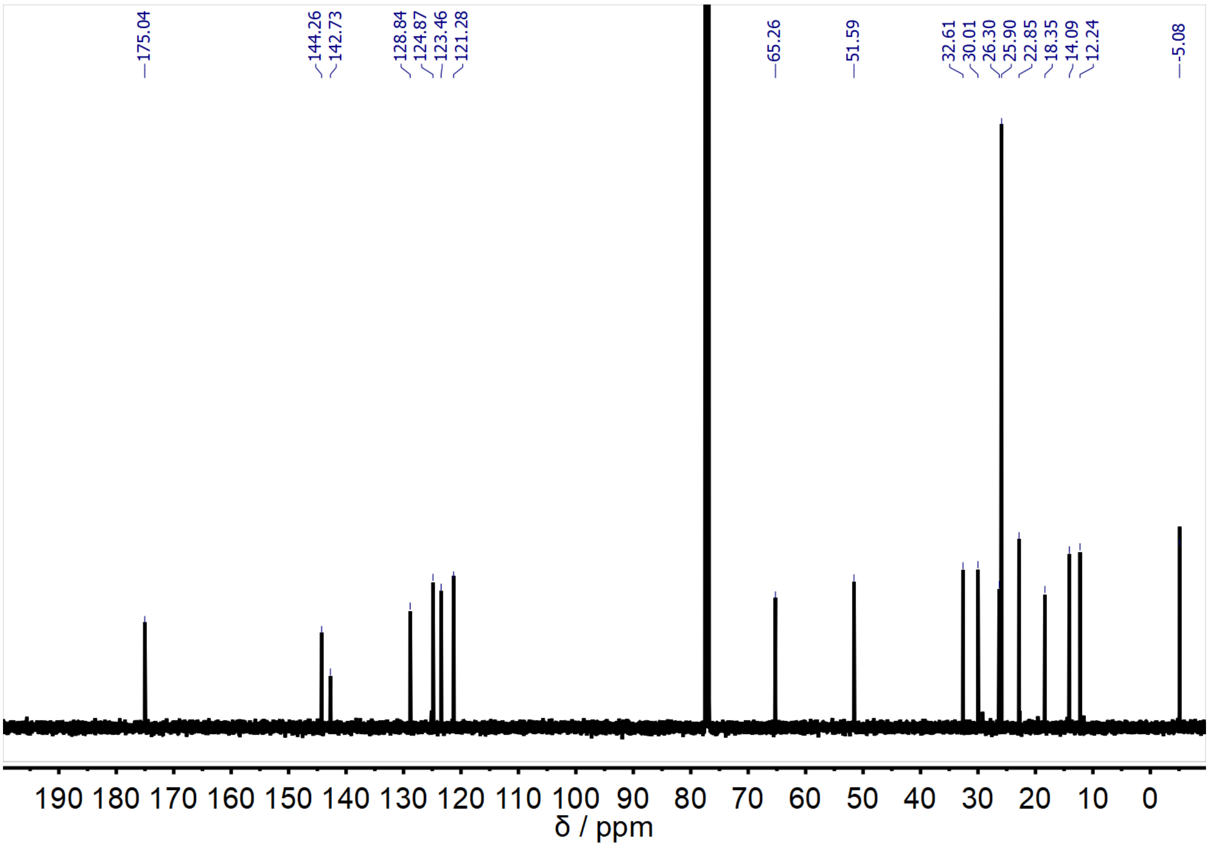

Figure S.28 – ^{13}C NMR spectrum of **9** in $CDCl_3$ (δ -10 – 200 ppm).

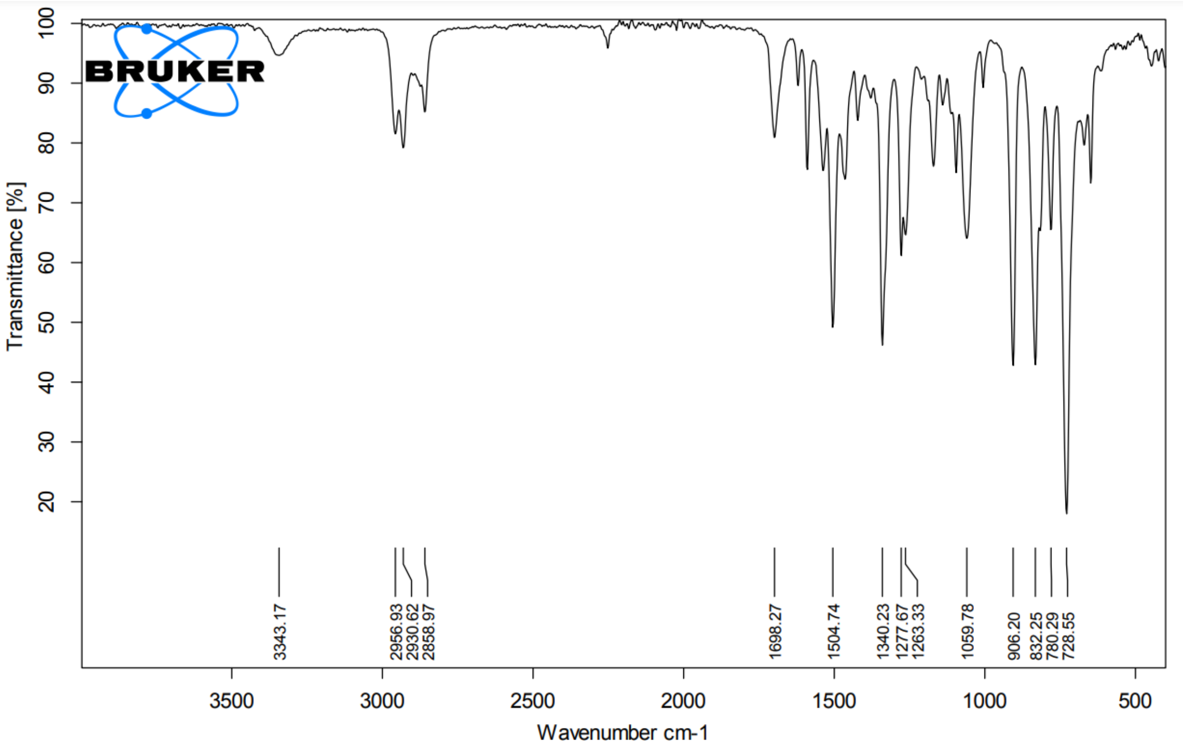

Figure S.29 – FT-IR spectrum of **9**

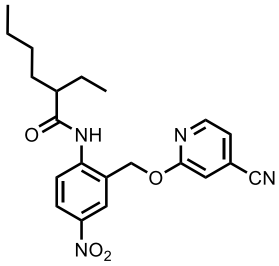

N-(2-(((4-cyanopyridin-2-yl)oxy)methyl)-4-nitrophenyl)-2-ethylhexanamide, **10**. N-(2-(((*tert*-butyldimethylsilyl)oxy)methyl)-4-nitrophenyl)-2-ethylhexanamide (0.196 g, 0.481 mmol) was dissolved in dry THF (5 mL) under an inert atmosphere. TBAF (1 M in THF, 0.5 mL, 0.5 mmol) was added and reaction stirred for 30 minutes. 4-cyano-2-fluoropyridine (0.183 g, 1.50 mmol) dissolved in dry THF (1.5 mL) was added to the reaction and stirred for 1 day. The reaction mixture was quenched with 1M HCl (1 mL) and then diluted with EtOAc (50 mL). The mixture was then washed with 1M HCl (25 mL), H_2O (2 x 25 mL) and brine (25 mL). The organic layer was dried in $MgSO_4$ and solvent removed in vacuo. The crude product was purified by flash chromatography (PE/EtOAc on Al_2O_3) to yield the desired product as a yellow solid (0.105 g, 0.264 mmol, 55%).

MP – 135 – 137 ^{O}C (acetone)

ν_{max} (film) cm^{-1} : 3244 (N-H), 2959 (C-H), 2930 (C-H), 2873 (C-H), 1660 (C=O), 1603 (C=C), 1589 (C=C), 1548 (C=C), 1509 (C=C), 1474 (C=C), 1413, 1364, 1342, 1311, 1286, 1273, 1238, 1147, 1041, 745.

^{1}H NMR (400 MHz, $CDCl_3$) δ_H ppm: 9.28 (s, 1H), 8.38 (s, 1H), 8.37 (d, J = 5.4 Hz, 1H), 8.33 (d, J = 5.2 Hz, 1H), 8.22 (dd, J = 9.1, 2.7 Hz, 1H), 7.20 (dd, J = 5.3, 1.3 Hz, 1H), 7.10 (s, 1H), 5.49 (s, 2H), 1.84-1.70 (m, 2H), 1.76 (m, 2H), 1.68-1.54 (m, 2H), 1.39 – 1.30 (m, 4H), 0.99 (t, J = 7.4 Hz, 3H), 0.89 (t, J = 5.6, 4.4 Hz, 3H).

^{13}C{^{1}H} NMR (101MHz, $CDCl_3$) δ_C ppm: 174.9, 162.8, 147.9, 143.5, 143.0, 127.3, 125.9, 125.5, 124.0, 123.0, 119.2, 115.9, 115.1, 65.1, 51.3, 32.6, 30.1, 26.3, 22.9, 14.1, 12.3.

HRMS: calc for $C_{21}H_{25}N_4O_4^+$ $[M+H]^+$: 397.1876, found: 397.1890

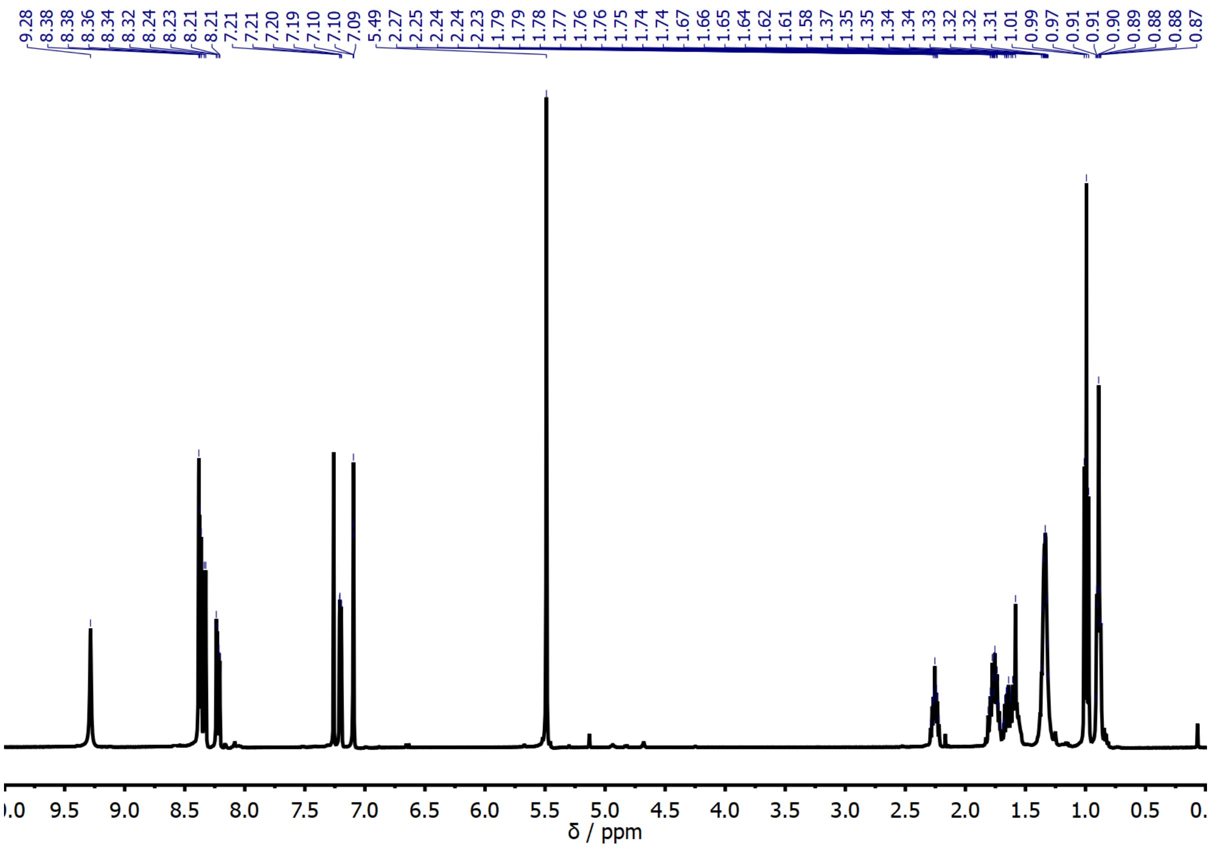

Figure S.30 – ^{1}H NMR spectrum of **10** in $CDCl_3$ (δ 0 – 10 ppm).

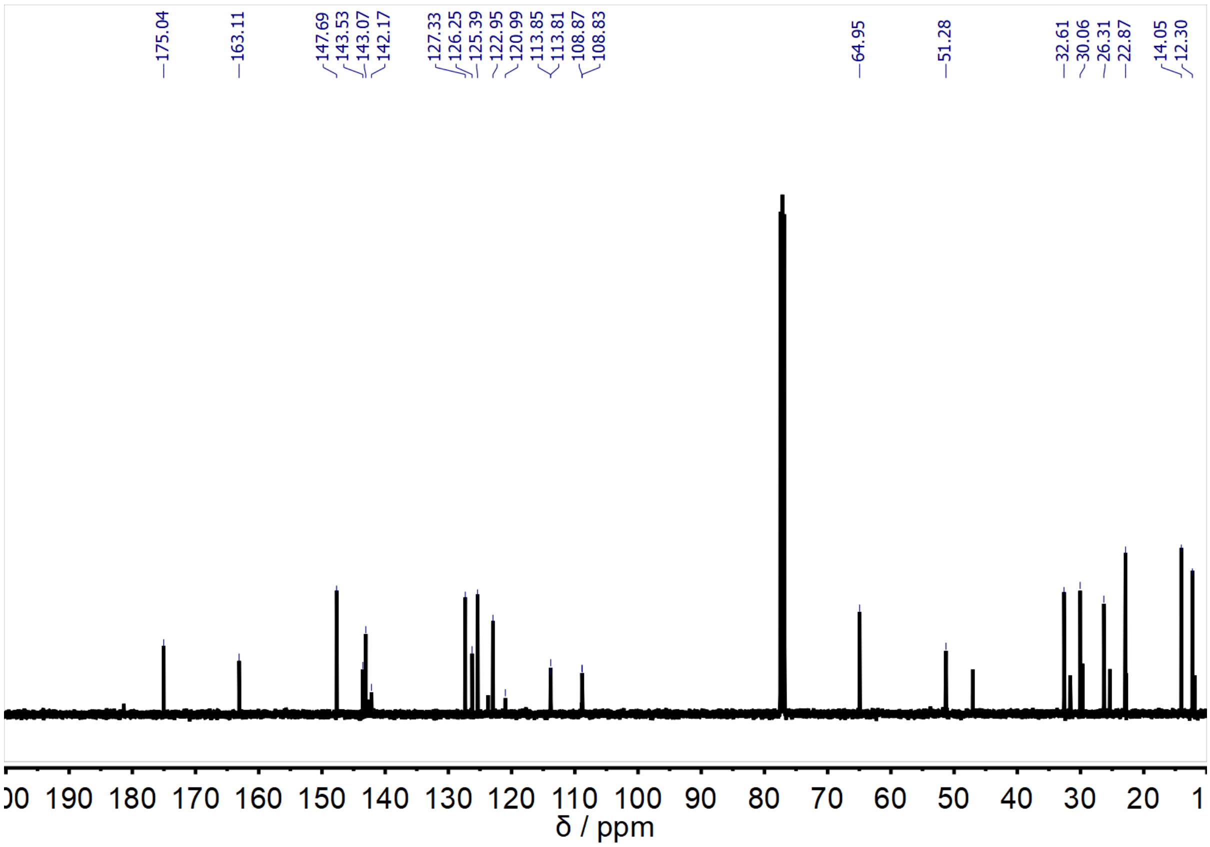

Figure S.31 – ^{13}C NMR spectrum of **10** in $CDCl_3$ (δ 0 – 200 ppm).

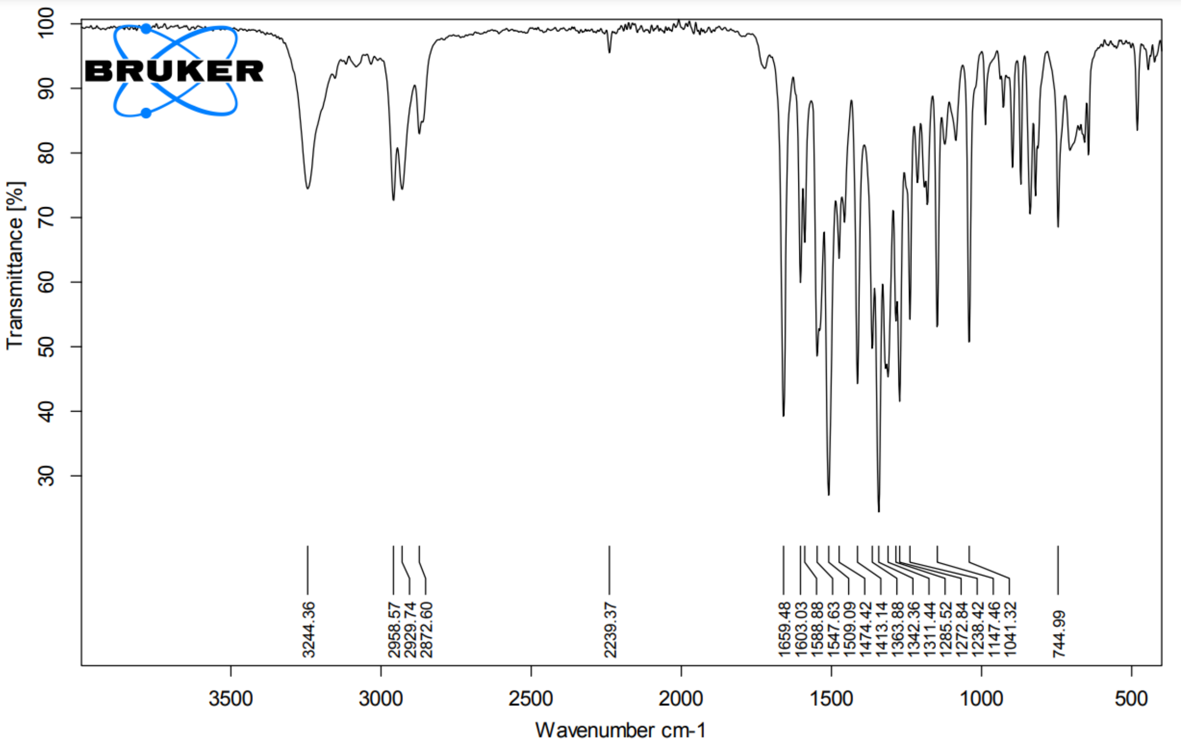

Figure S.32 – FT-IR spectrum of **10**

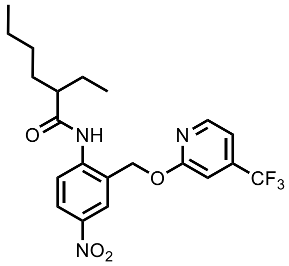

2-ethyl-N-(2-(((4-(trifluoromethyl)pyridin-2-yl)oxy)methyl)-4-nitrophenyl)hexanamide, **11**. 4-(trifluoromethyl)-2-((pyridin-2-yloxy)methyl)aniline (49.0 mg, 0.156 mmol) and NEt_3 (30 µL, 0.215 mmol) were dissolved in dry DCM (2 mL) under a N_2 atmosphere. 2-ethylhexanoyl chloride (30 µL, 0.215 mmol) was added and the reaction stirred overnight. The reaction mixture was diluted with EtOAc (50 mL), and then washed with 1M NaOH (25 mL), sat. $NaHCO_3$ (2 x 25 mL) and brine (25 mL). The organic layer was dried with $MgSO_4$ and in vacuo. The crude product was purified by flash chromatography (PE/EtOAc on SiO_2) to yield the desired product as a white solid (38.3 mg, 0.0872 mmol, 56%)

ν_{max} (film) cm^{-1} : 3251 (N-H), 2961 (C-H), 2931 (C-H), 2861 (C-H), 1659 (C=O), 1509 (C=C), 1417 (C=C), 1366, 1338, 1271, 1175, 1132, 1079, 1035, 907, 877, 819, 733, 671

^{1}H NMR (400 MHz, $CDCl_3$) δ_H ppm: δ 9.57 (s, 1H), 8.39 (s, 1H), 8.38 (d, J = 6.1 Hz, 1H), 8.34 (d, J = 5.4 Hz, 1H), 8.22 (dd, J = 9.1, 2.7 Hz, 1H), 7.20 (dd, J = 5.4, 1.4 Hz, 1H), 7.07 (d, J = 1.4 Hz, 1H), 5.50 (s, 2H), 2.28 (tt, J = 8.9, 5.2 Hz, 1H), 1.87 – 1.71 (m, 2H), 1.70 – 1.53 (m, 2H), 1.33 (dtt, J = 11.8, 8.3, 4.5 Hz, 4H), 1.00 (t, J = 7.4 Hz, 3H), 0.89 – 0.86 (m, 3H).

$^{13}C\{^1H\}$ NMR (101MHz, $CDCl_3$) δ_C ppm: 175.0, 163.1, 147.7, 143.5, 143.1, 142.2, 127.3, 126.2, 125.4, 123.0, 121.0, 113.9, 113.8, 108.9, 108.8, 65.0, 51.3, 32.6, 30.1, 26.3, 22.9, 14.1, 12.3.

HRMS: calc for $C_{21}H_{25}N_3O_4F_3^+$ $[M+H]^+$: 440.1797, found: 440.1787

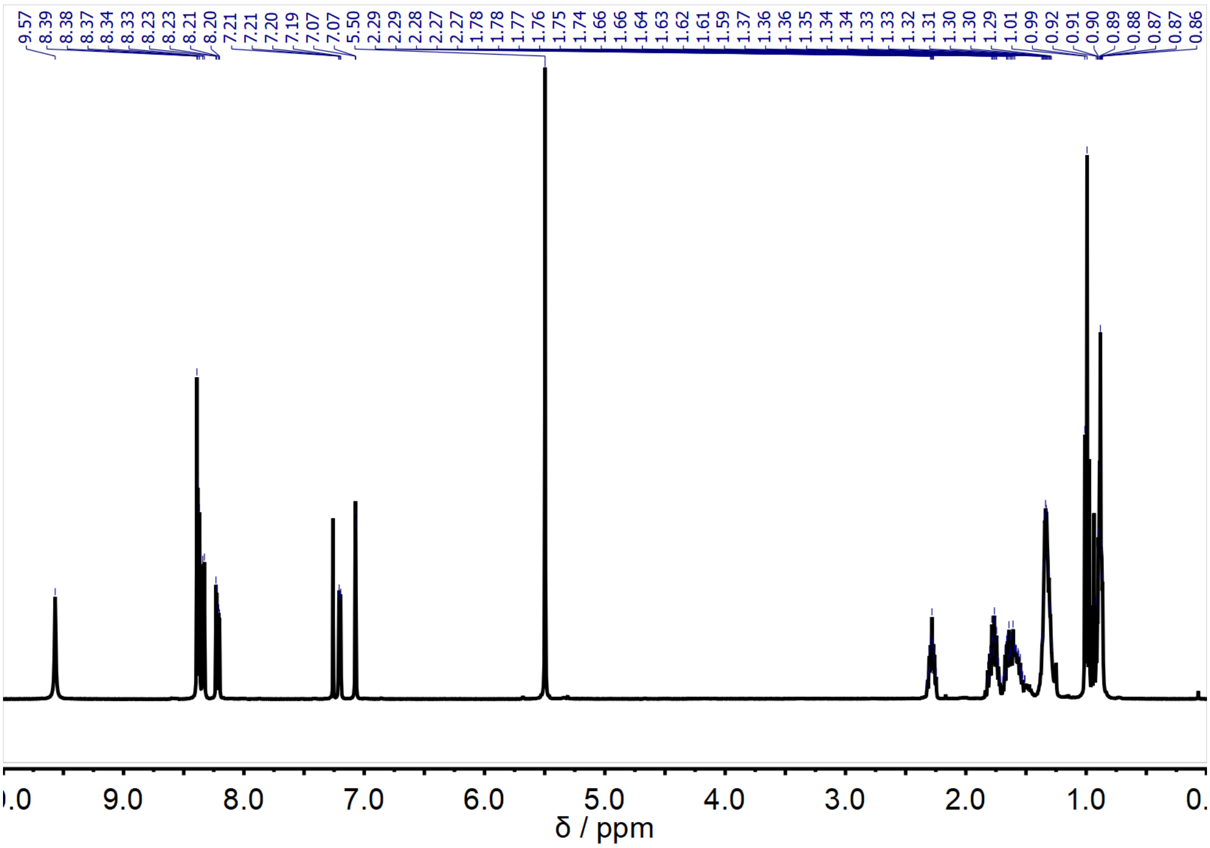

Figure S.33 – ^{1}H NMR spectrum of **11** in $CDCl_3$ (δ 0 – 11 ppm).

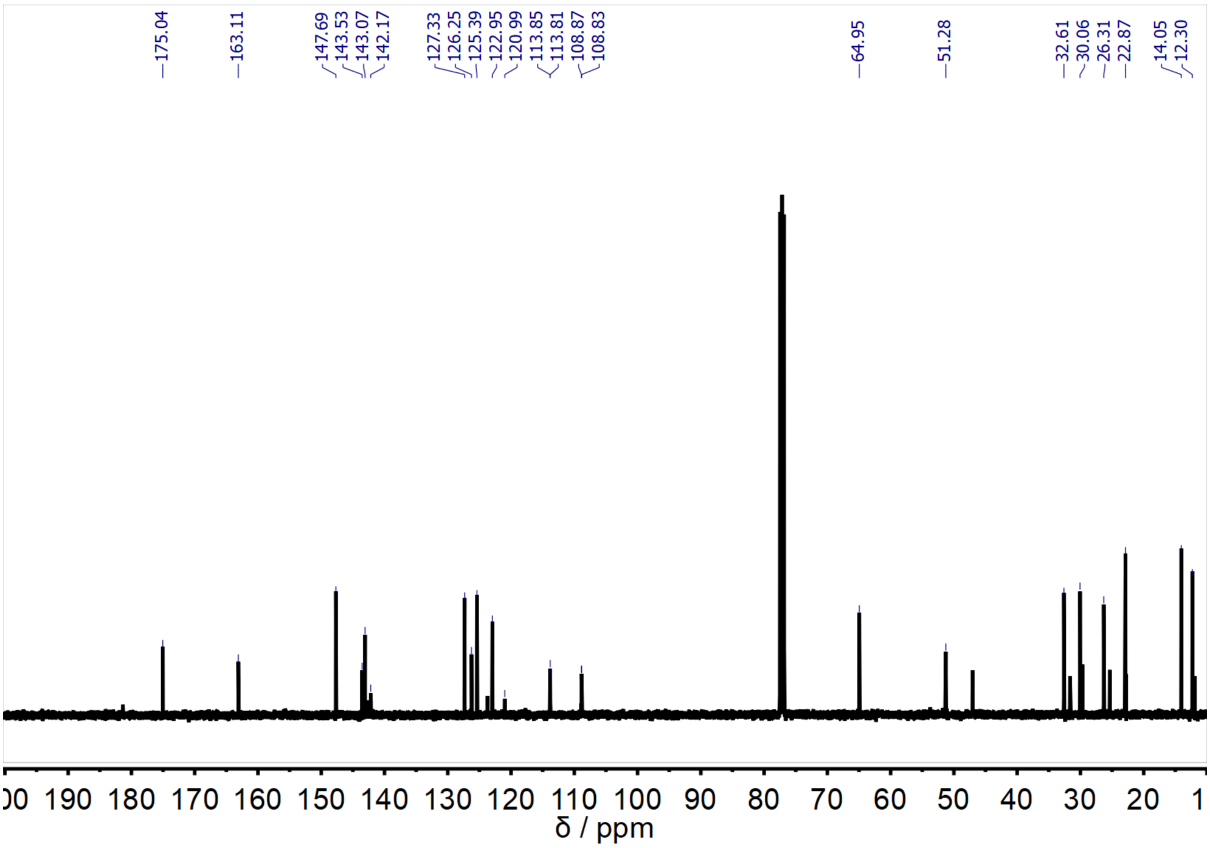

Figure S.34 – ^{13}C NMR spectrum of **11** in $CDCl_3$ (δ 0 – 200 ppm).

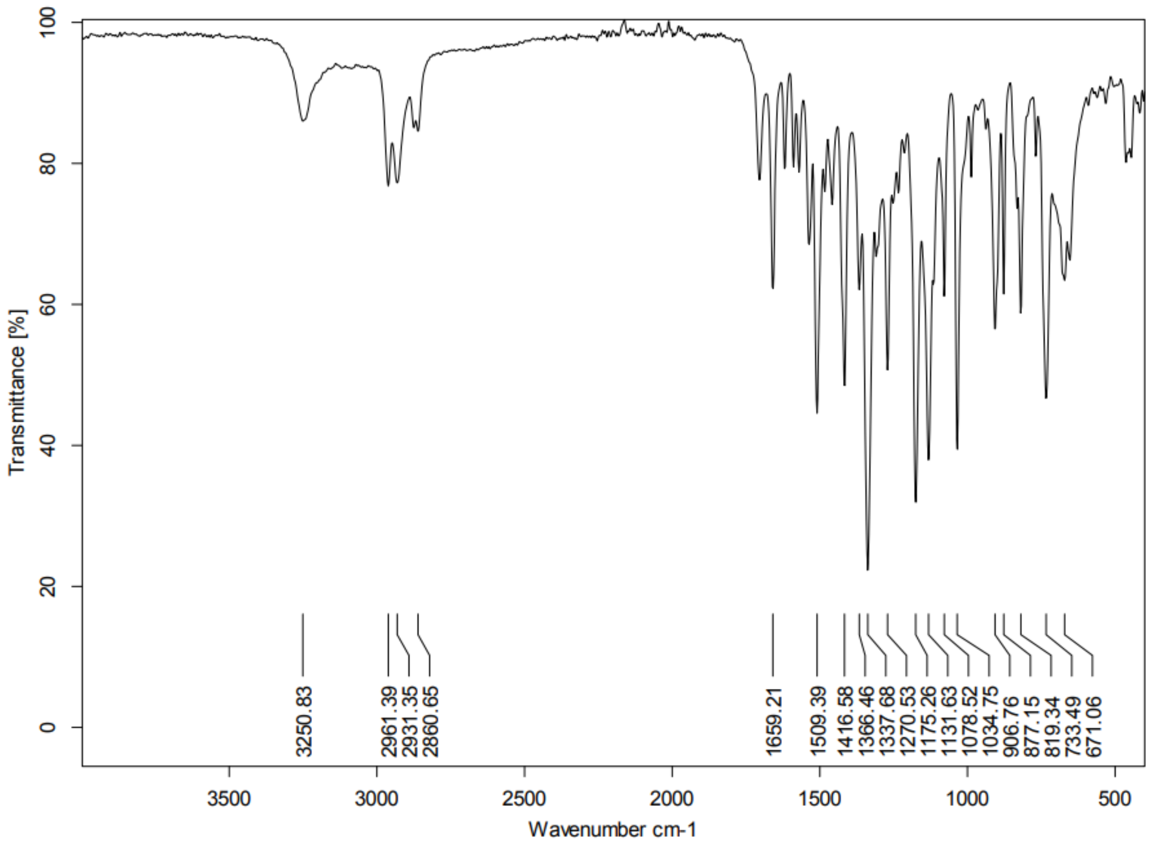

Figure S.35 – FT-IR spectrum of **11**

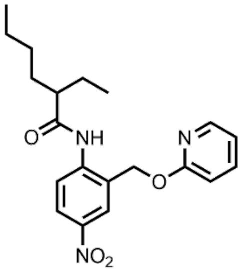

2-ethyl-N-(4-nitro-2-((pyridin-2-yloxy)methyl)phenyl)hexanamide, **12**. 4-nitro-2-((pyridin-2-yloxy)methyl)aniline (7.0 mg, 0.029 mmol) was dissolved in dry DCM (0.5 mL) under a nitrogen atmosphere. NEt_3 (10 µL, 0.071 mmol) and 2-ethylhexanoylchloride (5.0 µL, 0.029 mmol) were and the reaction stirred for 2 hours. The reaction mixture was diluted with EtOAc (50 mL) and washed with NaOH (25 mL), $NaHCO_3$ (25 mL) and water (3 x 10 mL). The organic layer was dried with $MgSO_4$, and the solvent removed under reduced pressure to obtain the desired product as a yellow solid (4.6 mg, 0.012 mmol, 43%)

ν_{max} (film) cm^{-1} : 3257 (N-H), 2959 (C-H), 2928 (C-H), 2858 (C-H), 1665 (C=O), 1591 (C=C), 1537 (C=C), 15-5 (C=C), 1471 (C=C), 1432 (C=C), 1343, 1313, 1302, 1272, 1252, 1090, 1049, 814, 778, 745, 736

^1H NMR (400 MHz, $CDCl_3$) δ_H ppm: δ 10.31 (s, 1H), 8.39 (s, 1H), 8.38 (d, J = 7.0 Hz, 1H), 8.21 (dd, J = 9.1, 2.7 Hz, 1H), 8.17 (ddd, J = 5.2, 2.0, 0.8 Hz, 1H), 7.67 (ddd, J = 8.8, 7.1, 1.9 Hz, 1H), 7.00 (ddd, J = 7.1, 5.2, 1.0 Hz, 1H), 6.84 (dt, J = 8.4, 0.9 Hz, 1H), 5.43 (d, J = 2.4 Hz, 2H), 2.33 (tt, J = 9.4, 5.1 Hz, 1H), 1.86 – 1.72 (m, 2H), 1.68 – 1.59 (m, 1H), 1.59 – 1.54 (m, 2H), 1.34 (pdd, J = 9.2, 5.0, 2.7 Hz, 4H), 1.25 (s, 1H), 0.99 (t, J = 7.4 Hz, 3H), 0.88 (h, J = 3.4, 2.9 Hz, 4H).

$^{13}C\{^1H\}$ NMR (101 MHz, $CDCl_3$) δ_C ppm: 175.27, 162.72, 145.93, 143.44, 143.31, 140.13, 127.57, 127.04, 125.19, 122.95, 118.11, 112.29, 64.25, 51.13, 32.62, 30.06, 26.34, 22.91, 14.11, 12.32.

HRMS: calc for $C_{20}H_{26}N_3O_4^+$ $[M+H]^+$: 372.1923, found: 372.1921

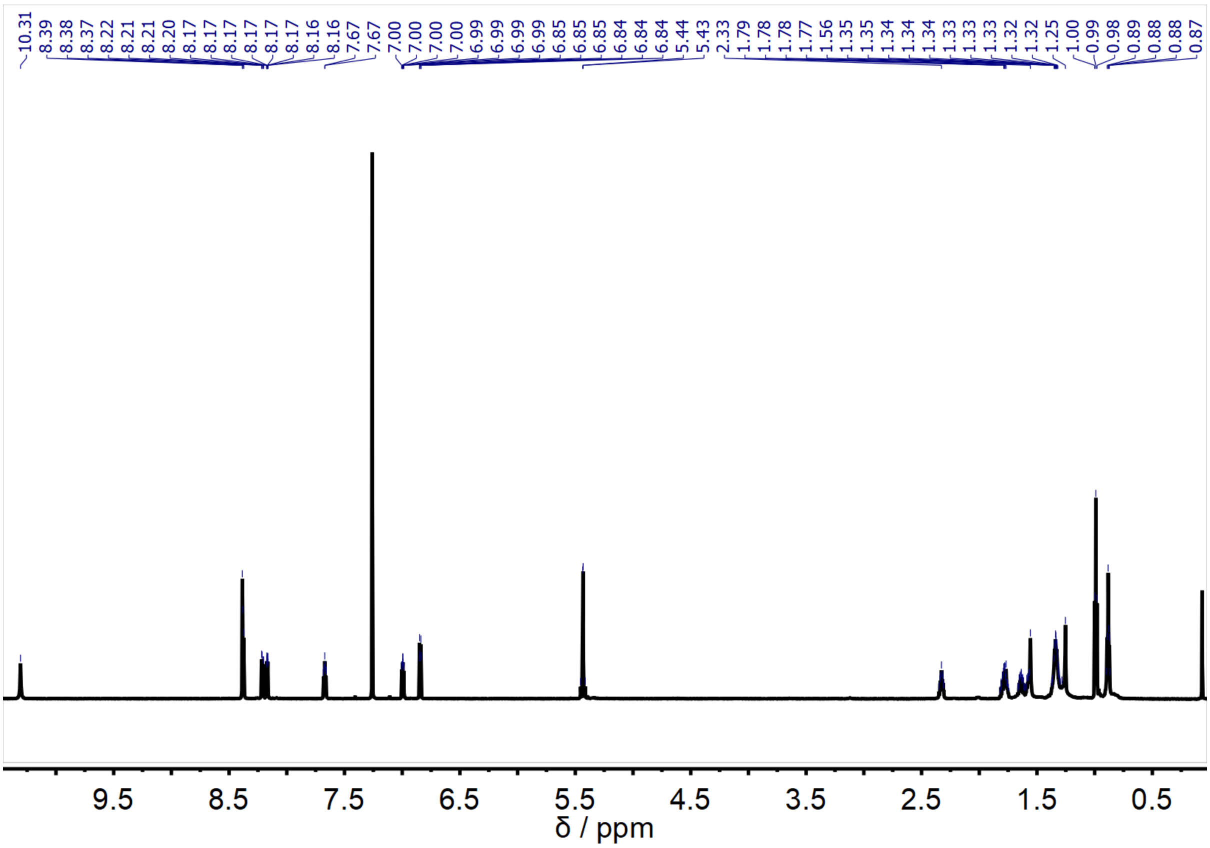

Figure S.36 – ^{1}H NMR spectrum of **12** in $CDCl_3$ (δ 0 – 10.5 ppm).

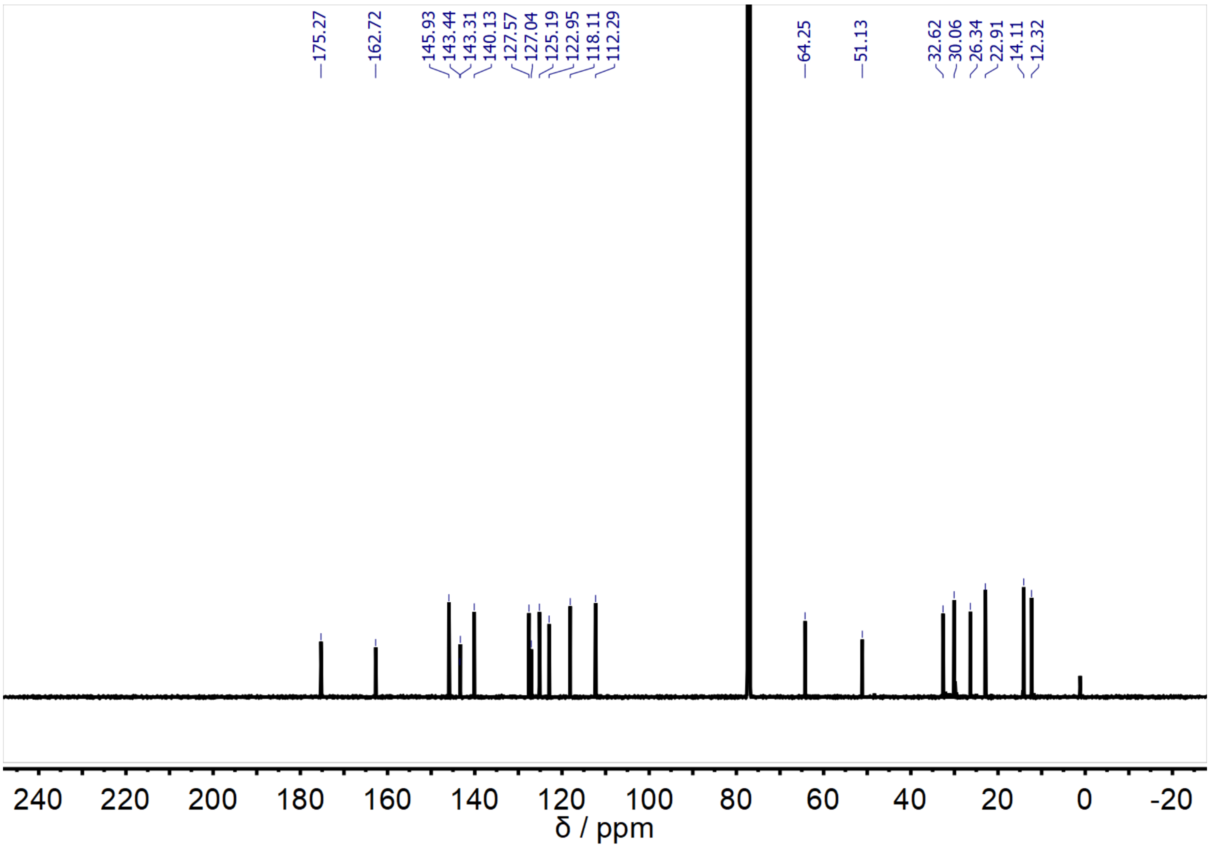

Figure S.37 – ^{13}C NMR spectrum of **12** in $CDCl_3$ (δ 0 – 200 ppm).

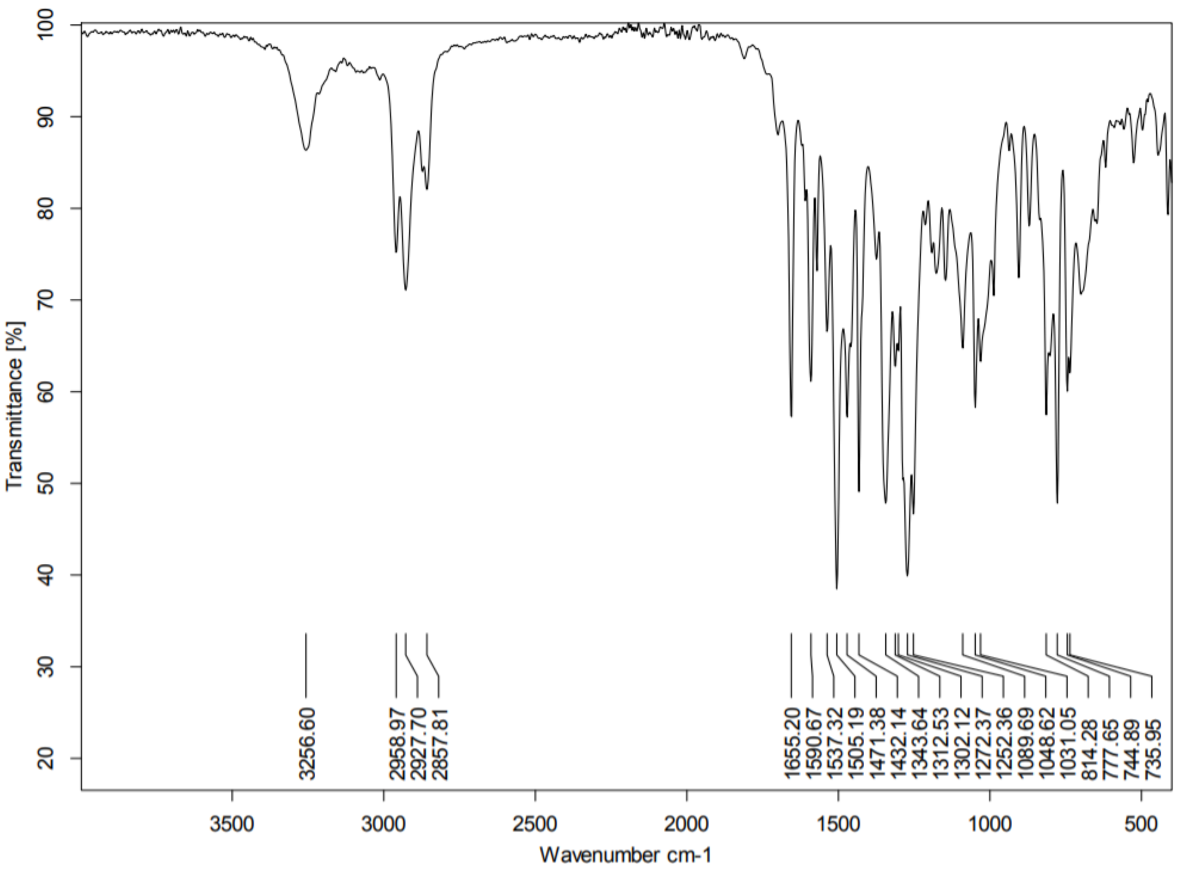

Figure S.38 – FT-IR spectrum of **12**

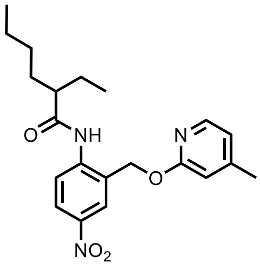

2-ethyl-N-(2-(((4-methylpyridin-2-yl)oxy)methyl)-4-nitrophenyl)hexanamide, **13**. 4-methyl-2-((pyridin-2-yloxy)methyl)aniline (10.8 mg, 0.0416 mmol) and NEt_3 (20 µL, 0.144 mmol) were dissolved in dry DCM (0.5 mL) under a N_2 atmosphere. 2-ethylhexanoyl chloride (7.5 µL, 0.0433 mmol) was added and the reaction stirred for 5 hours. The reaction mixture was diluted with EtOAc (50 mL), and then washed with 1M NaOH (25 mL), sat. $NaHCO_3$ (25 mL) and brine (25 mL). The organic layer was dried with $MgSO_4$ and in vacuo. The crude product was purified by flash chromatography (PE/EtOAc on SiO_2) to yield the desired product as a white solid (6.6 mg, 0.0171 mmol, 41%)

ν_{max} (film) cm^{-1} : 3207 (N-H), 2961 (C-H), 2930 (C-H), 2859 (C-H), 1698 (C=O), 1615 (C=C), 1589 (C=C), 1508 (C=C), 1339, 1280, 1172, 1160, 908, 730

^1H NMR (400 MHz, $CDCl_3$) δ_H ppm: δ 10.53 (s, 1H), 8.38 (d, J = 0.9 Hz, 1H), 8.36 (d, J = 7.4 Hz, 1H), 8.20 (dd, J = 9.1, 2.7 Hz, 1H), 8.03 – 7.98 (m, 1H), 6.81 (ddd, J = 5.3, 1.4, 0.7 Hz, 1H), 6.65 (dt, J = 1.5, 0.8 Hz, 1H), 5.40 (s, 2H), 2.38 – 2.33 (m, 1H), 2.32 (s, 3H), 1.86 – 1.71 (m, 2H), 1.68 – 1.58 (m, 1H), 1.39 – 1.30 (m, 5H), 0.99 (t, J = 7.4 Hz, 3H), 0.88 (td, J = 5.6, 4.2, 2.7 Hz, 3H).

$^{13}C\{^1H\}$ NMR (101MHz, $CDCl_3$) δ_C ppm: δ 175.2, 162.8, 151.7, 145.1, 143.2, 127.5, 127.1, 125.0, 122.8, 119.5, 112.1, 64.0, 50.9, 32.5, 29.9, 26.2, 22.8, 21.0, 14.0, 12.2.

HRMS: calc for $C_{21}H_{28}N_3O_4^+$ $[M+H]^+$: 386.2080, found: 386.2073

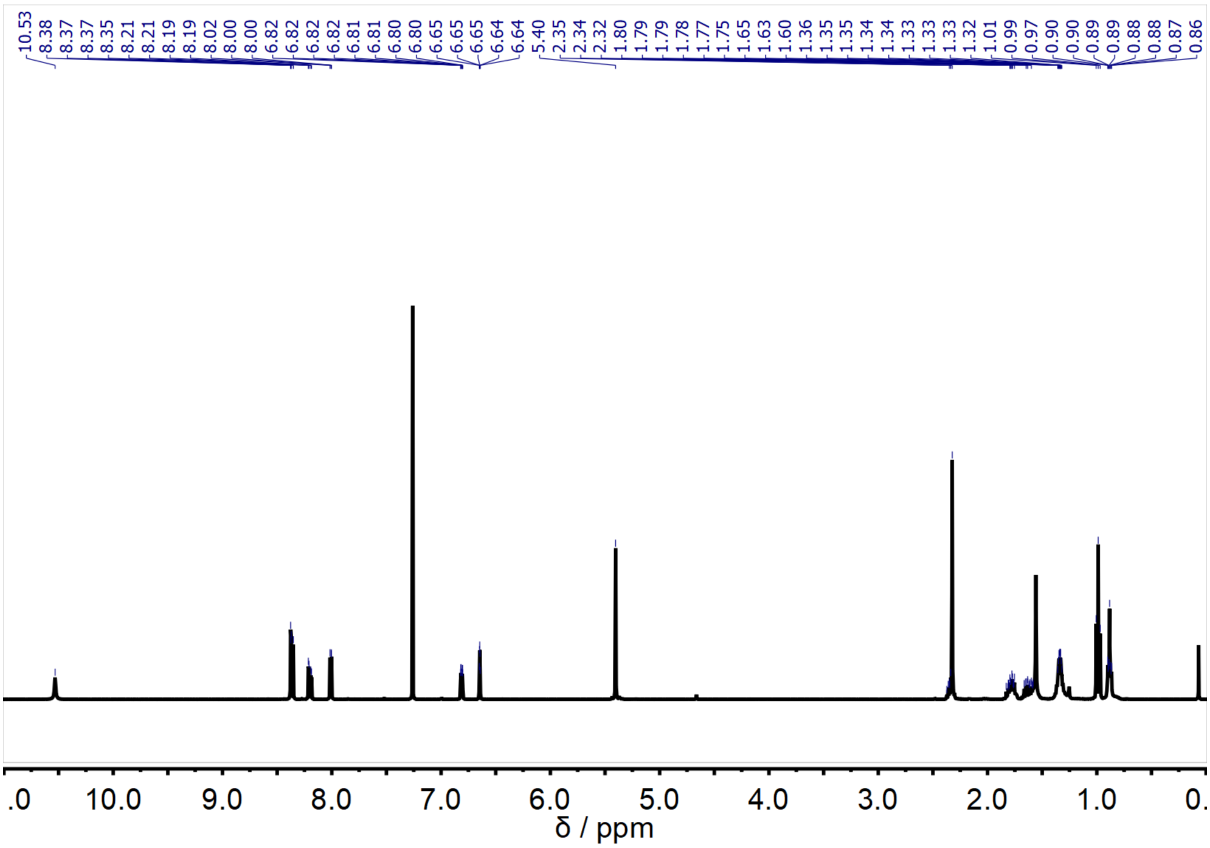

Figure S.39 – ^{1}H NMR spectrum of **13** in $CDCl_3$ (δ 0 – 11 ppm).

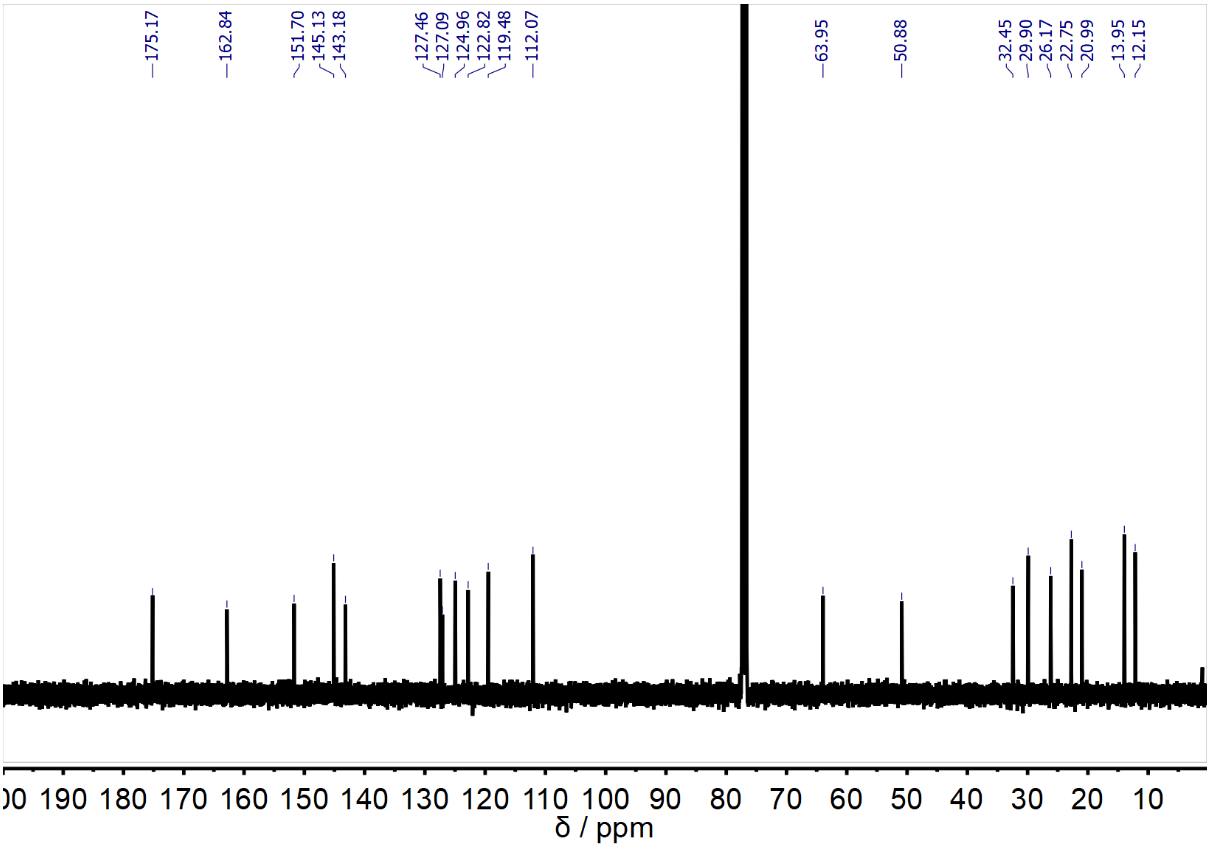

Figure S.40 – ^{13}C NMR spectrum of **13** in $CDCl_3$ (δ 0 – 200 ppm).

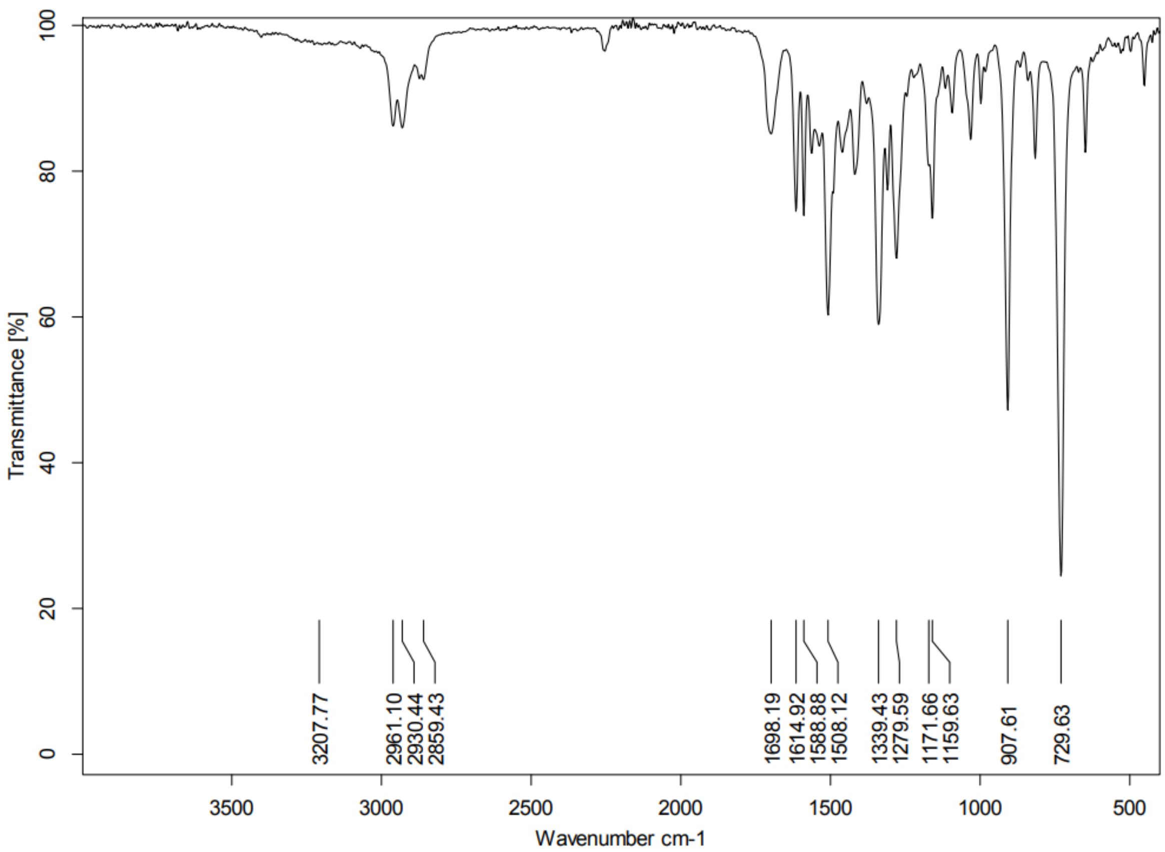

Figure S.41 – FT-IR spectrum of **13**

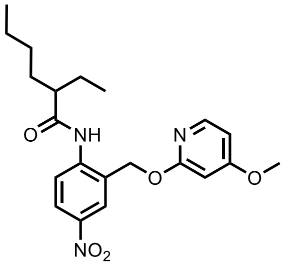

2-ethyl-N-(2-(((4-methoxypyridin-2-yl)oxy)methyl)-4-nitrophenyl)hexanamide, **14**. 4-methoxy-2-((pyridin-2-yloxy)methyl)aniline (52.1 mg, 0.189 mmol) and NEt_3 (100 µL, 0.718 mmol) were dissolved in dry DCM (3 mL) under a N_2 atmosphere. 2-ethylhexanoyl chloride (35 µL, 0.202 mmol) was added and the reaction stirred for 5 hours. The reaction mixture was diluted with EtOAc (50 mL), and then washed with 1M NaOH (25 mL), sat. $NaHCO_3$ (25 mL) and brine (25 mL). The organic layer was dried with $MgSO_4$ and in vacuo. The crude product was purified by flash chromatography (PE/EtOAc on SiO_2) to yield the desired product as a white solid (16.0 mg, 0.0399 mmol, 21%)

ν_{max} (film) cm^{-1} : 3270 (N-H), 2960 (C-H), 2930 (C-H), 2873 (C-H), 2859 (C-H), 1603 (C=O), 1589 (C=C), 1571 (C=C), 1506 (C=C), 1486, 1441, 1337, 1273, 1203, 1165, 1092, 1037, 833, 746

^1H NMR (400 MHz, $CDCl_3$) δ_H ppm: δ 10.70 (s, 1H), 8.39 (s, 1H), 8.38 (d, J = 6.5 Hz, 1H), 8.22 (dd, J = 9.1, 2.7 Hz, 1H), 7.97 (d, J = 6.0 Hz, 1H), 6.60 (dd, J = 6.1, 2.2 Hz, 1H), 6.29 (d, J = 2.2 Hz, 1H), 5.43 (s, 2H), 3.85 (s, 3H), 2.36 (tt, J = 9.1, 5.1 Hz, 1H), 1.81 (dddd, J = 16.4, 8.7, 7.4, 4.9 Hz, 2H), 1.71 – 1.54 (m, 3H), 1.42 – 1.31 (m, 4H), 1.01 (t, J = 7.4 Hz, 3H), 0.91 (t, J = 6.8 Hz, 3H).

$^{13}C\{^1H\}$ NMR (101MHz, $CDCl_3$) δ_C ppm: 175.2, 168.7, 164.6, 146.1, 143.3, 143.2, 127.5, 127.1, 125.0, 122.9, 107.6, 94.8, 64.1, 55.5, 50.9, 32.4, 29.9, 26.2, 22.8, 14.0, 12.2.

HRMS: calc for $C_{21}H_{28}N_3O_5^+$ [M+H]$^+$: 402.2028, found: 402.2047

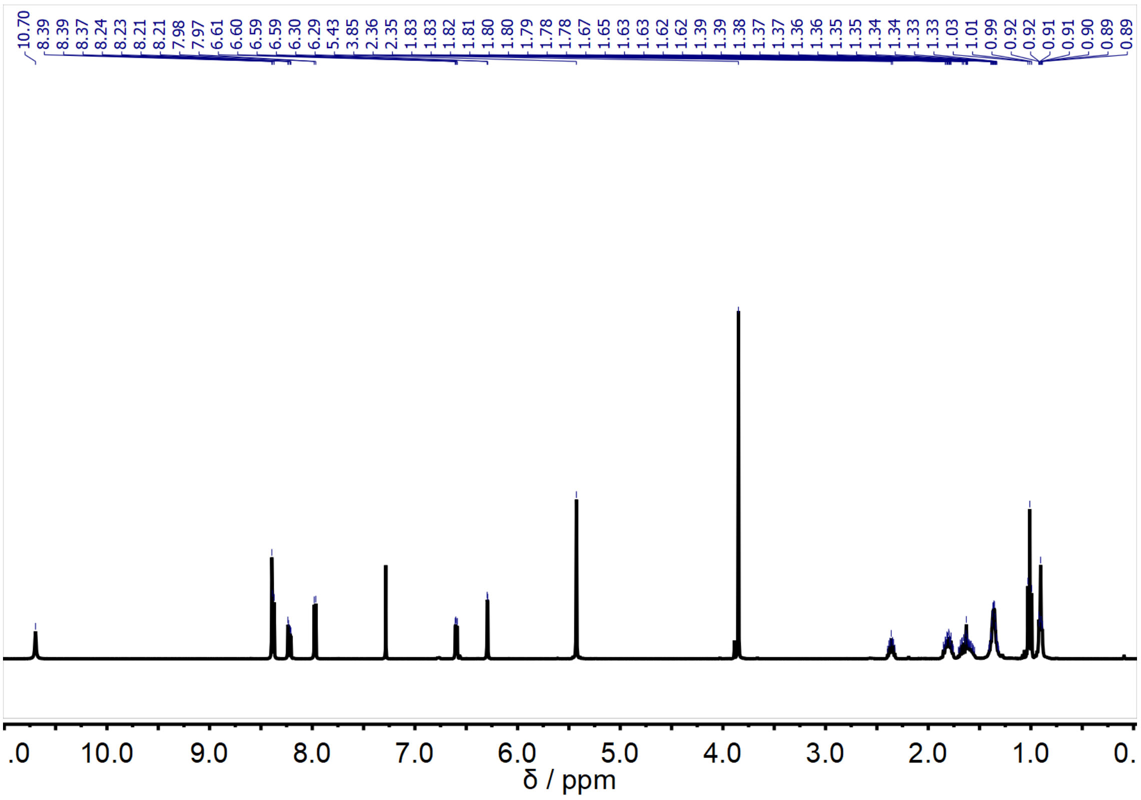

Figure S.42 – ^{1}H NMR spectrum of **14** in $CDCl_3$ (δ 0 – 11 ppm).

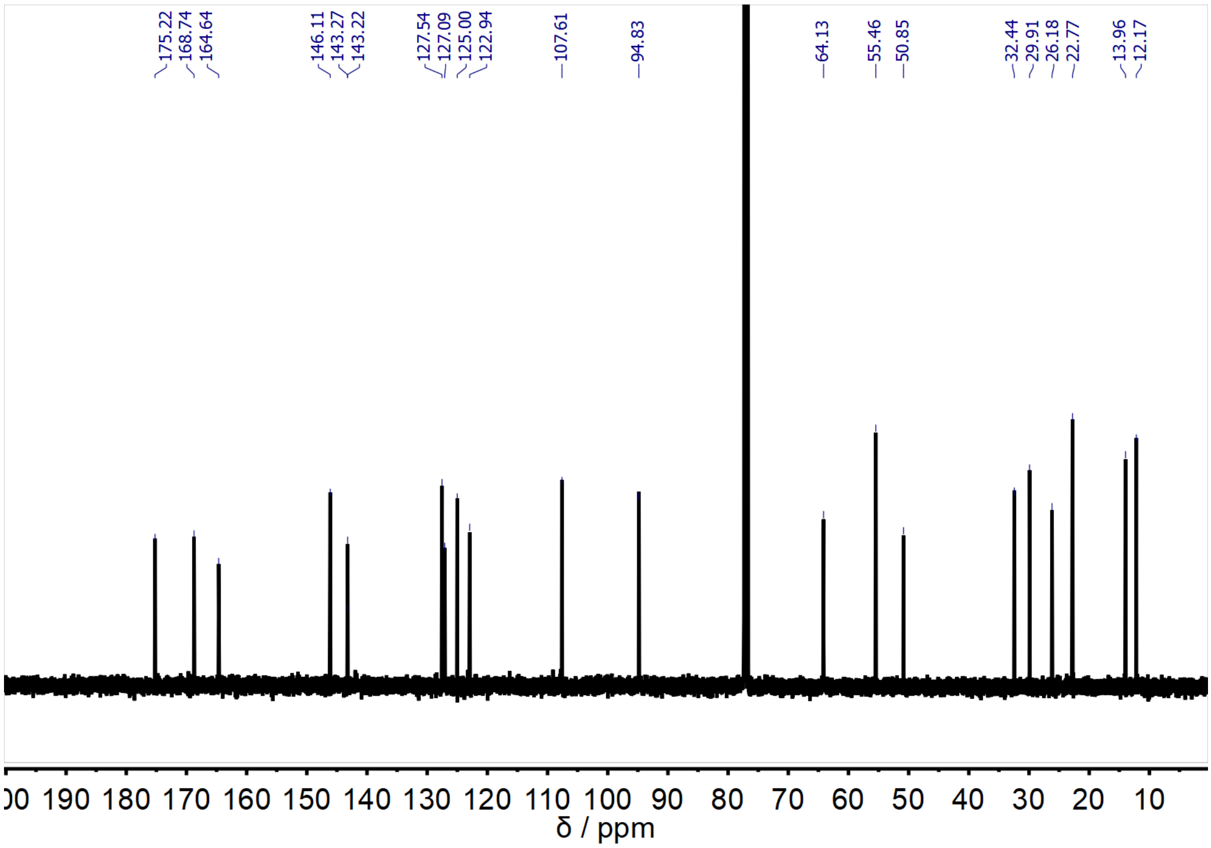

Figure S.43 – ^{13}C NMR spectrum of **14** in $CDCl_3$ (δ 0 – 200 ppm).

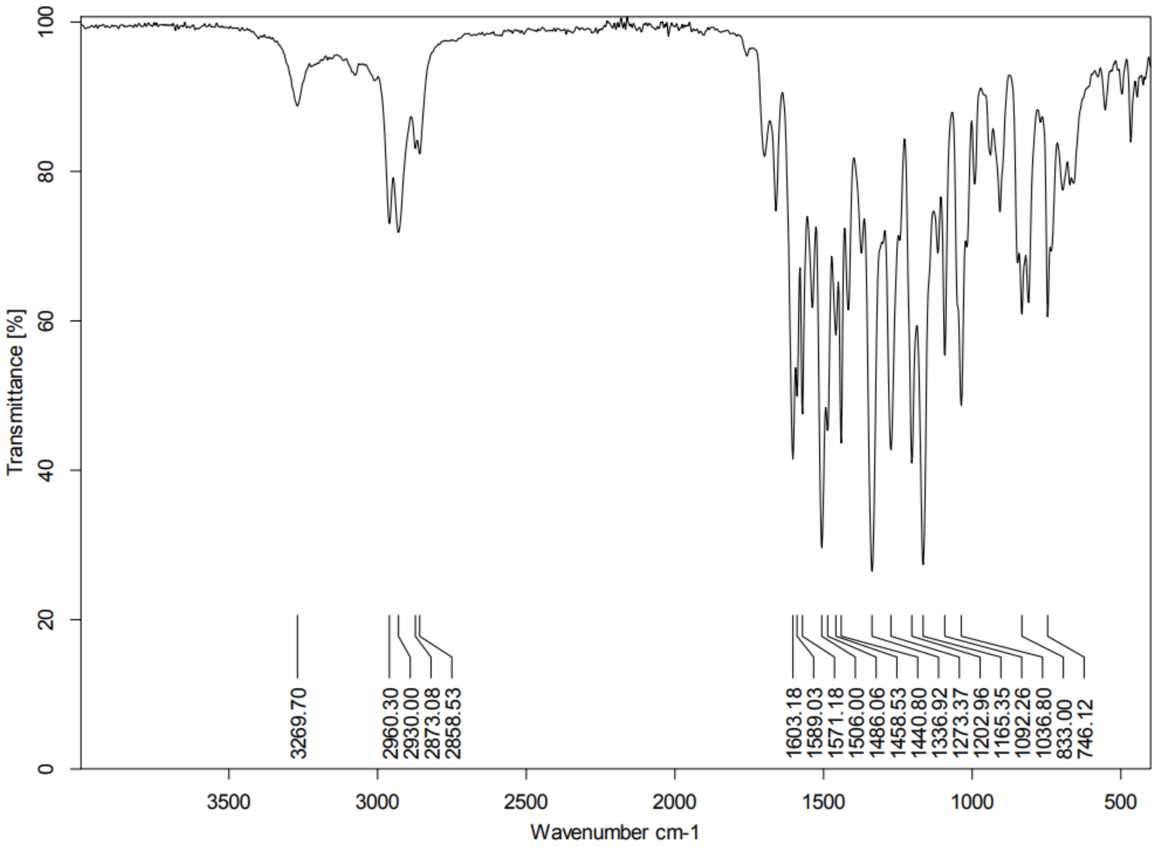

Figure S.44 – FT-IR spectrum of **14**

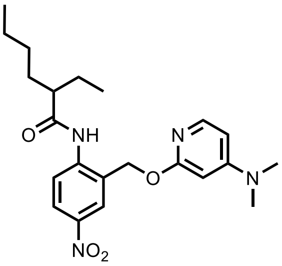

2-ethyl-N-(2-(((4-(dimethylamino)pyridin-2-yl)oxy)methyl)-4-nitrophenyl)hexanamide, **15**. 4-(dimethylamino)-2-((pyridin-2-yloxy)methyl)aniline (30.1 mg, 0.104 mmol) and NEt_3 (20 µL, 0.144 mmol) were dissolved in dry DCM (2 mL) under a N_2 atmosphere. 2-ethylhexanoyl chloride (20 µL, 0.115 mmol) was added and the reaction stirred overnight. The reaction mixture was diluted with EtOAc (50 mL), and then washed with 1M NaOH (25 mL), sat. $NaHCO_3$ (2 x 25 mL) and brine (25 mL). The organic layer was dried with $MgSO_4$ and in vacuo. The crude product was purified by flash chromatography (PE/EtOAc on SiO_2) to yield the desired product as a white solid (5.2 mg, 0.0125 mmol, 12%)

ν_{max} (film) cm^{-1} : 2958 (C-H), 2929 (C-H), 2859 (C-H), 1698 (C=O), 1613 (C=C), 1589 (C=C), 1539 (C=C), 1509 (C=C), 1489 (C=C), 1459 (C=C), 1446, 1384, 1271, 1159, 1034, 817

^{1}H NMR (400 MHz, $CDCl_3$) δ_H ppm: 1H NMR (400 MHz, Chloroform-d) δ 11.24 (s, 1H), 8.37 (d, J = 4.5 Hz, 1H), 8.35 (d, J = 1.8 Hz, 1H), 8.19 (dd, J = 9.2, 2.7 Hz, 1H), 7.81 (d, J = 6.3 Hz, 1H), 6.31 (dd, J = 6.3, 2.4 Hz, 1H), 5.91 (d, J = 2.3 Hz, 1H), 5.36 (d, J = 0.9 Hz, 2H), 2.99 (s, 6H), 2.38 (tt, J = 9.4, 5.1 Hz, 1H), 1.84 – 1.73 (m, 2H), 1.69-1.52 (m, 2H), 1.39-1.23 (m, 6H), 0.99 (t, J = 7.4 Hz, 3H), 0.92 – 0.83 (m, 3 H).

^{13}C{^{1}H} NMR (101MHz, $CDCl_3$) δ_C ppm: 13C NMR (101 MHz, CDCl3) 164.26, 157.53, 145.42, 143.61, 127.77, 125.03, 103.58, 91.44, 63.96, 50.78, 39.48, 32.58, 30.05, 26.33, 22.94, 14.13, 12.32.

HRMS: calc for $C_{22}H_{29}N_4O_4^+$ $[M-H]^-$: 413.2189, found: 413.2193

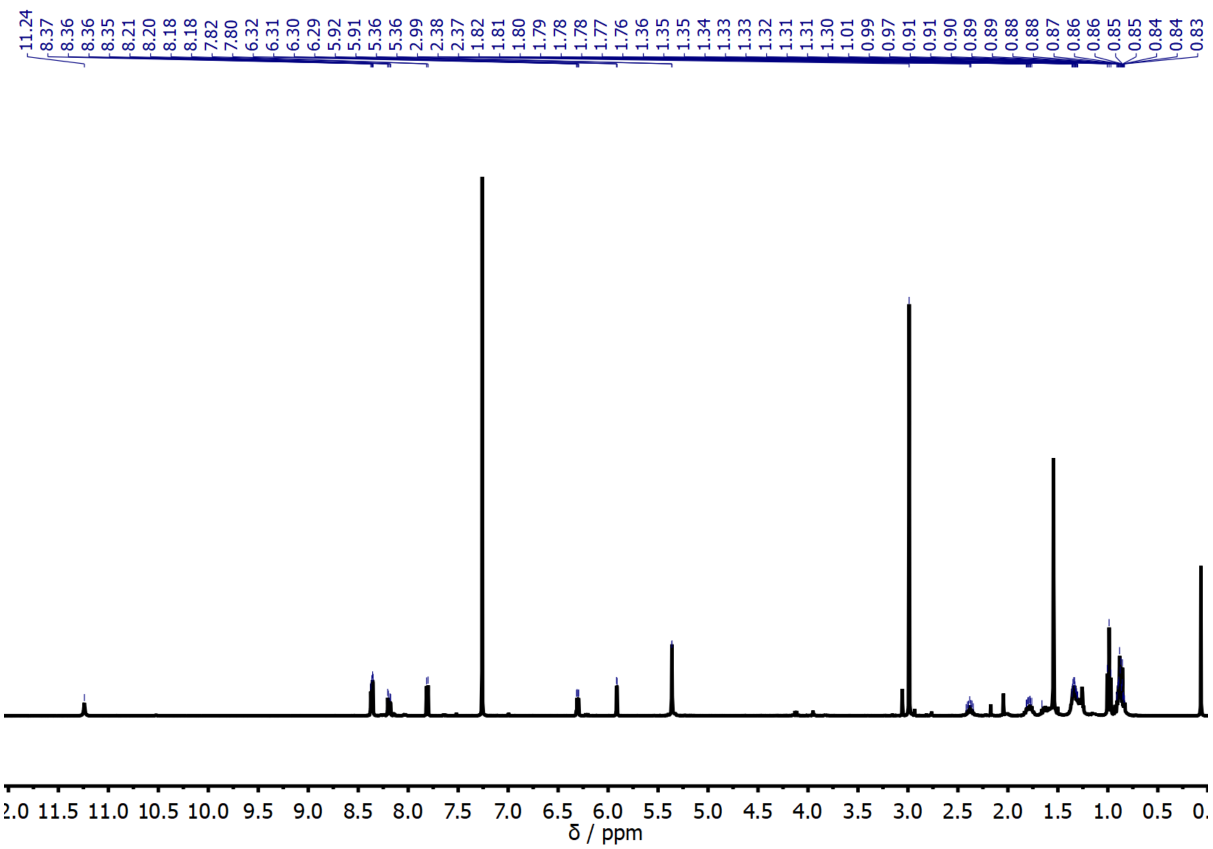

Figure S.45 – ^{1}H NMR spectrum of **15** in $CDCl_3$ (δ 0 – 12 ppm).

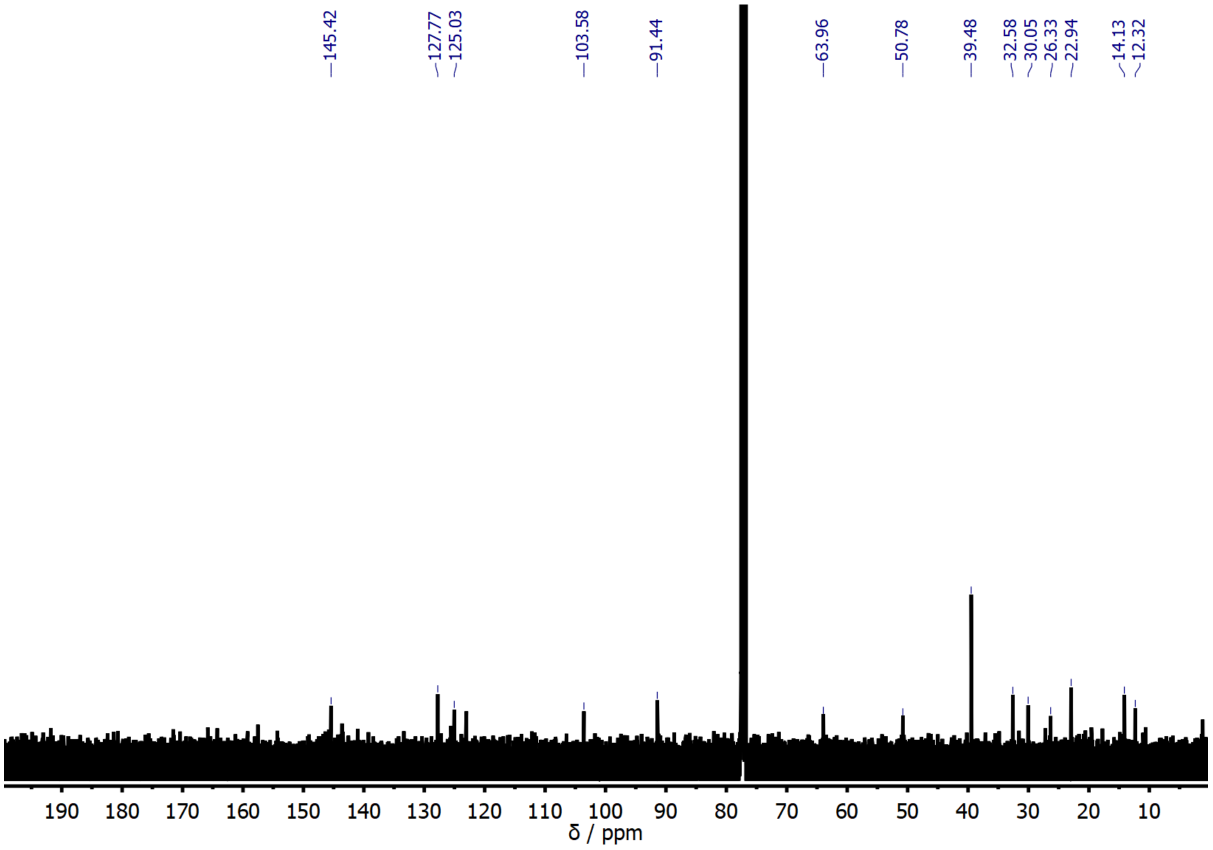

Figure S.46 – ^{13}C NMR spectrum of **15** in $CDCl_3$ (δ 0 – 200 ppm).

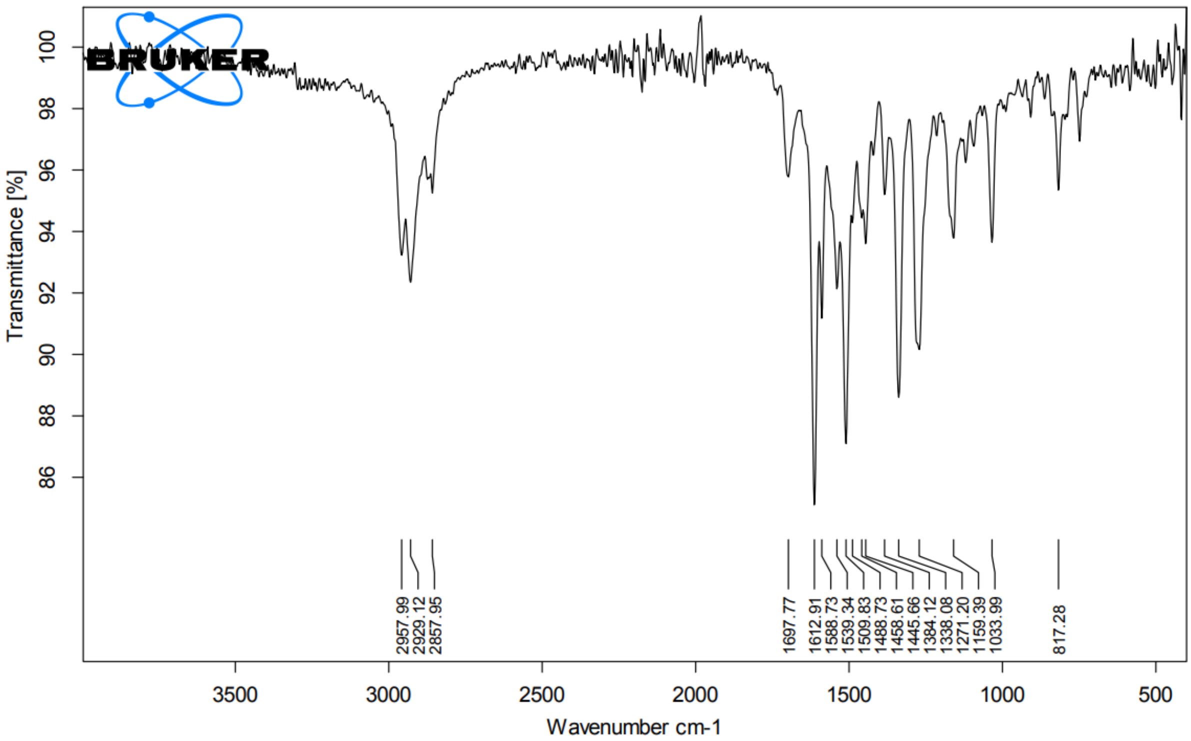

Figure S.47 – FT-IR spectrum of **15**

UV/Vis Absorption Spectroscopy and ^{1}H NMR Titrations

UV/Vis titrations were carried out on an Agilent Cary 60 UV-Vis spectrophotometer, using standard titration protocols. A 5 mL sample of the host (**1-11**) was prepared at a known concentration (typically between 0.020-0.070 mM) in *n*-octane. The UV/Vis spectrum of the free host (2 mL) was recorded. The guest was dissolved (PFTB) in 2 mL of the host solution. Aliquots of the guest solution were successively added to the cuvette, and the UV/Vis absorption spectrum was recorded after each addition. The UV/Vis absorption spectra were analysed using a purpose-built Python script to fit the changes in the absorption at fixed wavelengths to a 1:1 binding isotherm accounting for guest absorption, using Equation 1,[4].

$$\frac{A - A_0}{A_f - A_0} = \frac{[HG]}{[H]_0} = \frac{(K([H]_0 + [G]_0) + 1) \pm \sqrt{(K([H]_0 + [G]_0) + 1)^2 - 4K^2[H]_0[G]_0}}{2K[H]_0}$$

Equation 1

A	Absorbance
A_0	Initial absorbance
A_f	Final absorbance
[HG]	Host-guest complex concentration
$[H]_0$	Total host concentration
$[G]_0$	Total guest concentration
K	Association constant

Table S.2 – Definitions of the terms in Equation 2.

NMR titrations were carried out on a Bruker 400 MHz spectrometer using WET solvent suppression. A 2 mL sample of the host was prepared at a concentration of 0.18-0.22 mM in *n*-octane. The NMR spectrum of the host solution (0.6 mL) was recorded. The guest PFTB solution was prepared at a concentration of using the host solution as solvent. Aliquots of the guest solution were successively added to the NMR sample tube containing the host solution, and the ^{1}H NMR spectrum was recorded after each addition. For the dilution experiment, 0.6 mL of octane was placed in an NMR tube and aliquots of a solution of in octane was added with the spectrum being recorded after every addition. The NMR spectra were analysed using a purpose-built Python script to fit the changes in the chemical shifts for different protons to a 1:1 binding isotherm, using Equation 2[4], or a 1:2 binding isotherm.

$$\frac{\delta_{obs} - \delta_H}{\delta_{HG} - \delta_H} = \frac{[HG]}{[H]_0} = \frac{(K([H]_0 + [G]_0) + 1) \pm \sqrt{(K([H]_0 + [G]_0) + 1)^2 - 4K^2[H]_0[G]_0}}{2K[H]_0}$$

Equation 2

δ_{obs}	Observed chemical shift
δ_H	Host chemical shift
δ_{HG}	Complex chemical shift
[HG]	Host-guest complex concentration
$[H]_0$	Total host concentration
$[G]_0$	Total guest concentration
K	Association constant

Table S.3 – Definitions of the terms in Equation 1.

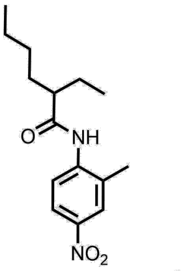

Figure S.48 – Structure of the host **1**

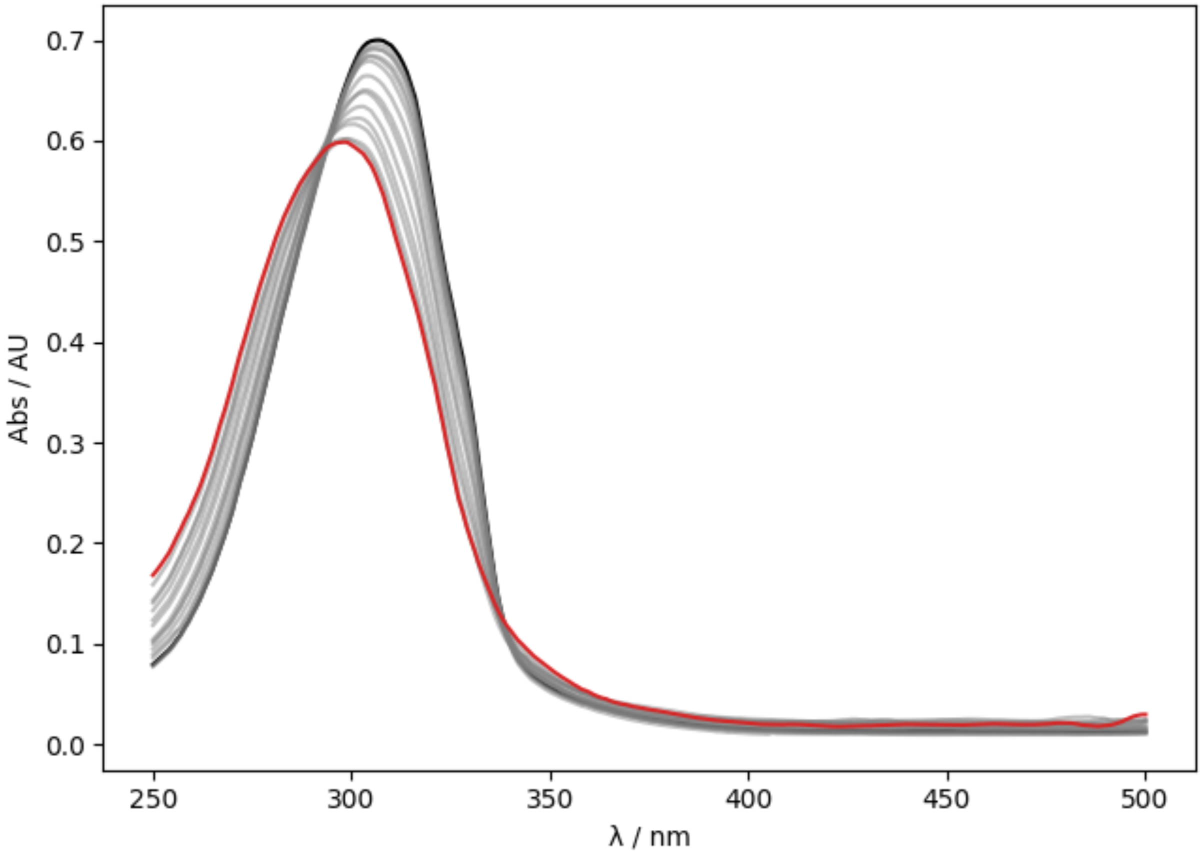

Figure S.49 - UV/Vis absorption spectra for the titration of PFTB into **1** (0.0401 mM in *n*-octane, at 298K). The UV/Vis spectrum of the host **1** and the final point of the titration are reported in black and in red, respectively.

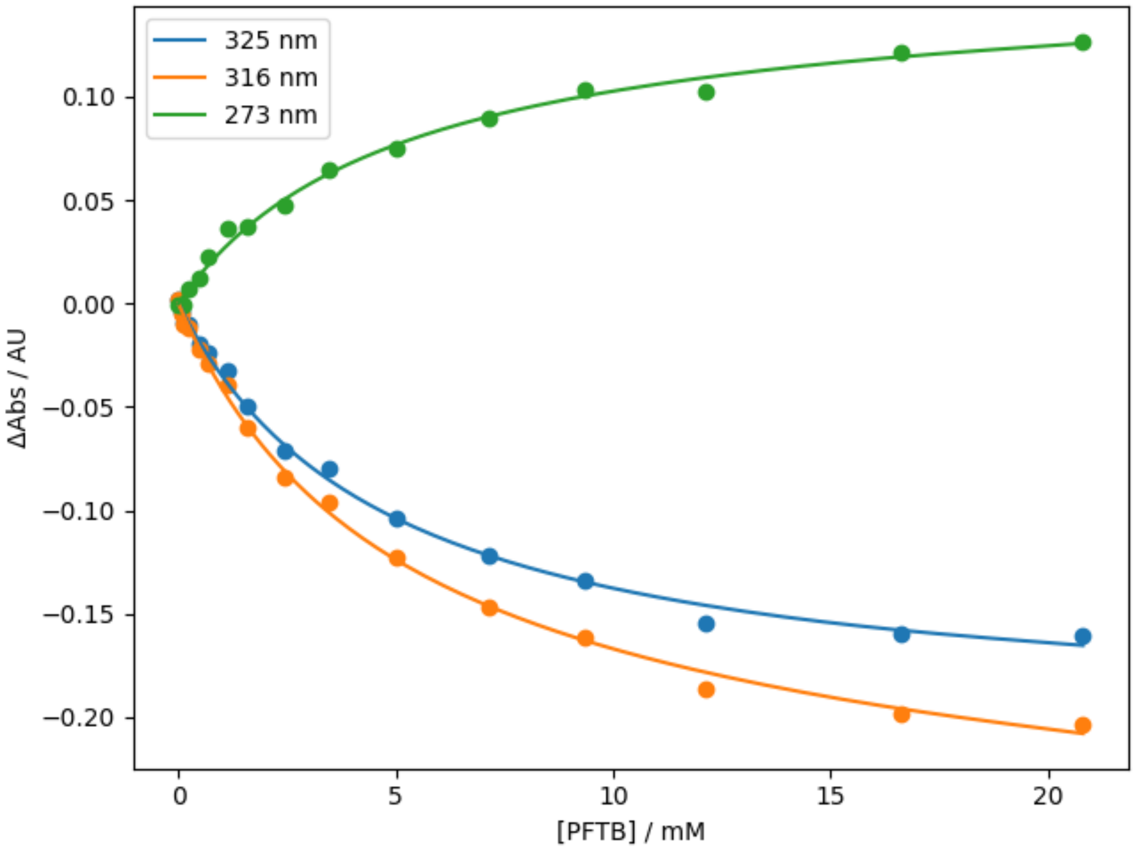

Figure S.50 - The fit of the absorbance at selected wavelengths to a 1:1 binding isotherm accounting for guest absorption for the titration of PFTB into **1** (0.0401 mM in *n*-octane, at 298 K).

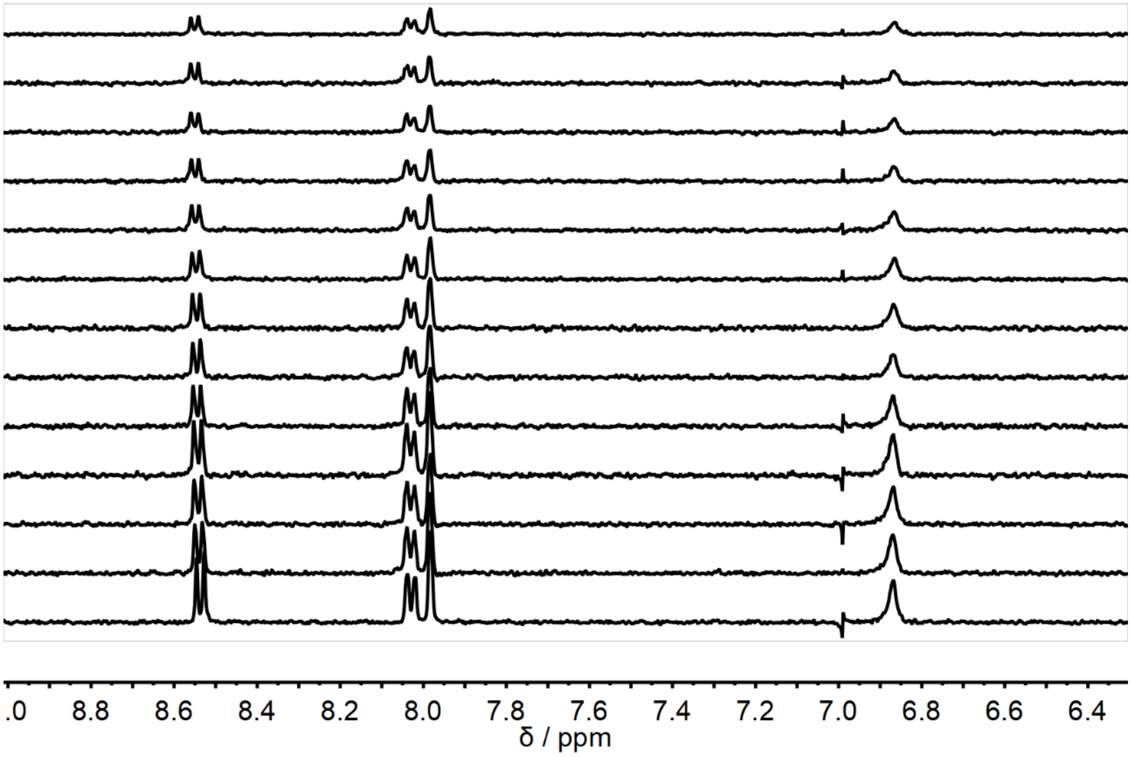

Figure S.51 – Stack plot for the NMR dilution of **1** in *n*-octane ranging from 0.267 mM (bottom) to 0.0501 mM (top)

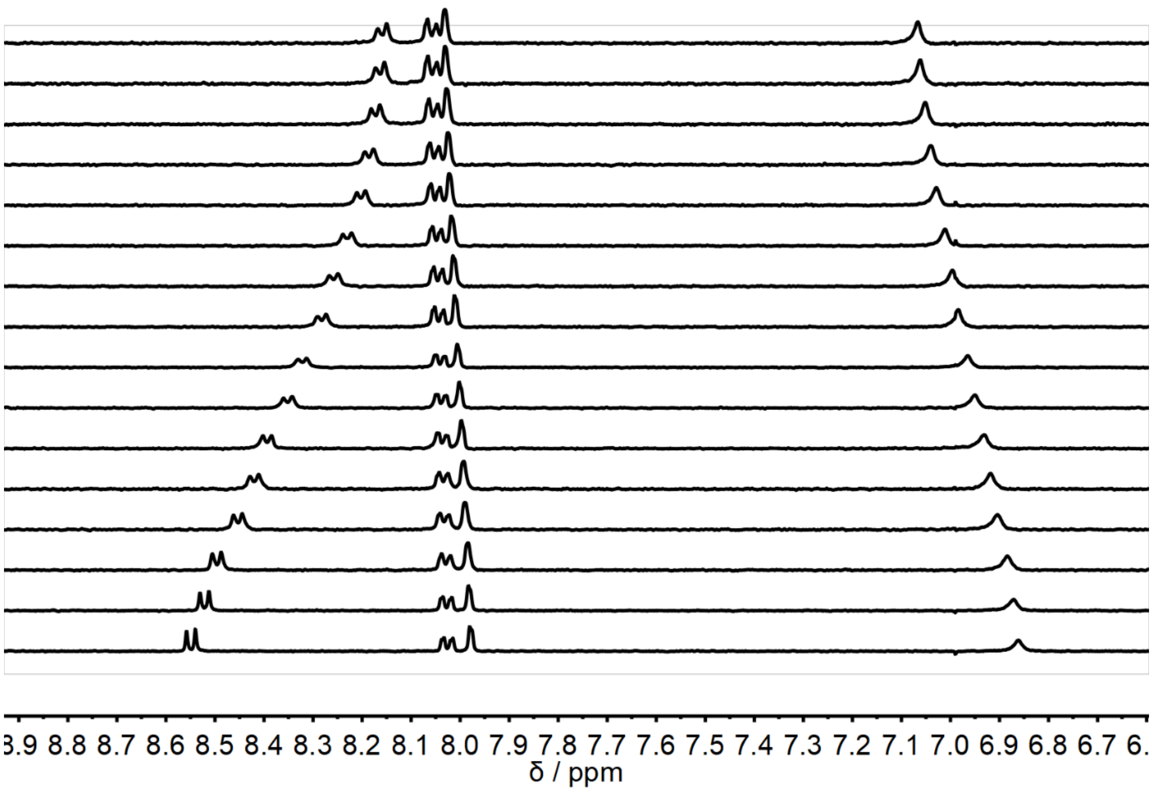

Figure S.52 – Stack plot for the NMR titration of PFTB into **1** (0.267 mM) in *n*-octane at 298 K.

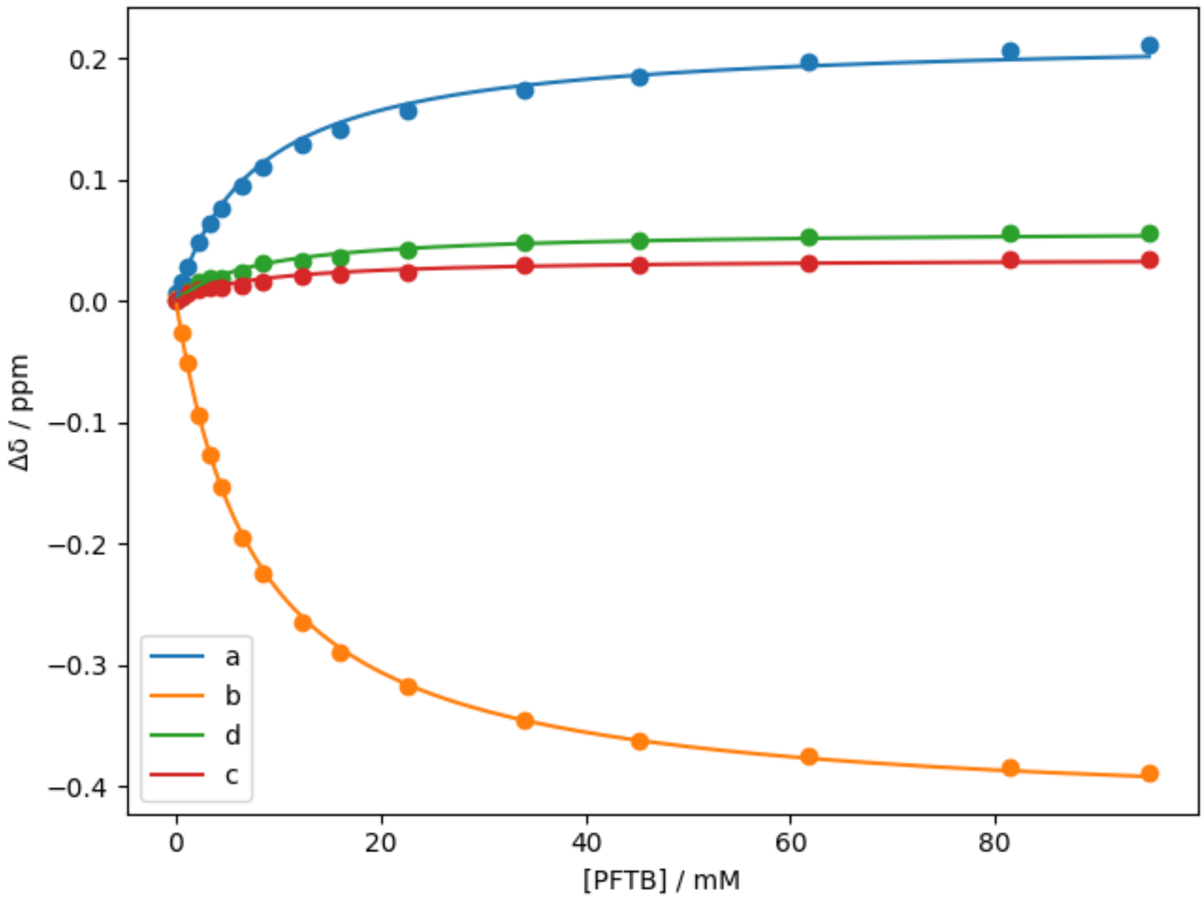

Figure S.53 – Fit for the NMR titration to a 1:1 binding isotherm of PFTB into **1** (0.267 mM) in *n*-octane at 298K.

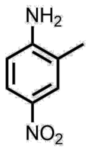

Figure S.54 – Structure of the host 2-methyl-4-nitroaniline

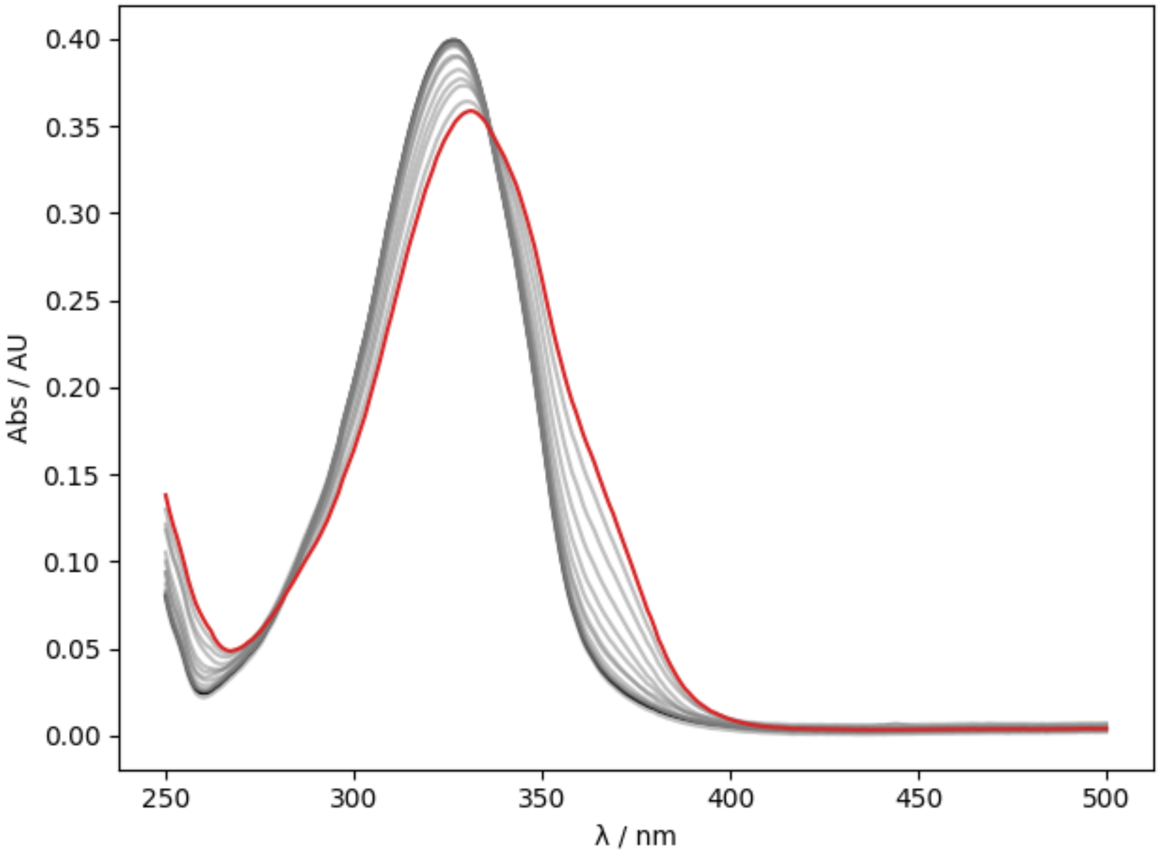

Figure S.55 - UV/Vis absorption spectra for the titration of PFTB into 2-methyl-4-nitroaniline (0.0414 mM in *n*-octane, at 298K). The UV/Vis spectrum of the host 2-methyl-4-nitroaniline and the final point of the titration are reported in black and in red, respectively.

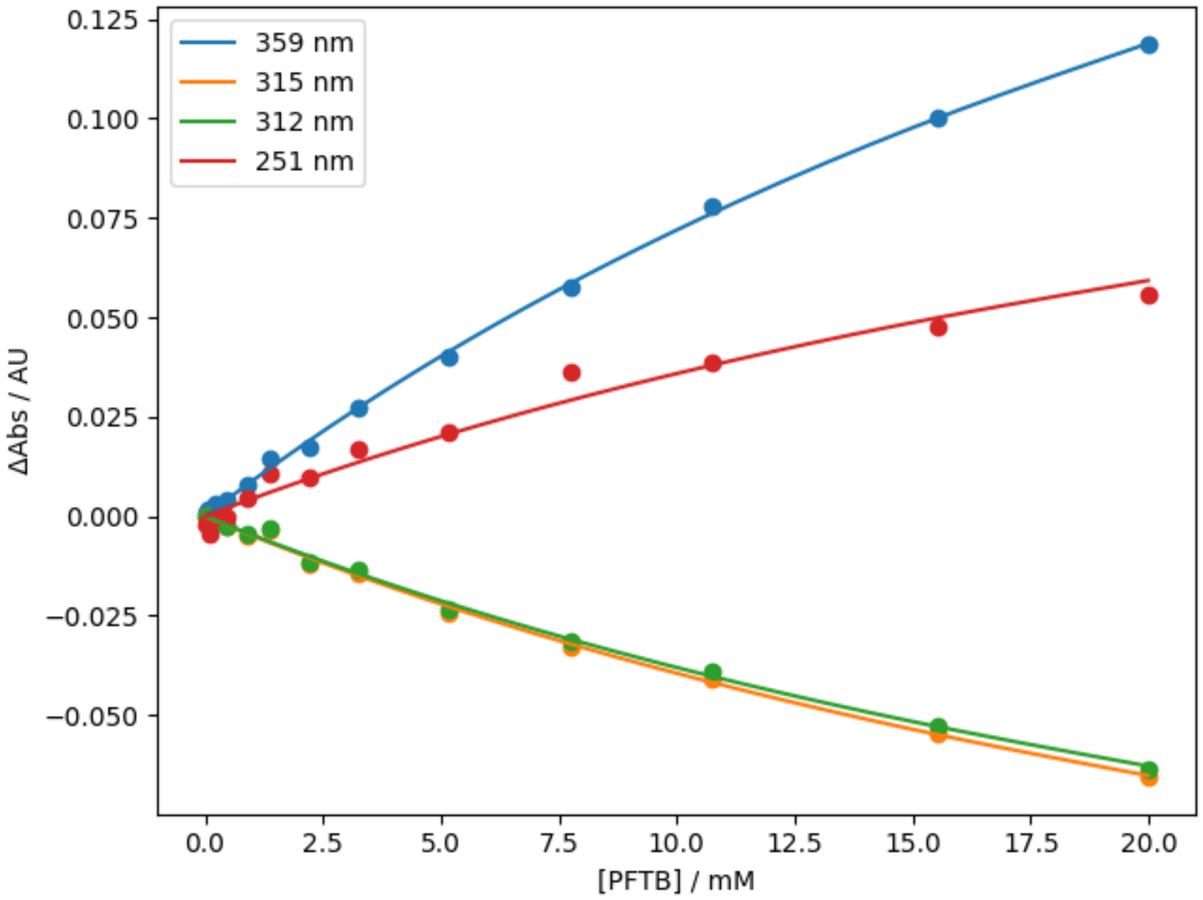

Figure S.56 - The fit of the selected absorbances to a 1:1 binding isotherm for the titration of PFTB into 2-methyl-4-nitroaniline (0.0414 mM in *n*-octane, at 298 K).

Deconvoluted UV/Vis spectra

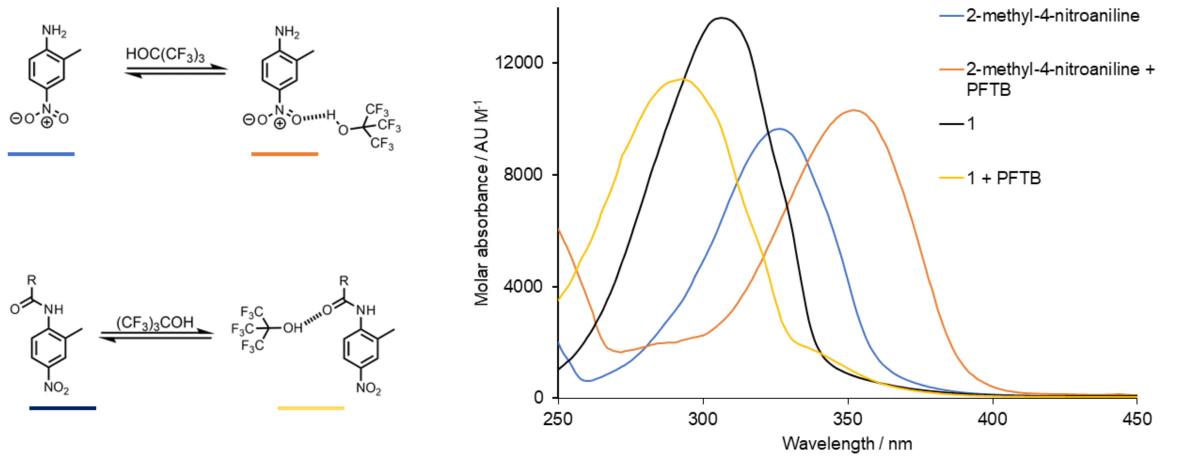

Figure S.57 - Deconvoluted spectra of free **1**, **1** bound to PFTB, free 2-methyl-4-nitroaniline, and 2-methyl-4-nitroaniline bound to PFTB

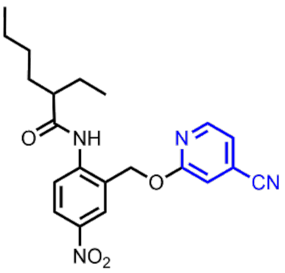

Figure S.58 – Structure of the host **10**

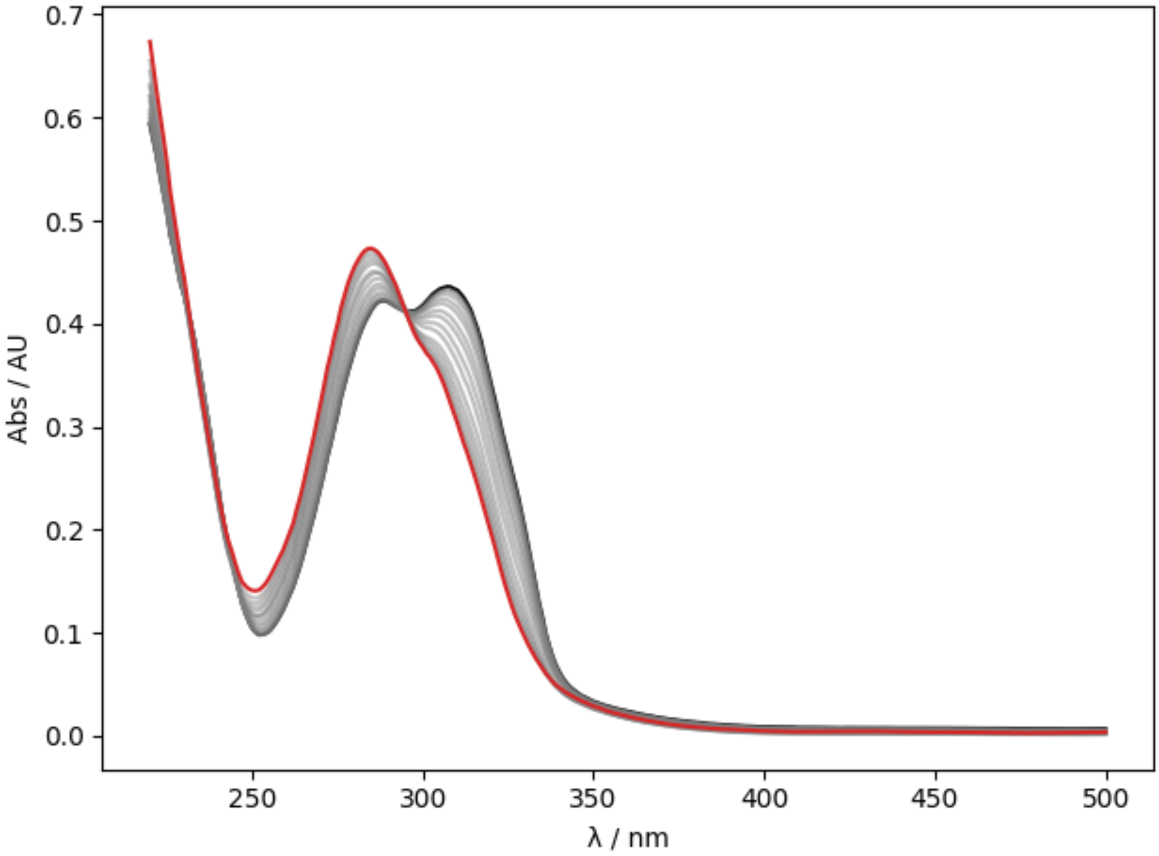

Figure S.59 - UV/Vis absorption spectra for the titration of PFTB into **11** (0.0314 mM in *n*-octane, at 298K). The UV/Vis spectrum of the host **11** and the final point of the titration are reported in black and in red, respectively.

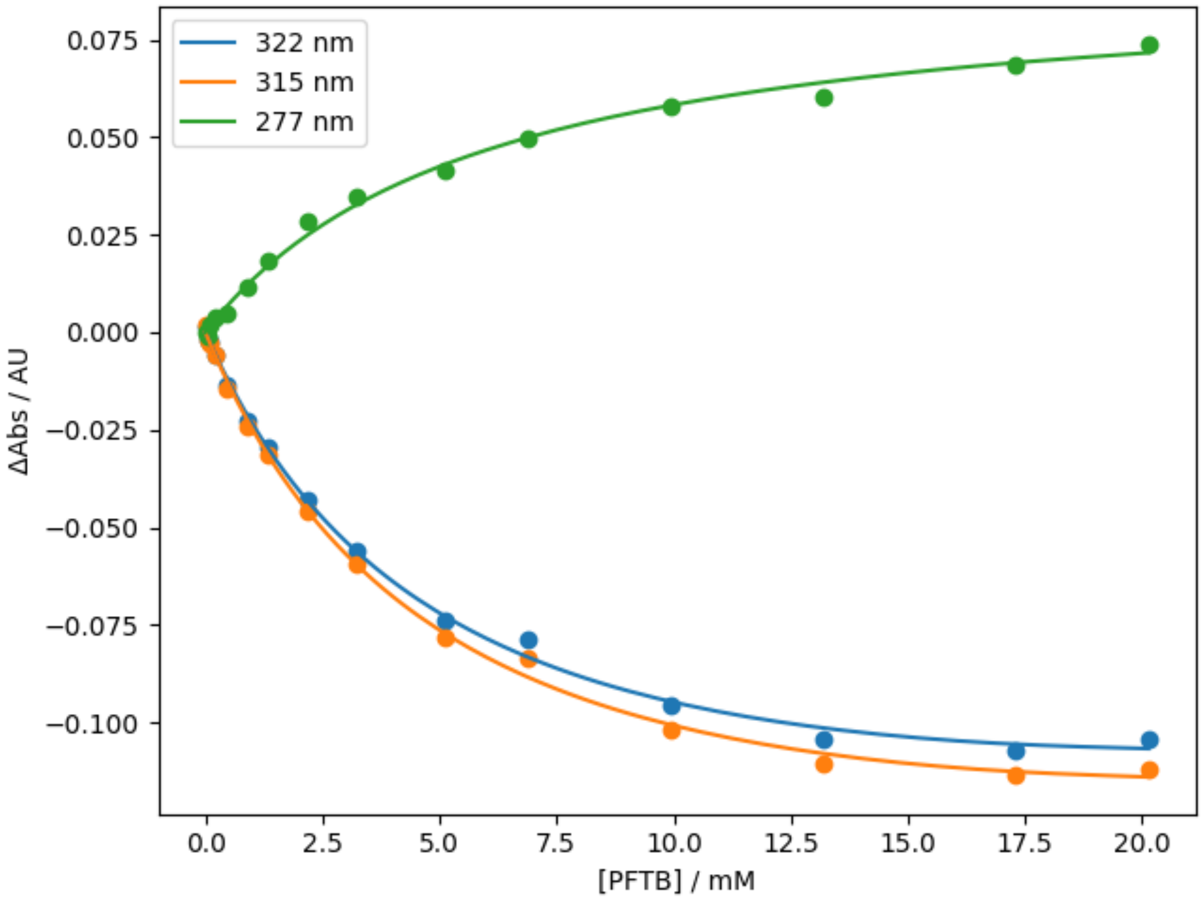

Figure S.60 - The fit of the selected absorbances to a 1:1 binding isotherm accounting for guest absorption for the titration of PFTB into **11** (0.0314 mM in *n*-octane, at 298 K).

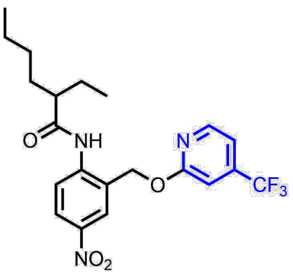

Figure S.61 – Structure of the host **11**

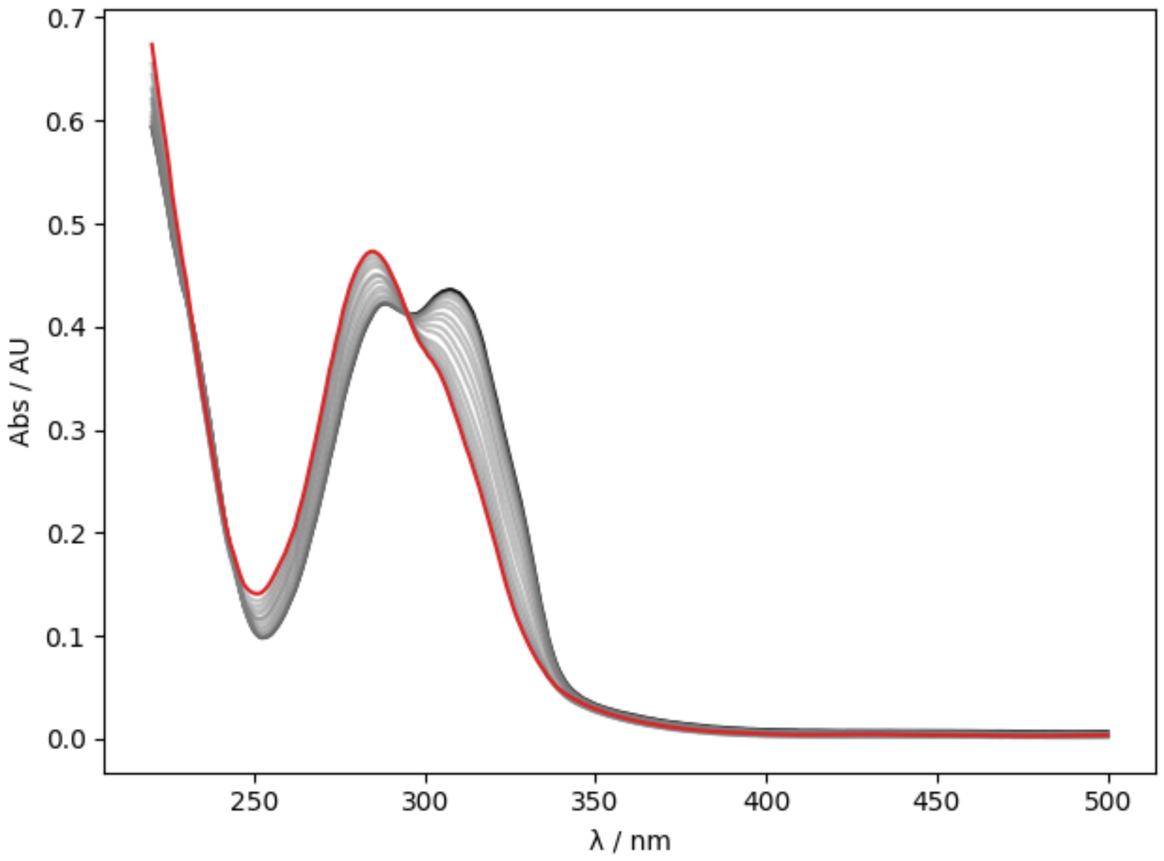

Figure S.62 - UV/Vis absorption spectra for the titration of PFTB into **11** (0.0314 mM in *n*-octane, at 298K). The UV/Vis spectrum of the host **11** and the final point of the titration are reported in black and in red, respectively.

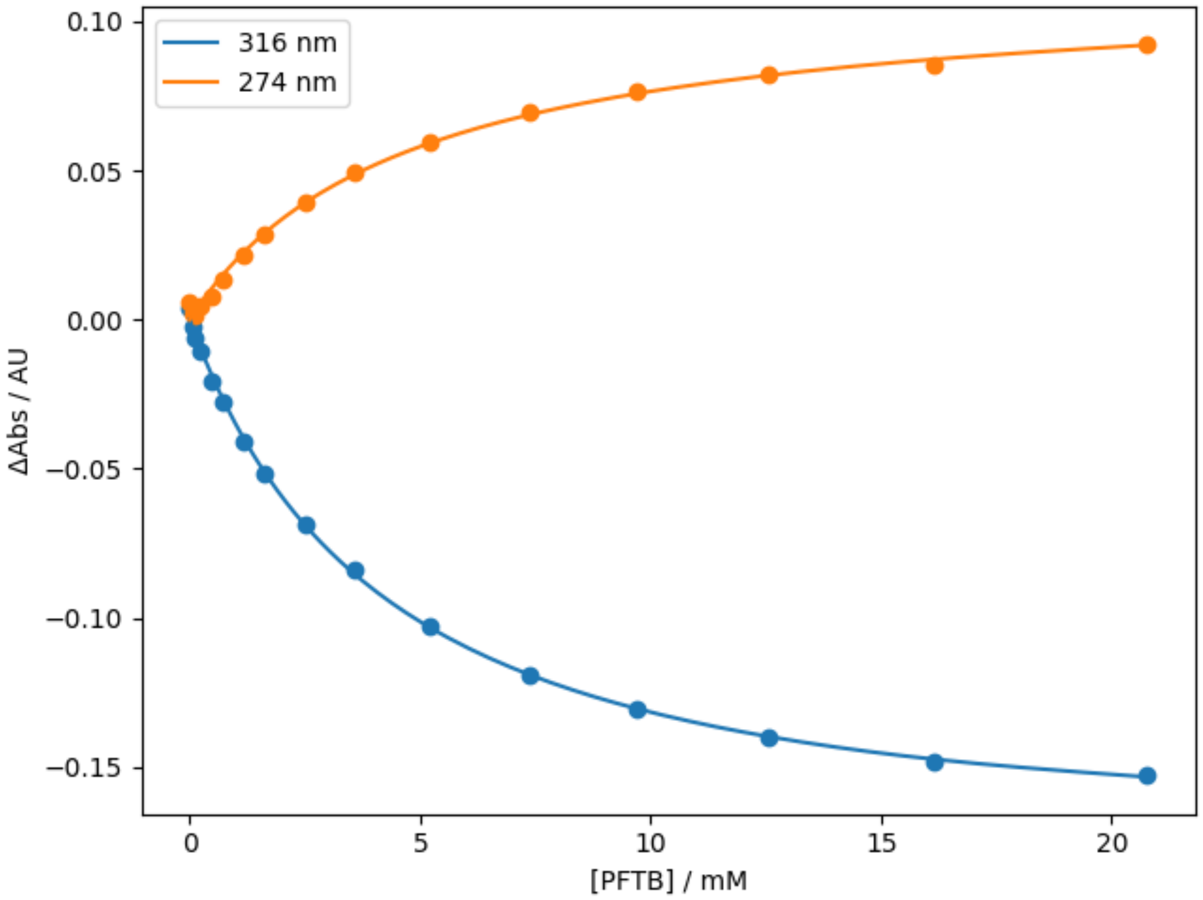

Figure S.63 - The fit of the selected absorbances to a 1:1 binding isotherm accounting for guest absorption for the titration of PFTB into **11** (0.0314 mM in *n*-octane, at 298 K).

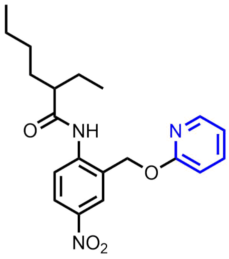

Figure S.64 –Structure of the host **12**.

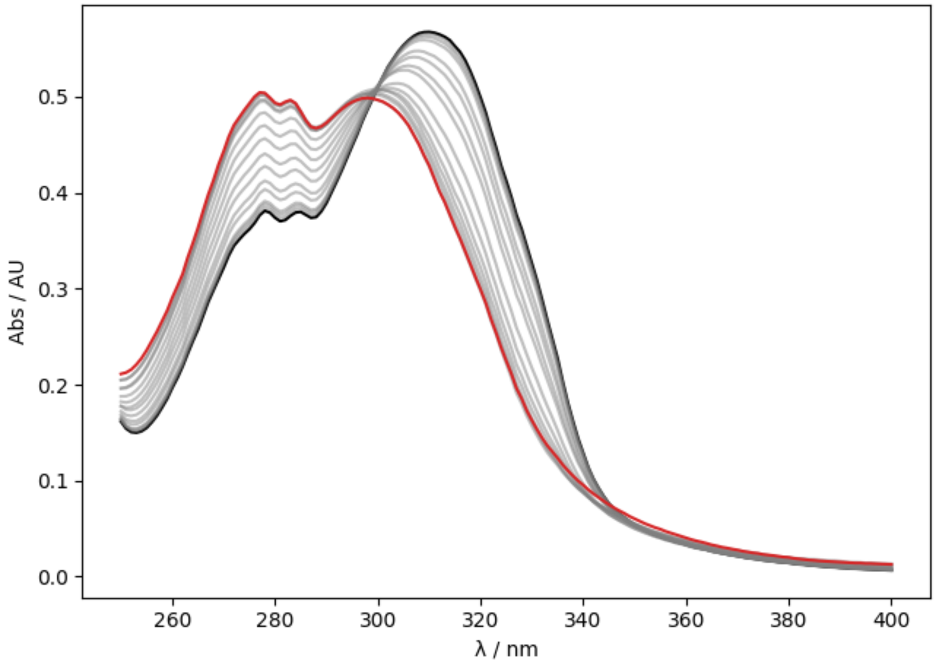

Figure S.66 - UV/Vis absorption spectra for the titration of HFIP into **13** (0.0373 mM in *n*-octane, at 298K). The UV/Vis spectrum of the host **12** and the final point of the titration are reported in black and in red, respectively.

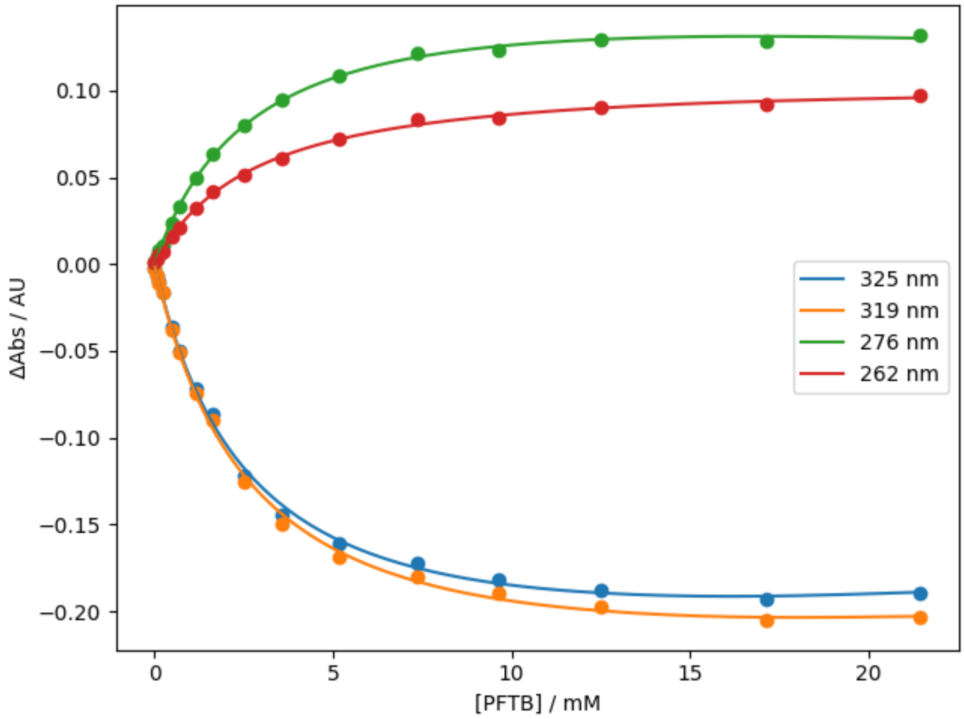

Figure S.67 - The fit of the absorbance at 278 nm (black) and 323 nm (red) to a 1:1 binding isotherm accounting for guest absorption for the titration of HFIP into **12** (0.0373 mM in *n*-octane, at 298 K).

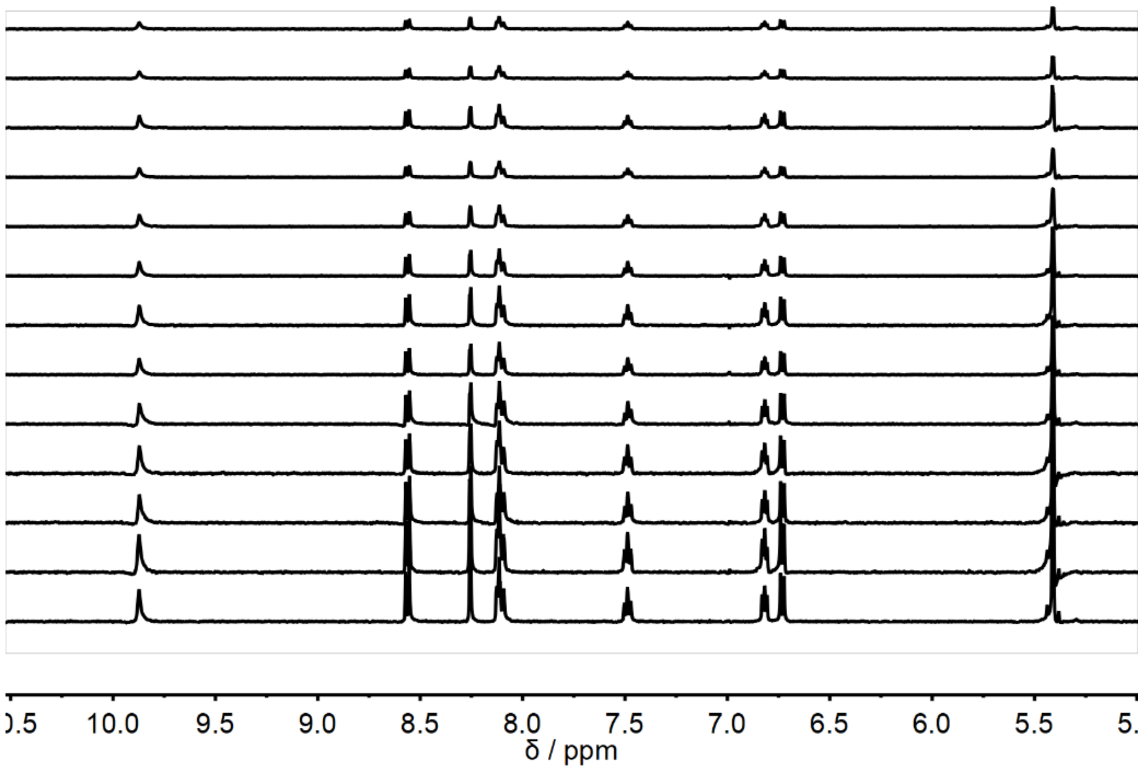

Figure S.68 – Stack plot for the NMR dilution of **12** in *n*-octane with concentration ranging from 0.695 mM (bottom) to 0.130 mM (top)

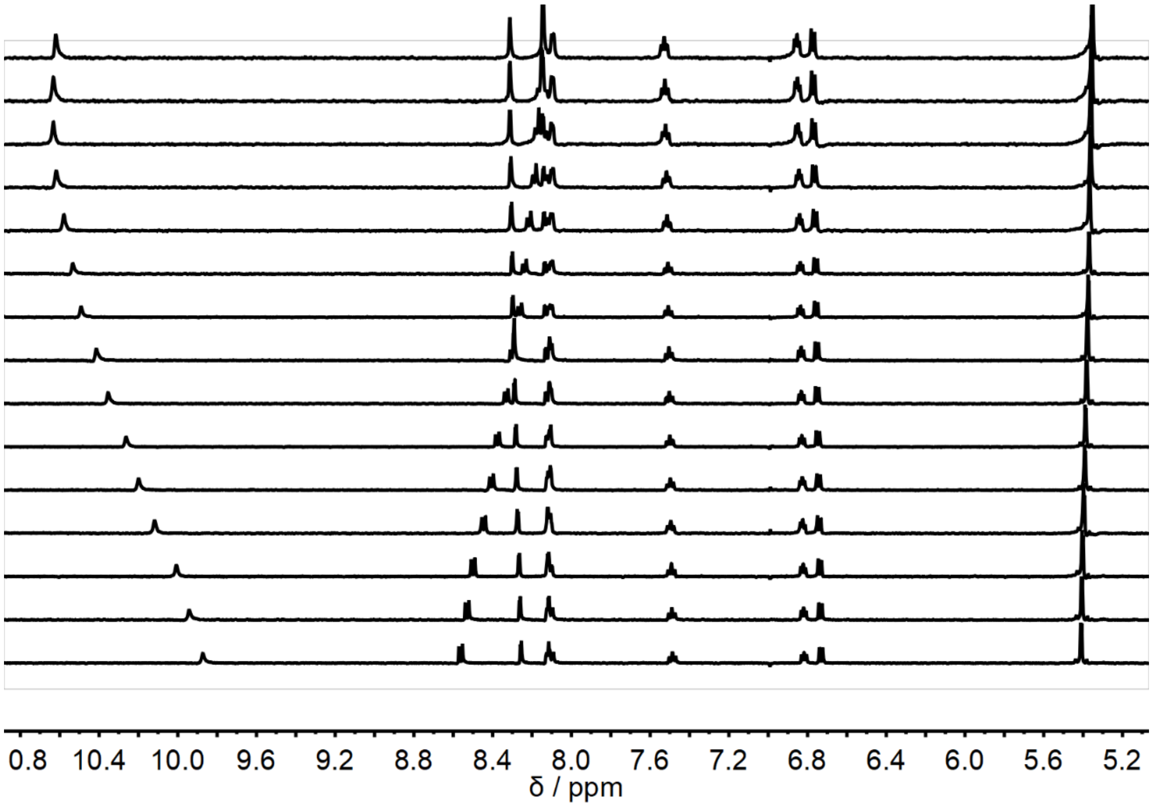

Figure S.69 – Stack plot for the NMR titration of PFTB into **12** (0.208 mM) in *n*-octane at 298 K.

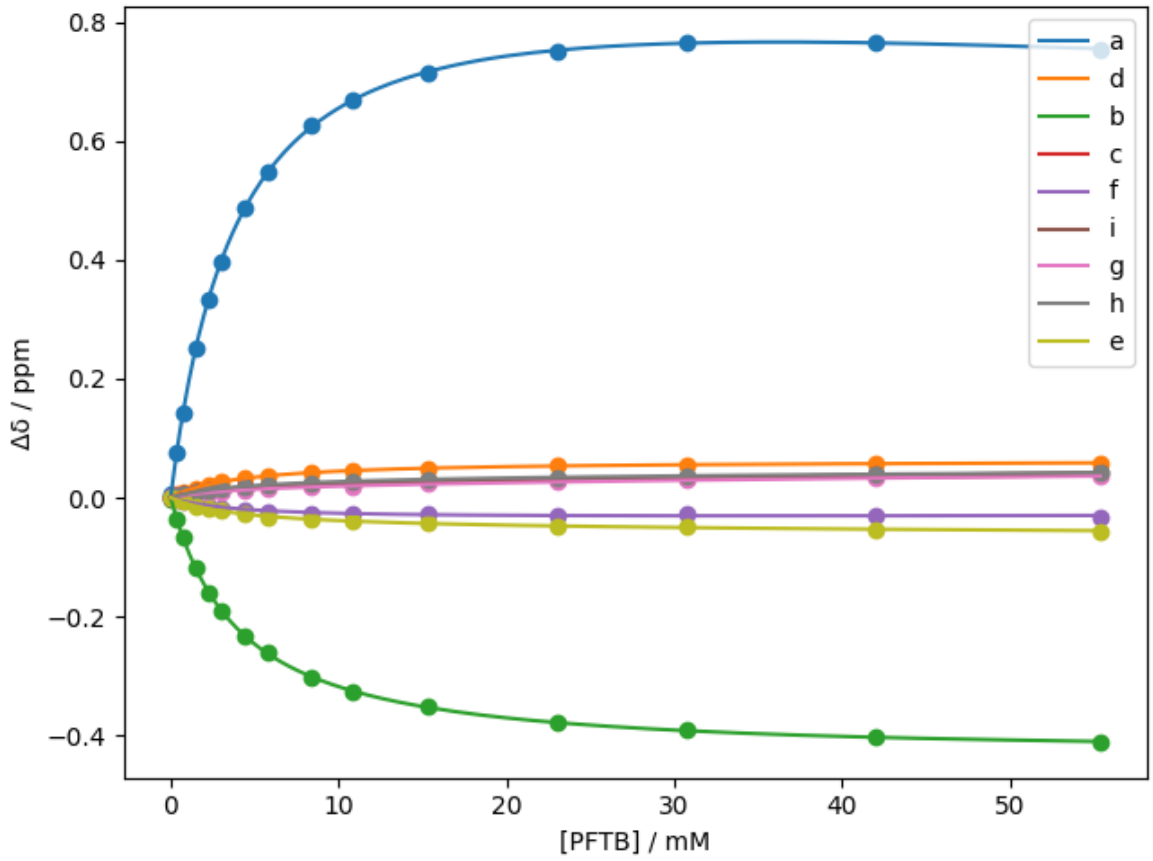

Figure S.70 – Fit for the NMR titration of PFTB into **12** (0.208 mM) to a 1:2 titration in *n*-octane at 298 K.

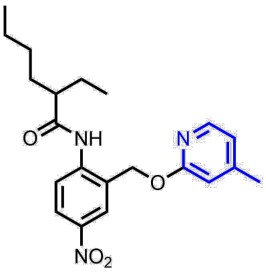

Figure S.71 – Structure of the host **13**

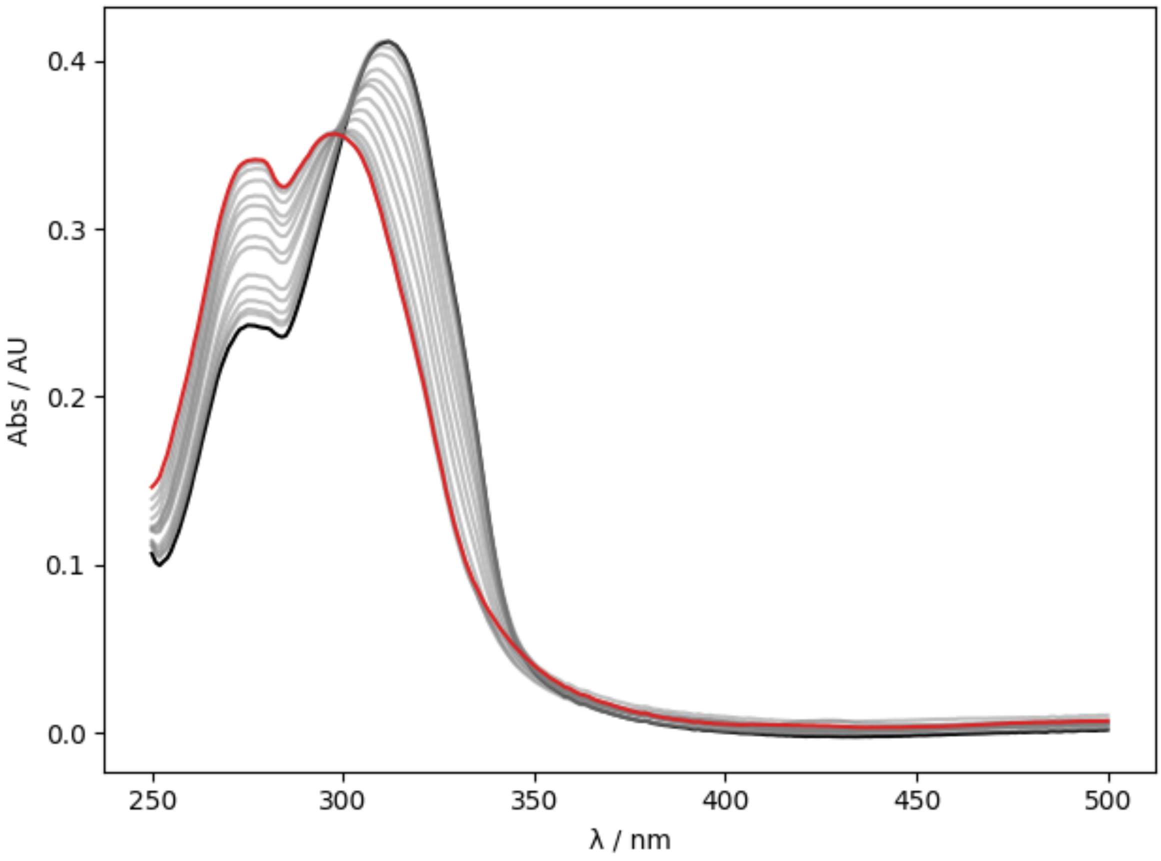

Figure S.72 - UV/Vis absorption spectra for the titration of PFTB into **25** (0.0286 mM in *n*-octane, at 298K). The UV/Vis spectrum of the host **13** and the final point of the titration are reported in black and in red, respectively.

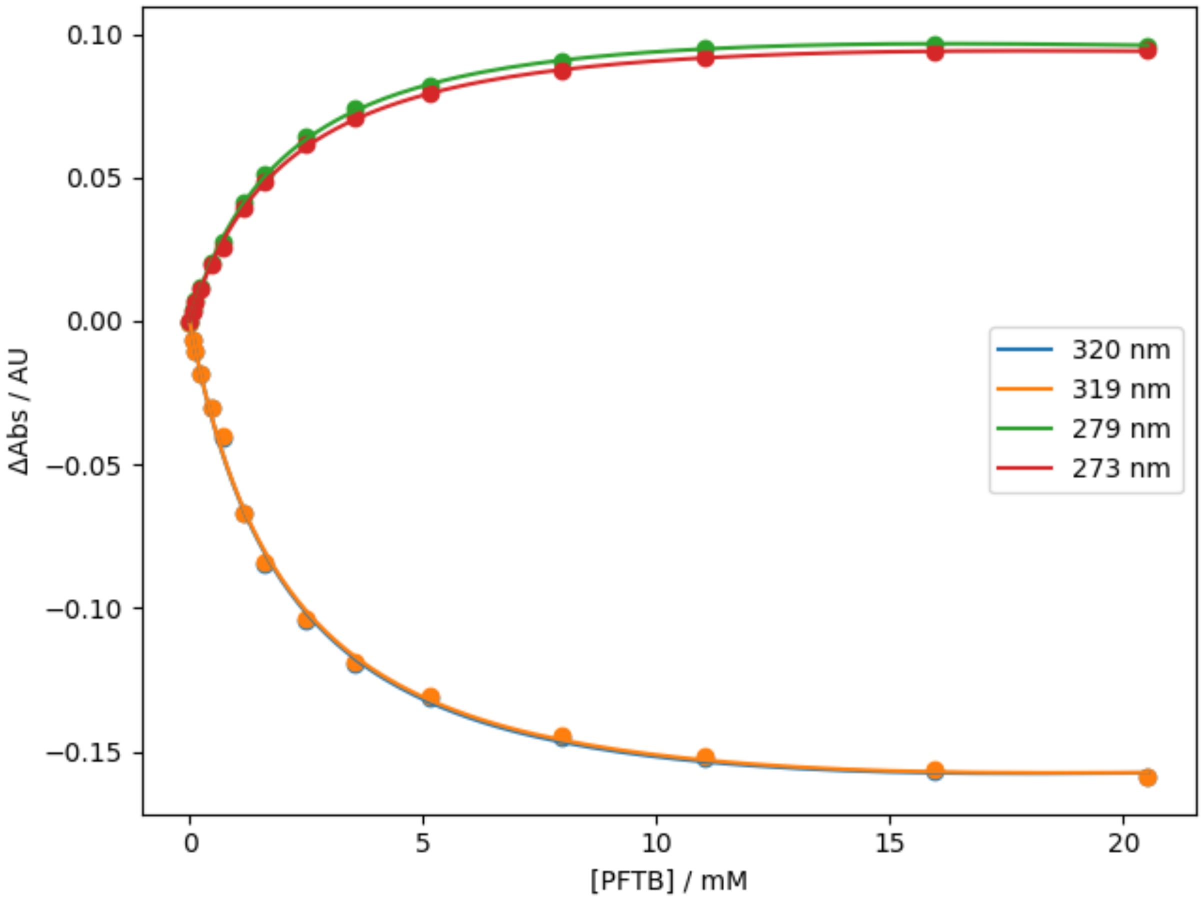

Figure S.73 - The fit of the selected absorbances to a 1:1 binding isotherm accounting for guest absorption for the titration of PFTB into **13** (0.0286 mM in *n*-octane, at 298 K).

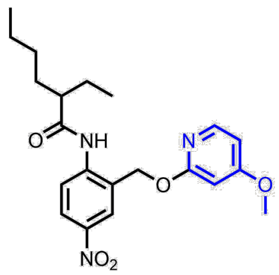

Figure S.74 – Structure of the host **14**

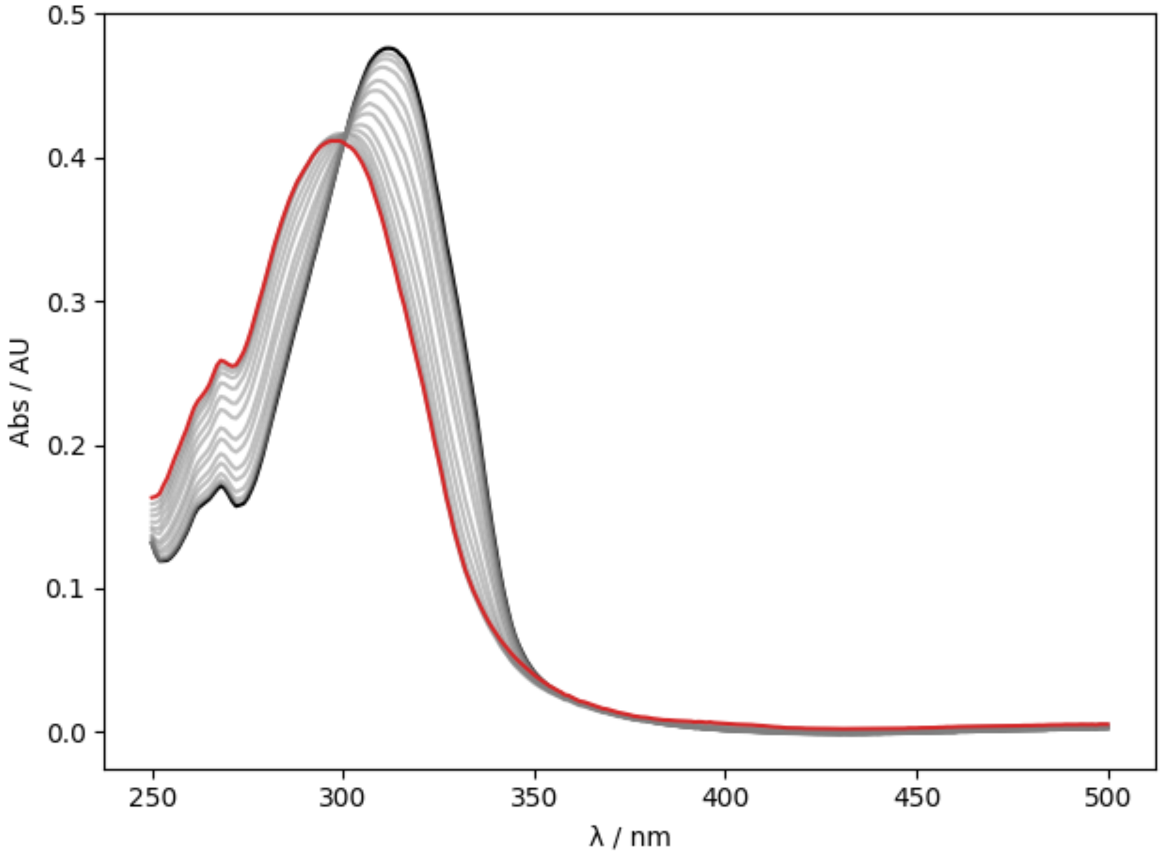

Figure S.75 - UV/Vis absorption spectra for the titration of PFTB into **14** (0.0350 mM in *n*-octane, at 298K). The UV/Vis spectrum of the host **14** and the final point of the titration are reported in black and in red, respectively.

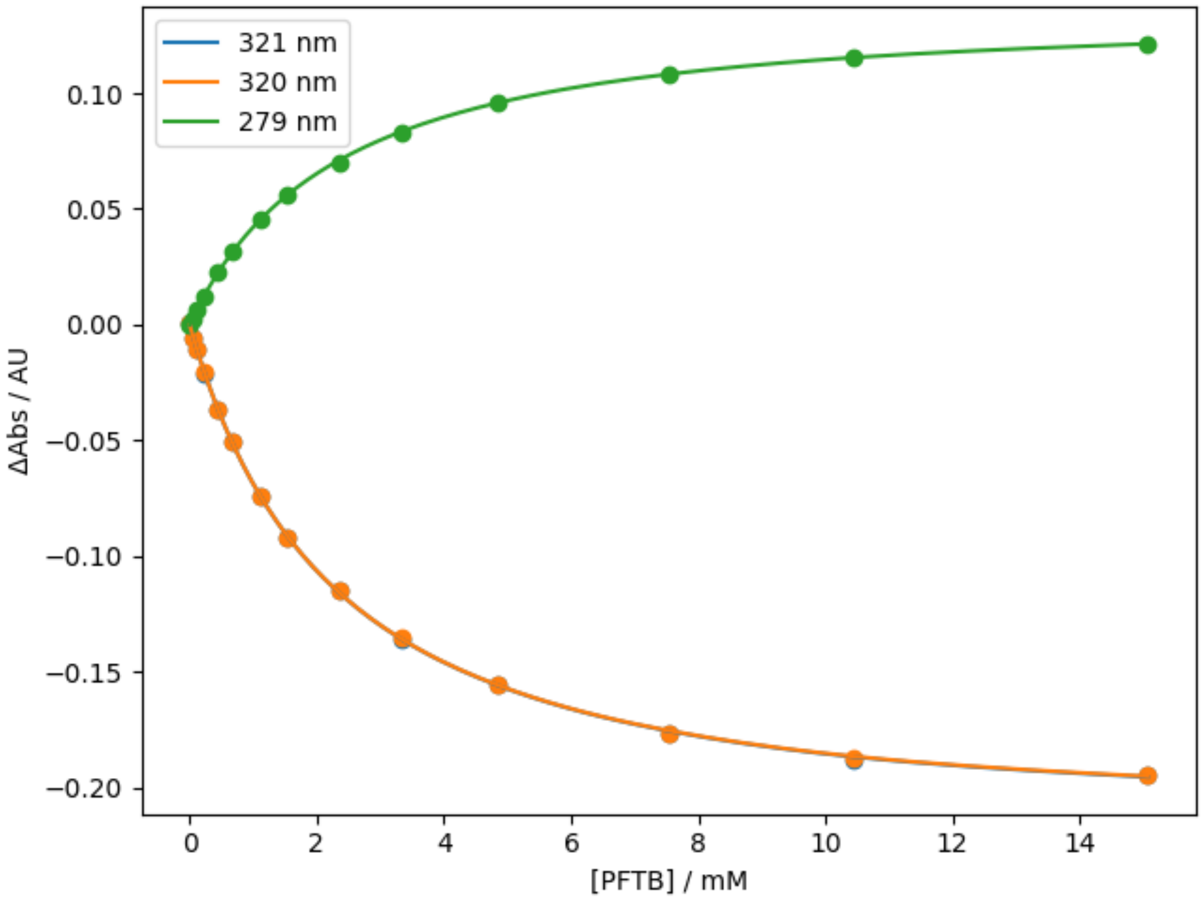

Figure S.76 - The fit of the selected absorbances to a 1:1 binding isotherm accounting for guest absorption for the titration of PFTB into **14** (0.0350 mM in *n*-octane, at 298 K).

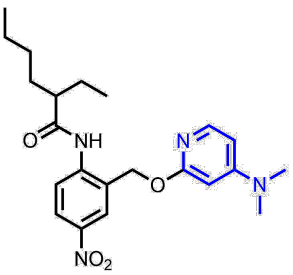

Figure S.77 – Structure of the host **15**

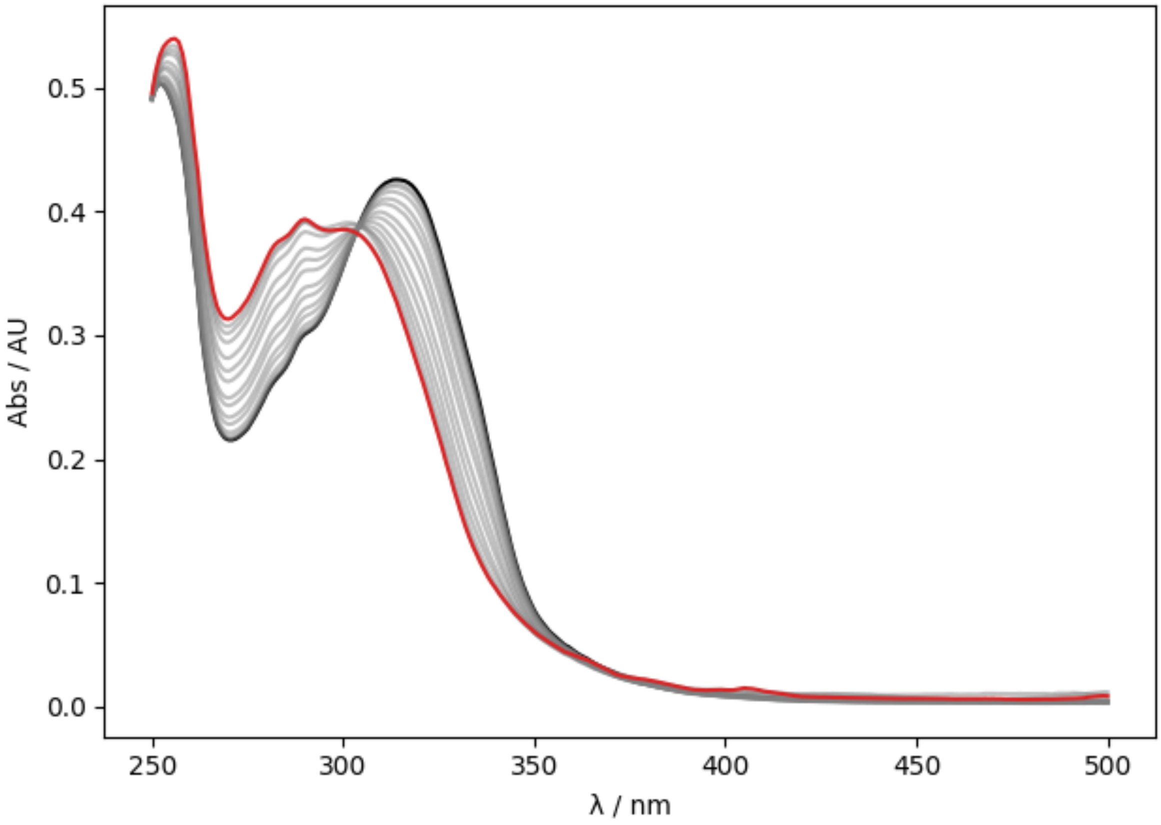

Figure S.78 - UV/Vis absorption spectra for the titration of PFTB into **15** (0.0323 mM in *n*-octane, at 298K). The UV/Vis spectrum of the host **15** and the final point of the titration are reported in black and in red, respectively.

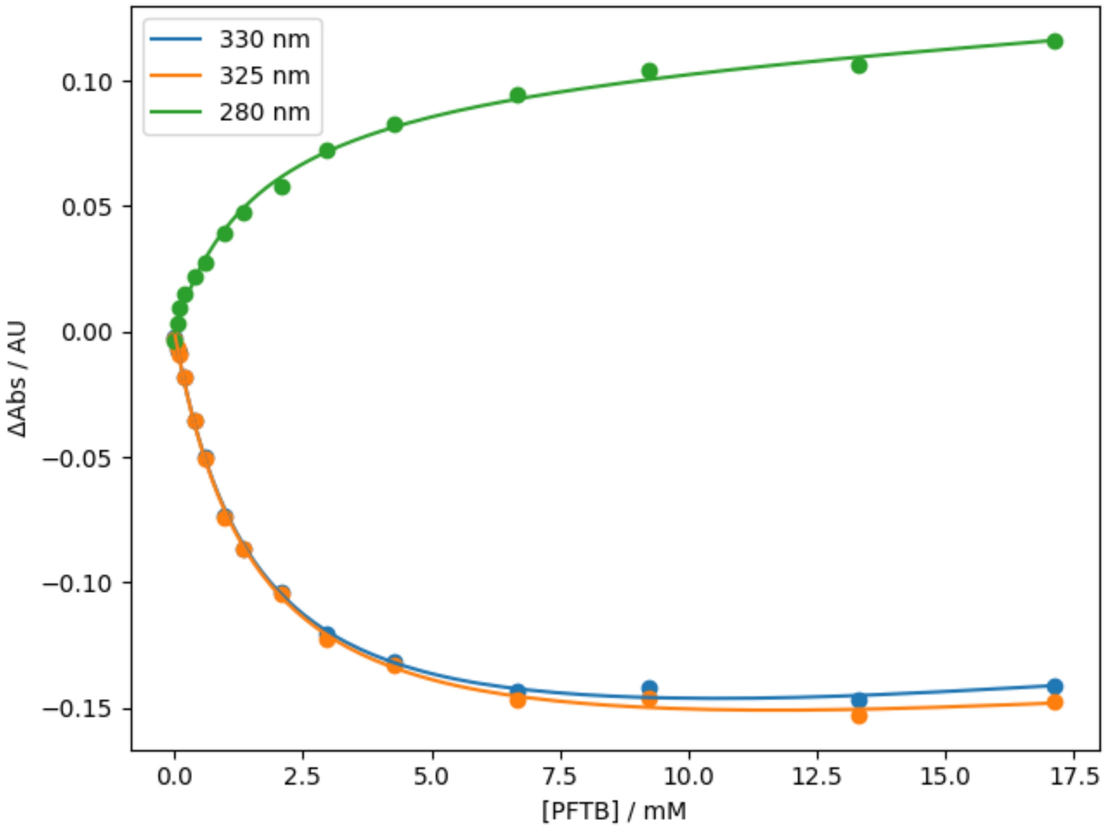

Figure S.79 - The fit of the selected absorbances to a 1:1 binding isotherm accounting for guest absorption for the titration of PFTB into **15** (0.0323 mM in *n*-octane, at 298 K).

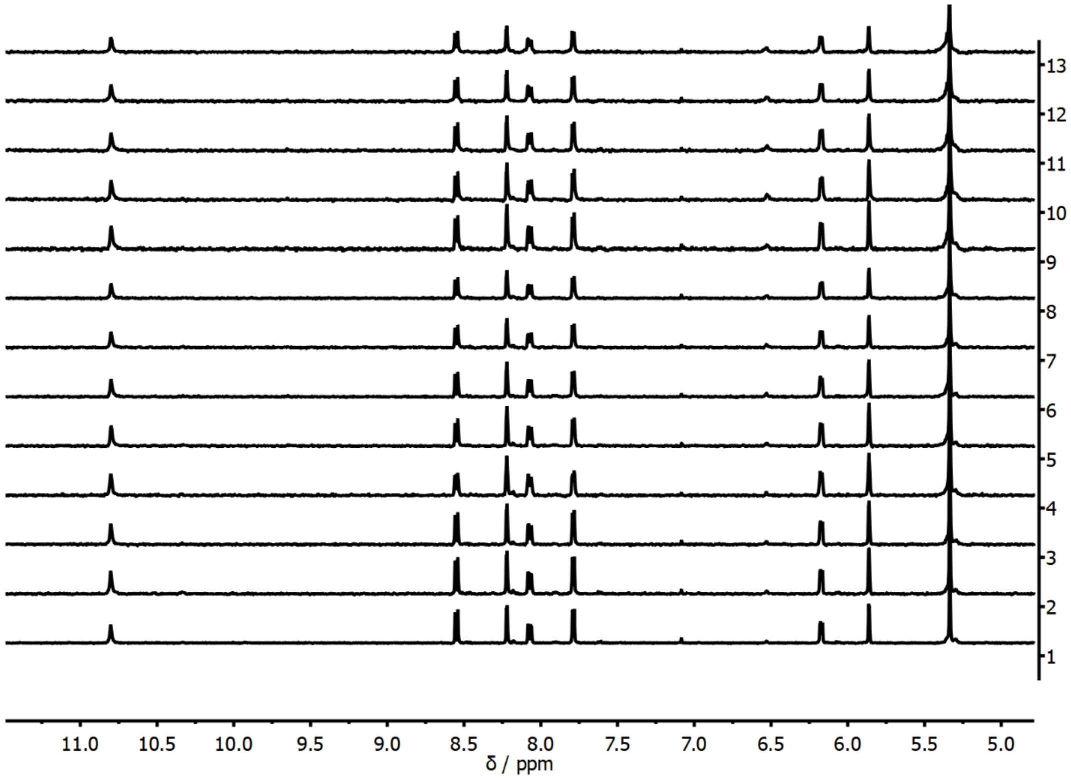

Figure S.80 – Stack plot for the NMR dilution of in *n*-octane with concentration ranging from 0.647 mM (bottom) to 0.114 mM (top)

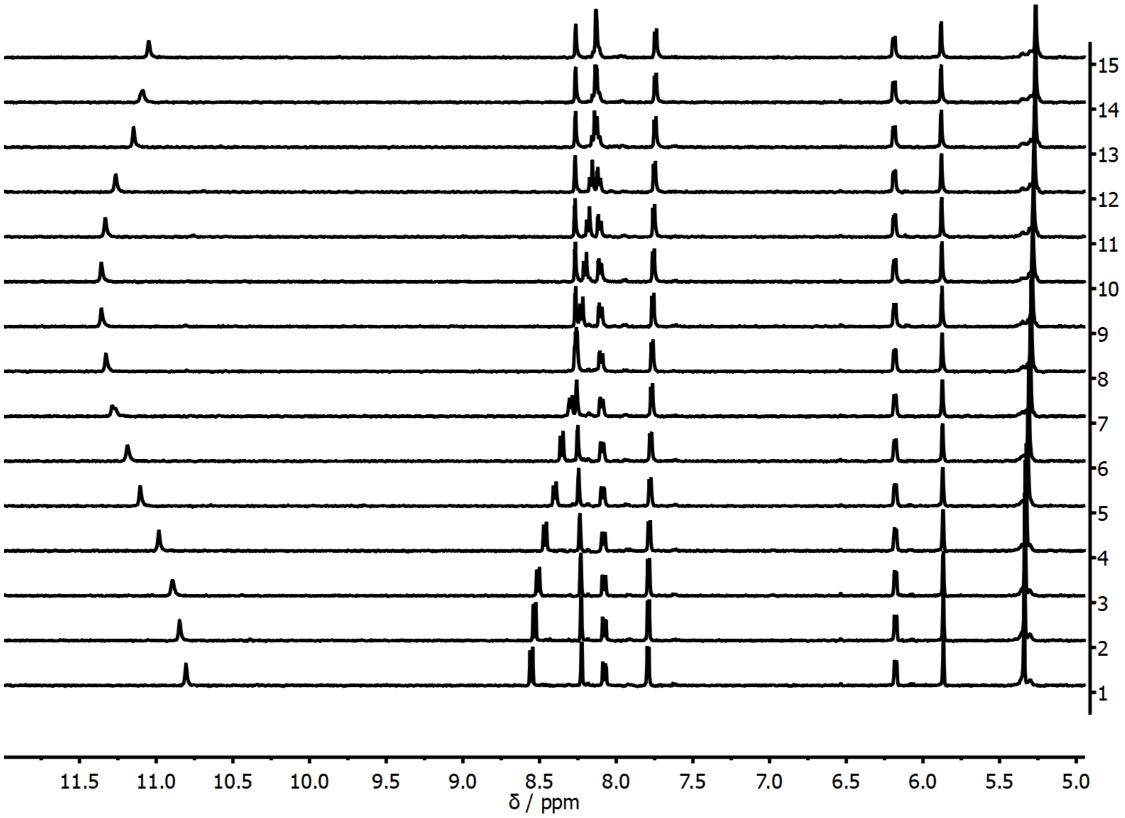

Figure S.81 – Stack plot for the NMR titration of PFTB into **15** (0.647 mM) in *n*-octane at 298 K.

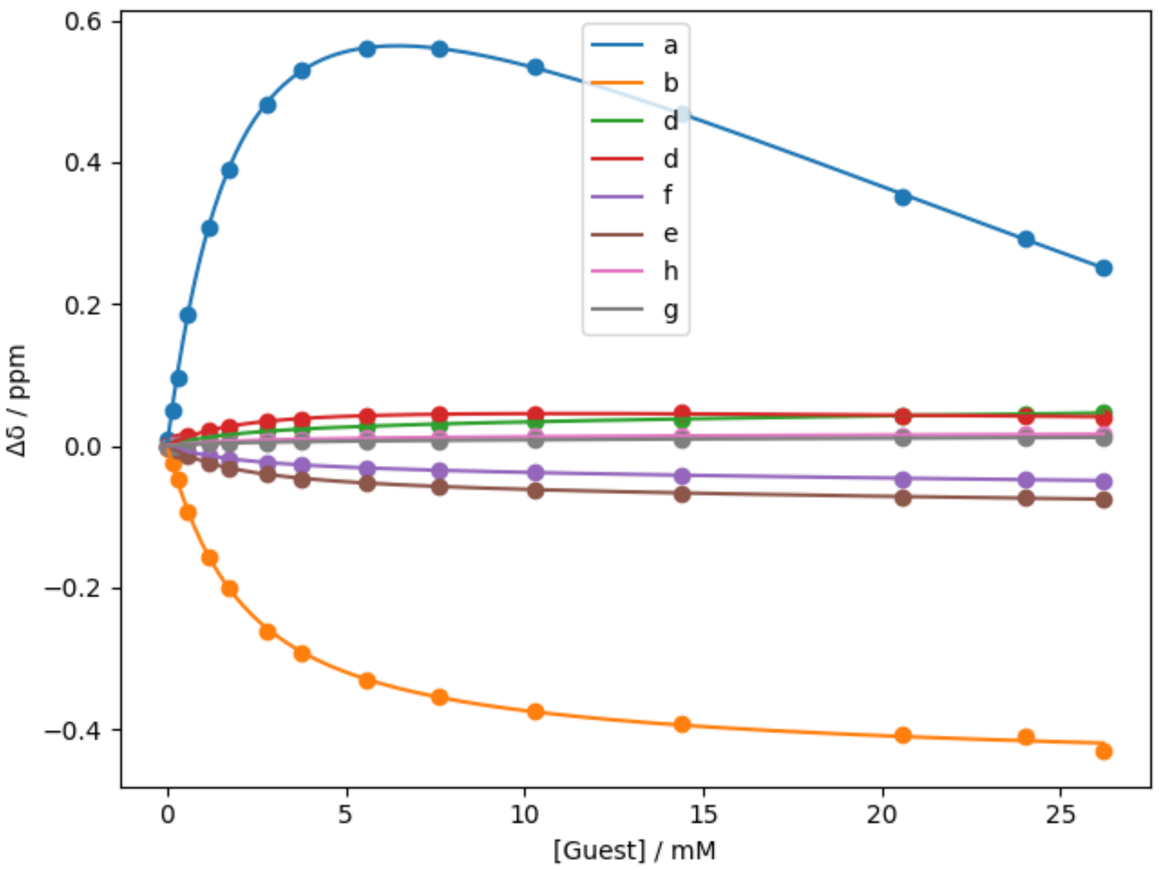

Figure S.82 – Fit for the NMR titration of PFTB into **15** (0.647 mM) to a 1:2 binding isotherm in *n*-octane at 298 K.

NMR Titration Shift Changes

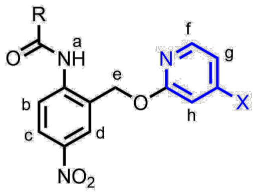

Figure S.83 - Protons labelled for the change in chemical shift in the free species and in the species bound to PFTB.

Number	X	Δδ(a)	Δδ(b)	Δδ(c)	Δδ(d)	Δδ(e)	Δδ(f)	Δδ(g)	Δδ(h)
**1**	N/A	0.22	-0.42	0.06	0.03	N/A	N/A	N/A	N/A
**10**	CN	0.49	-0.37	0.03	0.06	-0.03	-0.03	0.03	0.04
**11**	CF_3	0.66	-0.36	0.02	0.04	-0.05	-0.03	0.02	0.02
**12**	H	0.93	-0.44	0.03	0.06	-0.05	-0.04	0.02	0.03
**13**	Me	0.74	-0.40	0.05	0.05	-0.05	-0.05	0.04	0.03
**14**	OMe	0.55	-0.34	0.01	0.04	-0.02	0.02	0.02	-0.04
**15**	NMe_2	0.88	-0.43	0.03	0.06	-0.04	-0.07	0.01	0.01

Table S.4 A table of the shift change for the protons above in Figure S.2 from the free species to the limiting shift upon 1:1 complexation with PFTB. Protons f, g and h don't exist in species **15** as the pyridyloxy group is replaced by a methyl group

Number	X	Complex	Δδ(a)	Δδ(b)	Δδ(c)	Δδ(d)	Δδ(e)	Δδ(f)	Δδ(g)	Δδ(h)
**12**	H	Free	9.87	8.56	8.09	8.25	5.41	8.12	6.83	6.72
		1:1	10.80	8.12	8.12	8.31	5.36	8.08	6.85	6.76
		1:2	10.19	8.15	8.16	8.32	5.32	8.11	6.92	6.80
**15**	NMe_2	Free	10.80	8.57	8.06	8.22	5.34	7.79	6.17	5.86
		1:1	11.67	8.14	8.10	8.28	5.27	7.75	6.18	5.88
		1:2	8.03	8.06	8.19	8.19	5.19	7.67	6.21	5.90

Table S.5 A table of the shifts of the free host, 1:1 host:guest complex with PFTB, and 1:2 host:guest complexes with PFTB for compounds **12** and **15** from NMR titrations.

For compounds **1, 12** and **15** the limiting complexation-induced changes in chemical shift were found by directly fitting the NMR titration data (see Figures S.53, S.71, and S.83). For the other compounds, the limiting complexation-induced changes in chemical shift for the 1:1 complexes were estimated by comparing the chemical shifts for the free host (0.20 mM to 1.0 mM) with the chemical shifts in the presence of PFTB (45-50 mM). Assuming that the amount of 1:2 complex is negligible at these concentrations, the chemical shifts for the 1:1 complex were determined using Equation 2 and the association constant for the 1:1 complex measured from the UV/Vis titration.[4]

$$\frac{\delta_{obs}-\delta_H}{\delta_{HG}-\delta_H}=\frac{[HG]}{[H]_0}=\frac{(K([H]_0+[G]_0)+1)\pm\sqrt{(K([H]_0+[G]_0)+1)^2-4K^2[H]_0[G]_0}}{2K[H]_0}$$

Equation 2

δ_{obs}	Observed chemical shift
δ_H	Free host chemical shift
δ_{HG}	Bound host chemical shift
[HG]	Host-guest complex concentration
$[H]_0$	Total host concentration
$[G]_0$	Total guest concentration
K	Association constant

Table S.6 – Definitions of the terms in Equation 2.

Computational calculations

All molecular mechanics calculations were done using Schrödinger's Maestro software (2016 Edition), with $CHCl_3$ as the implicit solvent and MMFFs as the forcefield of choice.[5] First a minimisation calculation was ran (10000 iterations, convergence on gradient of 0.01). The minimisation result was then used as the basis for a conformational search (mixed torsional/low mode sampling, 10000 steps). The result of this was then used as a basis for a Jaguar DFT optimisation using a 6-31G* basis set[6-8], a B3LYP functional[9-12] and a nonrelativistic Hamiltonian. The calculations were done in the gas phase. The output of this was then used to calculate the molecular electrostatic potential (MEP) on the 0.0300 e $bohr^{-3}$ electron density isosurface in NWChem 7. The MEP was then converted to H-bond parameters using purpose-built Python and JavaScript programmes.[13]

References

1. Fulmer, G.R., Miller, A.J.M., Sherden, N.H., Gottlieb, H.E., Nudelman, A., Stoltz, B.M., Bercaw, J.E., Goldberg, K.I., *Organometallics,* **2010**, 29, 2176-2179
2. Hansch, C., Rockwell, S.D., Low, P.Y.C., Leo, A., and Steller, E.E., J. Med. Chem., **1977**, 20, 304-306
3. Berthelot, M., Laurence, C., Safar, M., and Besseau, F., *J. Chem. Soc., Perkin Trans. 2,* **1998**, 283-290
4. Anslyn, E.V, Dougherty, D.A., *Modern Physical Organic Chemistry*, **2005**, University Science Books, 220
5. Halgren, T.A., *J. Comput. Chem.*, **1999**, 20, 720-729
6. Ditchfield, R., Hehre, W. J., Pople, J. A., *J. Chem. Phys.*, **1971**, 54, 724-728
7. Hehre, W. J., Ditchfield, R., Pople, J. A., *J. Chem. Phys.*, **1972**, 56, 2257-2261
8. Hariharan, P.C., Pople, J.A., *Theor. Chim. Acta.*, **1973**, 28, 213-222
9. Becke, A.D., *J. Chem. Phys.*, **1993**, 98, 5648-5652
10. Lee, C., Yang, W., Parr, R.G., *Phys. Rev. B*, **1988**, 37, 785-789
11. Vosko, S.H., Wilk, L., Nusair, M., *Can. J. Phys.*, **1980**, 58, 1200-1211
12. Stephens, P.J., Devlin, F.J., Chabalowski, C.F., Frisch, M.J., *J. Phys. Chem*., **1994**, 98, 11623-11627
13. M. C. Storer and C. A. Hunter, *Phys. Chem. Chem. Phys.*, 2022, **24**, 18124
